# Synthesis and Characterization of New Pyrano[2,3-*c*]pyrazole Derivatives as 3-Hydroxyflavone Analogues

**DOI:** 10.3390/molecules28186599

**Published:** 2023-09-13

**Authors:** Arminas Urbonavičius, Sonata Krikštolaitytė, Aurimas Bieliauskas, Vytas Martynaitis, Joana Solovjova, Asta Žukauskaitė, Eglė Arbačiauskienė, Algirdas Šačkus

**Affiliations:** 1 Department of Organic Chemistry, Kaunas University of Technology, Radvilėnų pl. 19, LT-50254 Kaunas, Lithuania; arminas.urbonavicius@ktu.lt (A.U.); sonata.krikstolaityte@ktu.lt (S.K.); vytas.martynaitis@ktu.lt (V.M.); joana.solovjova@ktu.lt (J.S.); asta.zukauskaite@upol.cz (A.Ž.); 2 Institute of Synthetic Chemistry, Kaunas University of Technology, K. Baršausko g. 59, LT-51423 Kaunas, Lithuania; aurimas.bieliauskas@ktu.lt; 3 Department of Chemical Biology, Palacký University, Šlechtitelů 27, CZ-78371 Olomouc, Czech Republic

**Keywords:** pyrazoles, pyrano[2,3-*c*]pyrazoles, 3-hydroxyflavone, Algar–Flynn–Oyamada reaction, NMR investigation, ESIPT

## Abstract

In this paper, an efficient synthetic route from pyrazole-chalcones to novel 6-aryl-5-hydroxy-2-phenylpyrano[2,3-*c*]pyrazol-4(2*H*)-ones as 3-hydroxyflavone analogues is described. The methylation of 5-hydroxy-2,6-phenylpyrano[2,3-*c*]pyrazol-4(2*H*)-one with methyl iodide in the presence of a base yielded a compound containing a 5-methoxy group, while the analogous reaction of 5-hydroxy-2-phenyl-6-(pyridin-4-yl)pyrano[2,3-*c*]pyrazol-4(2*H*)-one led to the zwitterionic 6-(*N*-methylpyridinium)pyrano[2,3-*c*]pyrazol derivative. The treatment of 5-hydroxy-2,6-phenylpyrano[2,3-*c*]pyrazol-4(2*H*)-one with triflic anhydride afforded a 5-trifloylsubstituted compound, which was further used in carbon–carbon bond forming Pd-catalyzed coupling reactions to yield 5-(hetero)aryl- and 5-carbo-functionalized pyrano[2,3-*c*]pyrazoles. The excited-state intramolecular proton transfer (ESIPT) reaction of 5-hydroxypyrano[2,3-*c*]pyrazoles from the 5-hydroxy moiety to the carbonyl group in polar protic, polar aprotic, and nonpolar solvents was observed, resulting in well-resolved two-band fluorescence. The structures of the novel heterocyclic compounds were confirmed by ^1^H-, ^13^C-, ^15^N-, and ^19^F-NMR spectroscopy, HRMS, and single-crystal X-ray diffraction data.

## 1. Introduction

Fused pyrazole derivatives represent an important class of organic compounds as they are found in a large number of biologically and chemically active compounds [1]. These compounds are known for their anticancer [2], antimicrobial [3], antiviral [4], and anti-coagulant properties [5], and for their activity against CNS disorders [6]. Some of the fused pyrazole moieties are present in marketed drugs, such as apixaban, sildenafil, indiplon, zaleplon, etazolate, cartazolate, allopurinol, and futibatinib, which was recently approved by the U.S. Food and Drug Administration (FDA) [7].

Among other fused systems, pyrano[2,3-*c*]pyrazoles have been investigated for analgesic and anti-inflammatory [8], antimicrobial [9,10], and anticancer [11] activities. Recently, Sun et al. described the nano-formulation and anticancer activity of a 6-amino-4-(2-hydroxyphenyl)-3-methyl-1,4-dihydropyrano[2,3-*c*]pyrazole-5-carbonitrile via the blocking of the cell cycle through a p53-independent pathway [12], while Nguyen et al. reported a four-component sulfonated amorphous carbon and eosin Y-catalyzed synthesis and the molecular docking of 6-amino-1,4- or 2,4-dihydropyrano[2,3-*c*]pyrazole-5-carbonitriles as inhibitors of p38 MAP kinase [13]. In our previous studies, we reported the synthesis, characterization, and biological evaluation of several pyrano[2,3-*c*]pyrazole derivatives [14,15]. However, 5-hydroxy-2,6-diarylpyrano[2,3-*c*]pyrazol-4(2*H*)-ones, which can serve as potential analogues of 3-hydroxyflavone, are still understudied.

**Figure 1:**
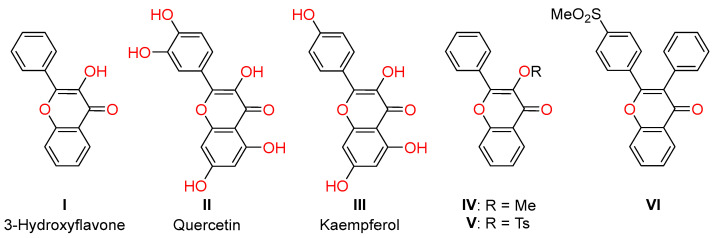
Structural formulas of selected 3-hydroxyflavones and their relative compounds.

3-Hydroxyflavone **I** (Figure 1) is known as the backbone of all flavonols. Flavonols are a class of the flavonoid family, a group of naturally occurring substances with variable phenolic structures, found in fruits, vegetables, grains, bark, roots, stems, flowers, tea, and wine [16,17,18]. Quercetin **II** and kaempferol **III** (Figure 1) are the most prevalent in plants and are among the flavonols that have been most investigated and reviewed for beneficial health properties, such as antioxidant, antimicrobial, hepatoprotective, and anti-inflammatory properties, and other effects [19,20]. Synthetic and semisynthetic flavonol derivatives have been reported in the literature in an attempt to improve the biochemical and pharmacological properties of their corresponding natural compounds. For example, synthesis and anti-*Leishmania* activity were reported for benzothiophene-flavonols [21]. A series of spirochromone-flavonols [22] and thiophene-pyrazole-flavonols [23] was synthesized and tested as antimicrobial agents. In addition, flavonols containing an isothiazolidine ring have been found to be effective inhibitors of cyclin-dependent kinase 2 (CDK2) [24].

3-Hydroxyflavones are known as fluorescent dyes because of their typical excited-state intramolecular photon transfer (ESIPT). ESIPT is one type of proton transfer reaction that has been the subject of considerable interest and a number of investigations in recent decades [25,26]. 3-Hydroxyflavones have been investigated as therapeutic imaging agents, including as fluorescence sensors and probes for the detection of the microenvironment, metal ions, and structures of proteins and DNA [27,28,29,30]. For example, Jiang et al. reported the application of 3-hydroxyflavone-based ESIPT fluorescent dyes for the dynamic imaging of lipid droplets with cells and tissues [31]. In a study by Kamariza et al., a 3-hydroxychromone derivative, 2-[7-(diethylamino)-9,9-dimethyl-9*H*-fluoren-2-yl]-3-hydroxy-4*H*-chromen-4-one, was conjugated to trehalose and a bright solvatochromic dye was obtained that detects *Mycobacterium tuberculosis* in a matter of minutes [32].

The *O*-methylation of 3-hydroxyflavones with reagents such as diazomethane, methyl iodide, dimethyl sulfate, or dimethyl carbonate proceeded to give *O*-methylated flavonoids, which exhibited a variety of biological activities [33,34,35]. For example, Ohtani et al. investigated the effect of 3-methoxyflavone derivatives, such as those of compound **IV** (Figure 1), on *P*-glycoprotein by measuring the potentiation of cellular accumulation and growth inhibition [36]. Juvale et al. reported the inhibitory activity of 3-methoxyflavones against a breast cancer resistance protein (BVRP/ABCG2) [37]. Furthermore, 3-hydroxyflavone treated with *p*-TsCl in the presence of a base afforded a corresponding flavone tosylate **V**, which was used in a Suzuki–Miyaura reaction for cross coupling with various phenyl boronic acids to give 2,3-diarylbenzopyrans [38,39]. Flavone-like 2,3-diarylbenzopyrans, such as compound **VI** (Figure 1), have been synthesized as novel selective inhibitors of cyclooxygenase-2 [40,41]. 

In the continuation of our research on the development of novel fused heterocyclic pyrazole-containing systems, we report here the synthesis, structural elucidation, and optical properties of novel 6-aryl-5-hydroxy-2-phenylpyrano[2,3-*c*]pyrazol-4(2*H*)-one derivatives as analogues of 3-hydroxyflavones. The ESIPT reaction of 6-aryl-5-hydroxy-2-phenylpyrano[2,3-*c*]pyrazol-4(2*H*)-ones from the 5-hydroxy moiety to the carbonyl group in MeOH and polar aprotic and non-polar solvents was also investigated. The obtained 5-hydroxy-2-phenylpyrano[2,3-*c*]pyrazol-4(2*H*)-ones were further functionalized by methylation as well as the Pd-catalyzed Suzuki, Heck, and Sonogashira coupling reactions of intermediate 5-triflate.

## 2. Results and Discussion

### 2.1. Chemistry

**Scheme 1:**
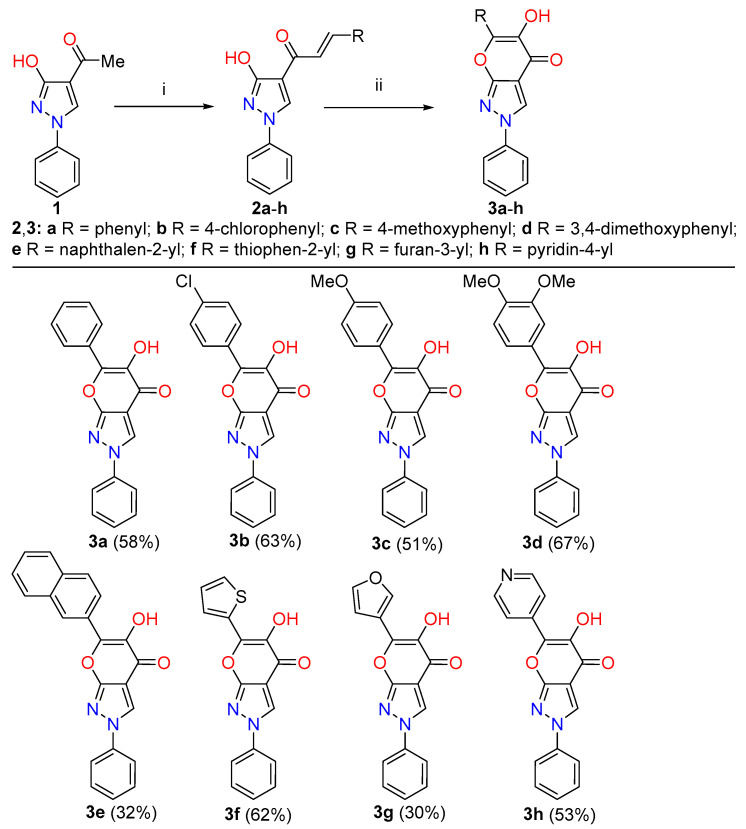
Reagents and conditions: (i) appropriate carbaldehyde, NaOH, EtOH, 55 °C, 3–5 h, in accordance with ref. [44]; (ii) NaOH, EtOH, H_2_O_2_, −25 °C, 2 h, then rt, 16 h.

The synthesis of 6-(hetero)aryl-5-hydroxy-2-phenylpyrano[2,3-*c*]pyrazol-4(2*H*)-ones **3a**–**h** was carried out as depicted in Scheme 1. 1-Phenyl-1*H*-pyrazol-3-ol **1** was obtained using a previously reported method [42,43] and subjected to a Claisen–Schmidt condensation reaction with variously 4′-substituted (hetero)aryl aldehydes in the presence of ethanolic sodium hydroxide, as we have described [44]. Heating the reaction mixture at 55 °C for 3 to 5 h afforded (*E*)-1-(3-hydroxy-1-phenyl-1*H*-pyrazol-4-yl)prop-2-en-1-ones **2a**–**h** in poor to excellent yields (36–95%). 

In a subsequent step, an Algar–Flynn–Oyamada (AFO) synthetic approach was applied for the formation of novel pyrano[2,3-*c*]pyrazol-4(2*H*)-ones **3a**–**h**. The AFO reaction is a stepwise process whereby chalcones undergo an oxidative cyclization to form flavones in the presence of alkaline hydrogen peroxide [45]. The AFO reaction outcome is dependent on the choice of the base; therefore, chalcone **2a** was used as a model compound for the fine-tuning of the reaction conditions. Several organic and inorganic bases (NaOH, KOH, NaOAc, TEA, and NaHCO_3_) were screened in different mixtures of ethanol/water as a solvent and a divergent amount of hydrogen peroxide (Table S1). The best result was obtained when using NaOH in EtOH and employing 5 eq of H_2_O_2_. Stirring chalcones **2a**–**h** with hydrogen peroxide in an alkaline ethanolic solution at −25 °C for 2 h and at room temperature overnight afforded the flavonol analogues **3a**–**h** in poor to good yields (30–67%). The pyrano[2,3-*c*]pyrazol-4(2*H*)-ones **3e** and **3g** were obtained in lower yields (30–32%) when chalcones bearing naphtalen-2-yl or furan-3-yl substituents (**2e** and **2g**, respectively) were used as starting materials in the AFO reaction. Unfortunately, the AFO reaction of (*E*)-3-(4-fluorophenyl)-1-(3-hydroxy-1-phenyl-1*H*-pyrazol-4-yl)prop-2-en-1-one or its 3-(4-nitrophenyl) counterpart gave only traces of targeted pyrano[2,3-*c*]pyrazol-4(2*H*)-ones.

**Figure 2:**
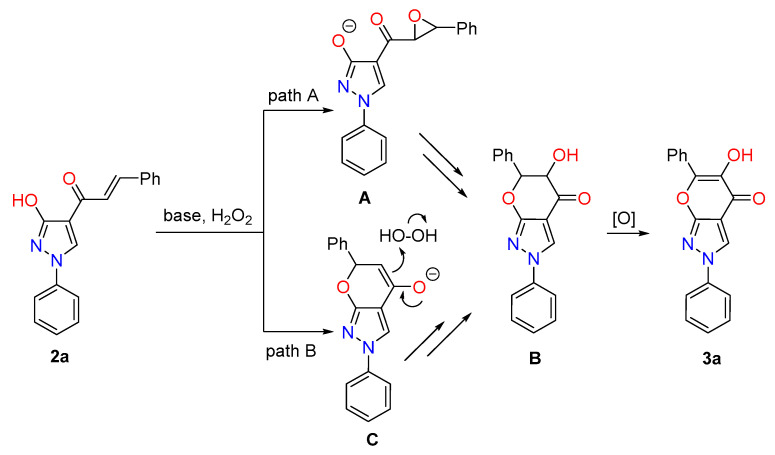
Mechanisms proposed for the transformation reaction of compound **2a** to compound **3a**.

According to the mechanistic studies reported in the literature [45,46,47], the formation of 6-(hetero)aryl-5-hydroxy-2-phenylpyrano[2,3-*c*]pyrazol-4(2*H*)-ones **3** from (*E*)-1-(3-hydroxy-1-phenyl-1*H*-pyrazol-4-yl)prop-2-en-1-ones **2**, employing AFO reaction conditions could proceed according to two different pathways, as depicted in Figure 2, using the transformation of **2a** to **3a** as an example. According to the approach suggested by Shen et al., first epoxide **A** (Figure 2, path A) is formed; then it is subsequently cyclized to 5-hydroxy-2,6-diphenyl-5,6-dihydropyrano[2,3-*c*]pyrazol-4(2*H*)-one **B** and oxidized to target 5-hydroxy-2-phenylpyrano[2,3-*c*]pyrazol-4(2*H*)-one **3a** [45]. Alternatively, as suggested by Ferreira et al., pyrazole-chalcone **2a** might first undergo a cyclization forming a 2,6-diphenyl-2,6-dihydropyrano[2,3-*c*]pyrazol-4-olate **C** (Figure 2, path B), followed by an attack of hydrogen peroxide and subsequent oxidation to form **3a** [47]. 

**Scheme 2:**
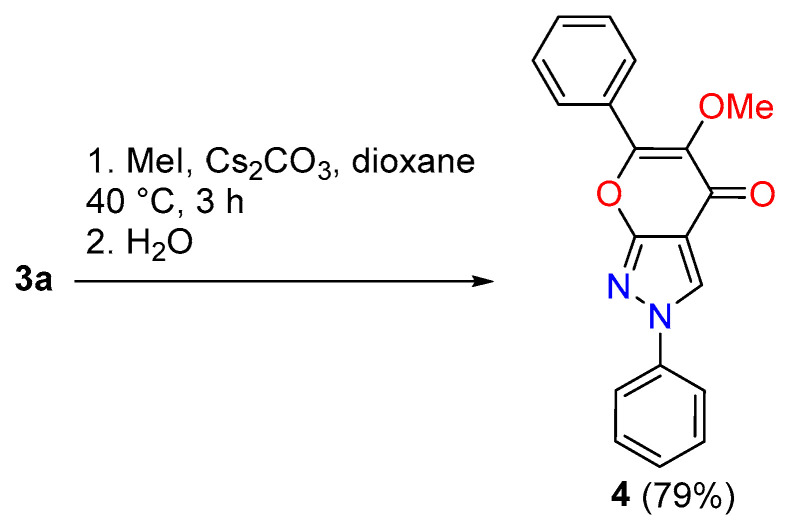
*O*-Methylation of compound **3a**.

For further modification of the obtained flavanol analogue **3a**, *O*-alkylation reaction conditions were applied. As a result, treating **3a** with methyl iodide in the presence of cesium carbonate in dioxane at 40 °C gave *O*-methylated compound **4** in a 79% yield (Scheme 2).

**Scheme 3:**
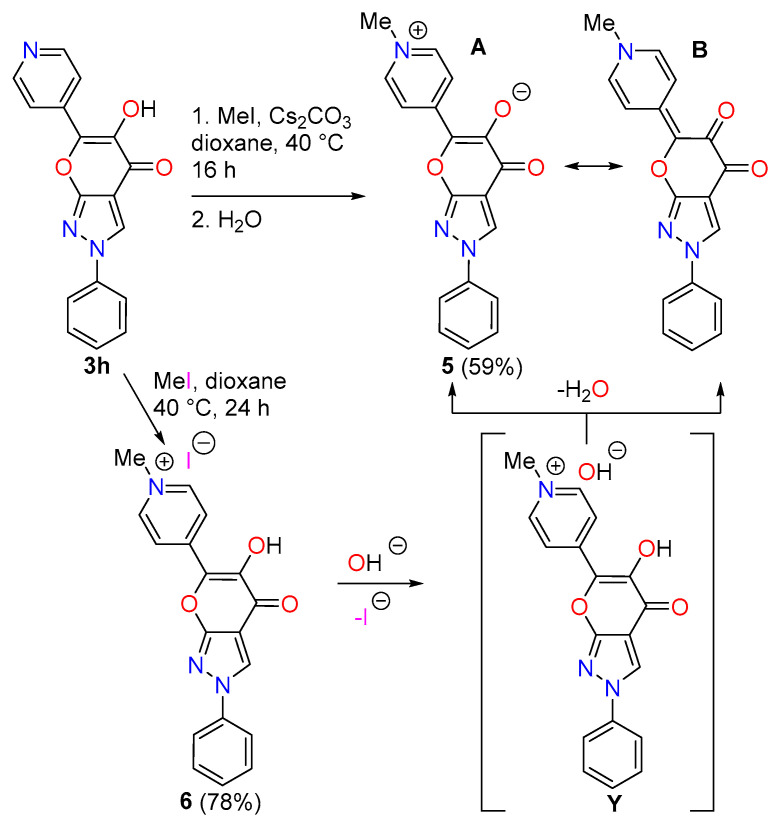
*N*-Methylation of compound **3h**.

Subsequently the methylation of compound **3h** containing both the hydroxyl group and the pyridin-4-yl substituent was investigated (Scheme 3). With the alkylation reaction conditions described above (MeI, Cs_2_CO_3_, dioxane, 40 °C), a formation of zwitterionic pyrano[2,3-*c*]pyrazol derivative **5** as the main product was observed. 

The proposed mechanism for the formation of compound **5** is shown in Scheme 3. Presumably, first, as a result of the reaction of pyridinyl-containing compound **3h** with methyl iodide, methylpyridinium iodide **6** was formed. This was also demonstrated when alkylating compound **3h** in the absence of a base as salt **6** was obtained in a 78% yield. The subsequent treatment of methylpyridinium iodide **6** with a base led to the formation of methylpyridinium hydroxide **Y**, which, upon the removal of the water molecule, led to the formation of the corresponding structure **5** as a resonance hybrid with the two contributing forms **A** and **B**, zwitterionic and neutral molecular structures, respectively. Pat et al. investigated the two-photon absorption (TPA) processes in a class of 4-quinopyran chromophores. The neutral molecular structure with a quinoid geometry is the molecular ground state, while the zwitterionic configuration with a benzenoid structure contributes significantly. The bond connecting the donor and acceptor phenylene fragments is a double bond when the molecule is neutral, while it is a single bond for the zwitterionic structure [48].

**Scheme 4:**
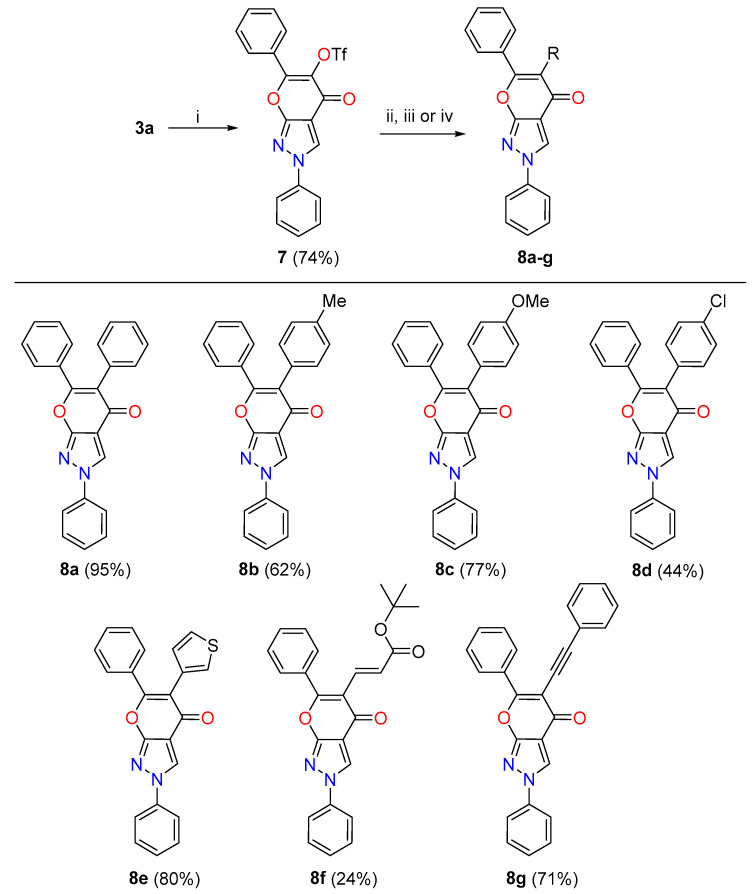
Reagents and conditions: (i) TEA, Tf_2_O, DCM, 0–24 °C, 16 h; (ii) (hetero)aryl boronic acid, K_3_PO_4_, KBr, Pd(PPh_3_)_4_, dioxane, 90 °C, 16 h (for **8a**–**e**); (iii) *tert*-butyl acrylate, TEA, Pd(PPh_3_)_2_Cl_2_, DMF (dry), 100 °C (for **8f**), 72 h; (iv) phenylacetylene, TEA, CuI, Pd(PPh_3_)_2_Cl_2_, DMF (dry), 65 °C, 1 h (for **8g**).

Further functionalization of pyrano[2,3-*c*]pyrazol-4(2*H*)-ones was accomplished via *O*-triflate intermediate **7**, which was synthesized from 5-hydroxy-2,6-diphenylpyrano[2,3-*c*]pyrazol-4(2*H*)-one (**3a**) following a standard procedure using Tf_2_O in the presence of TEA (Scheme 4). The obtained 4-oxo-2,4-dihydropyrano[2,3-*c*]pyrazol-5-yl trifluoromethanesulfonate **7** was subjected to Suzuki, Heck, and Sonogashira reactions to examine the employment of Pd-catalyzed coupling reactions for the functionalization of pyrano[2,3-*c*]pyrazol-4(2*H*)-ones. Triflate **7** underwent Suzuki-type cross coupling with (hetero)aryl boronic acids to give compounds **8a**–**e** in fair to excellent yields (44–95%). In the course of this coupling, standard conditions were applied, i.e., Pd(PPh_3_)_4_ was used as a catalyst and anhydrous K_3_PO_4_ as a base in dioxane at 90 °C. The reaction was carried out in the presence of KBr, which is known to suppress the decomposition of the palladium catalyst transition state by converting phosphonium salts to palladium bromide [49]. The Suzuki reaction yield was lower (44%) when 4-chlorophenylboronic acid was used for the cross coupling.

The Heck reaction of triflate **7** and *tert*-butyl acrylate under the standard conditions (Pd(PPh_3_)_2_Cl_2_, TEA, DMF, 100 °C) gave a poor yield (24%) of *tert*-butyl (*E*)-3-(4-oxo-2,4-dihydropyrano[2,3-*c*]pyrazol-5-yl)acrylate **8f**, while the Sonogashira cross-coupling reaction of compound **7** with phenylacetylene under the usual conditions (Pd(PPh_3_)_2_Cl_2_, CuI, TEA, DMF, 65 °C) afforded alkyne **8g** in a good yield (71%). A similar approach of flavonol functionalization employing Pd-catalyzed reactions of *O*-triflates was also reported by Kumar et al. in their study on the synthesis of 3,4-diarylpyrazoles and 4,5-diarylpyrimidines, starting with triarylbismuth as a three-fold arylating reagent and 3-trifloxychromones [50]. Dahlén et al. reported a synthetic strategy to form 2,3,6,8-tetrasubstituted chromone derivatives employing a Stille coupling reaction for the functionalization of the third position of the ring via intermediate 4-oxo-4*H*-chromen-3-yl trifluoromethanesulfonates [51]. Notably, it was observed that the latter compounds were not active under Heck reaction conditions. In addition, Akwari et al. demonstrated effective 3-arylation of flavones via a Suzuki cross-coupling reaction of 3-(trifluorosulphonyloxy)flavone [52]. 

### 2.2. NMR Spectroscopic Investigations

**Figure 3:**
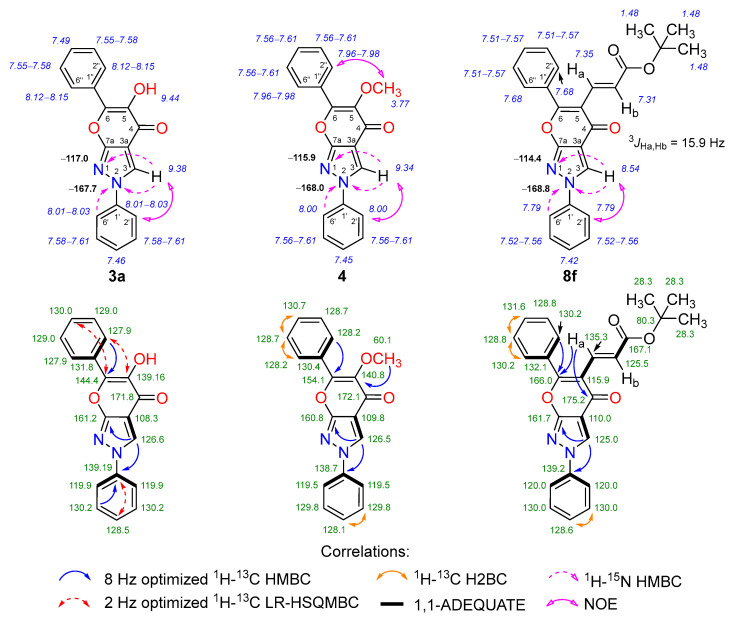
Relevant ^1^H-^13^C HMBC, ^1^H-^13^C LR-HSQMBC, ^1^H-^13^C H2BC, ^1^H-^15^N HMBC, ^1^H-^1^H NOESY, and 1,1-ADEQUATE correlations, as well as ^1^H NMR (italics), ^13^C NMR, and ^15^N NMR (bold) chemical shifts of compounds **3a** (DMSO-*d*_6_), **4** (DMSO-*d*_6_), and **8f** (CDCl_3_).

**Figure 4:**
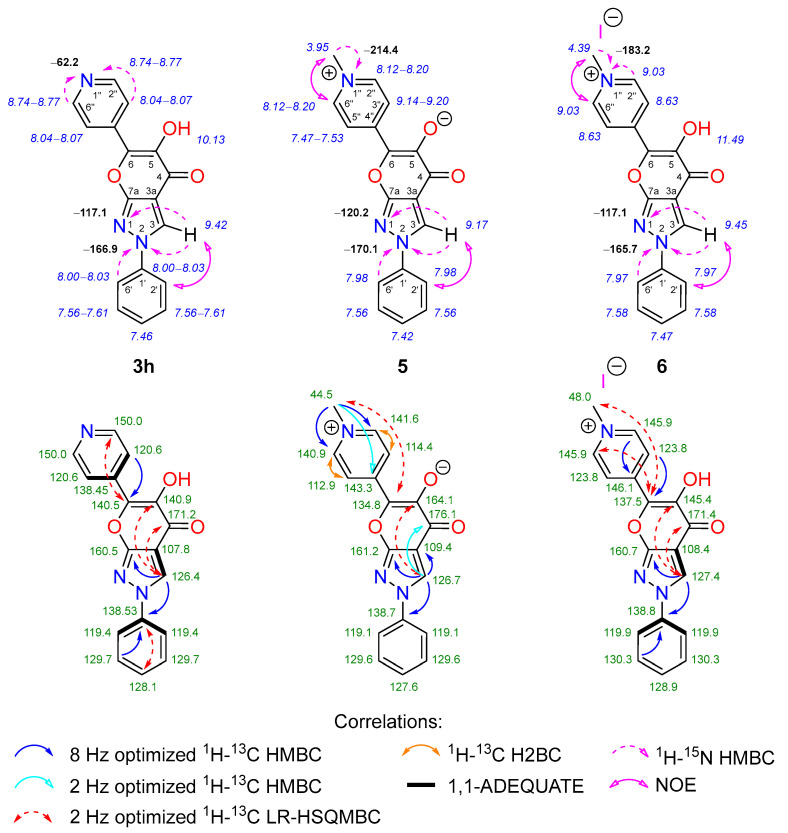
Relevant ^1^H-^13^C HMBC, ^1^H-^13^C LR-HSQMBC, ^1^H-^13^C H2BC, ^1^H-^15^N HMBC, ^1^H-^1^H NOESY, and 1,1-ADEQUATE correlations, as well as ^1^H NMR (italics), ^13^C NMR, and ^15^N NMR (bold) chemical shifts of compounds **3h** (DMSO-*d*_6_), **5** (DMSO-*d*_6_), and **6** (DMSO-*d*_6_).

The formation of 6-(hetero)aryl-5-hydroxy-2-phenylpyrano[2,3-*c*]pyrazol-4(2*H*)-ones **3a**–**h** and their derivatives **4**, **5**, **6**, **7**, and **8a**–**g** was confirmed through detailed analysis of their spectroscopic data. Key information for structure elucidation was obtained from NMR spectral data using a combination of standard and advanced NMR spectroscopy techniques, such as ^1^H-^13^C HMBC, ^1^H-^13^C LR-HSQMBC, ^1^H-^15^N HMBC, ^1^H-^13^C HSQC, ^1^H-^13^C H2BC, ^1^H-^1^H COSY, ^1^H-^1^H TOCSY, ^1^H-^1^H NOESY, and 1,1-ADEQUATE experiments. Since popular NMR prediction programs such as CSEARCH, ACD C+H predictor, as well as NMR chemical shift databases for structural dereplication depend on high-quality data with unambiguously assigned resonances [53], we carried out NMR studies with the obtained compounds to fully map all the ^1^H, ^13^C and ^15^N NMR signals as accurately as possible. The corresponding NMR data for the selected representatives of the aforementioned new ring systems are displayed in Figure 3 and Figure 4.

An initial comparison of the ^1^H NMR spectra between chalcone **2a** and compound **3a**, which was isolated as the sole product, clearly indicated the disappearance of characteristic olefinic protons (δ 7.63 and 7.75 ppm) from the prop-2-en-1-one moiety. Furthermore, the ^13^C NMR and DEPT, along with the ^1^H-^13^C HSQC spectroscopic data of **3a**, revealed the presence of two new quaternary carbons (δ 139.16 and 144.4 ppm) in the absence of two olefinic methine carbons, clearly indicating a successful oxidative cyclization to flavanol. The structure of the pyrano[2,3-*c*]pyrazol-4(2*H*)-one ring system **3a** bearing phenyl substituents at sites N-2 and C-6 was further elucidated via the connectivities based on the through-space correlations from the ^1^H-^1^H NOESY spectrum. In this case, distinct NOEs were exhibited between the pyrazole ring proton 3-H (singlet, δ 9.38 ppm) and the neighboring phenyl group 2′(6′)-H protons (δ 8.01–8.03 ppm), which confirms their proximity in space. The pyrazole 3-H proton was easily distinguished as it exhibited not only long-range HMBC correlations with neighboring N-2 “pyrrole-like” (δ −167.7 ppm) and N-1 “pyridine-like” (δ −117.0 ppm) nitrogen atoms, but also HMBC correlations with the quaternary carbons C-3a (δ 108.3 ppm) and C-7a (δ 161.2 ppm), respectively. The quaternary carbons C-5 (δ 139.16 ppm) and C-6 (δ 144.4 ppm) were assigned by comparing the long-range correlations obtained from the ^1^H-^13^C HMBC and ^1^H-^13^C LR-HSQMBC spectra. The most downfield and significantly broadened ^1^H signal resonating at δ 9.44 ppm was attributed to the hydroxyl group as it lacked correlations in the HSQC spectra. Finally, by process of elimination, the most downfield ^13^C signal resonating at δ 171.8 ppm was confidently assigned to the carbonyl carbon, thus completing our assignment of the pyrano[2,3-*c*]pyrazol-4(2*H*)-one ring system. An in-depth analysis of NMR spectral data showed that the chemical shift values were highly consistent within the flavonol analogues **3a**–**h**, thus validating the shifts for each position (Table S2).

The presence of a hydroxyl group at site 5 was further confirmed by the conversion of **3a** to *O*-methylated and *O*-triflated derivatives **4** and **7**, respectively. While the structural elucidation of *O*-methylated compound **4** was straightforward and followed the same logical approach as in the case of compounds **3a**–**h**, additional distinct NOEs were observed between the methoxy group protons (δ 3.77 ppm) and the neighboring phenyl group 2″(6″)-H protons (δ 7.96–7.98 ppm). The formation of *O*-triflated derivative **7** was clearly distinguished from the ^13^C NMR spectrum, where the CF_3_ group was observed as a quartet at δ 118.2 ppm (q, ^1^*J*_CF_ = 320.8 Hz). Moreover, the ^19^F NMR spectrum revealed a chemical shift of the CF_3_ group at δ −74.0 ppm, which is in good agreement with the data reported in the literature [54,55]. The triflate intermediate **7** underwent Pd-catalyzed coupling reactions to give derivatives **8a**–**g**, whose structures were also unambiguously elucidated. For instance, compound **8f** was obtained as an *E*-isomer. The magnitude of the vicinal coupling between the olefinic protons H_a_ (δ 7.35 ppm) and H_b_ (δ 7.31 ppm), which exhibited an AB-spin system and appeared as two sets of doublets (^3^*J*_Ha,Hb_ = 15.9 Hz), unquestionably confirmed *E*-configuration at the C=C double bond. Lastly, the olefinic protons were easily discriminated as only the proton H_a_ exhibited long-range ^1^H-^13^C HMBC correlations with neighboring C-4, C-5, and C-6 quaternary carbons (Figure 3).

The NMR spectral data of compound 3h with a pyridine moiety at site 6 revealed similar chemical shifts in the pyrano[2,3-*c*]pyrazol-4(2*H*)-one ring system compared with 3a–g. The ^1^H-^15^N HMBC spectrum revealed a new downfield ^15^N signal resonating at δ −62.2 ppm in addition to N-2 “pyrrole-like” (δ −166.9 ppm) and N-1 “pyridine-like” (δ −117.1 ppm) nitrogen atoms from the pyrazole moiety. The formation of methylpyridinium iodide 6 via the alkylation of 3h was unambiguously confirmed from the ^1^H-^15^N HMBC and ^1^H-^1^H NOESY spectral data. For instance, distinct NOEs were observed between the methyl group protons (δ 4.39 ppm) and the neighboring pyridinium 2″(6″)-H protons (δ 9.03 ppm). The aforementioned protons revealed strong long-range correlations with the methylpyridinium nitrogen at δ −183.3 ppm, which is in good agreement with the data reported in the literature [56].

In the case of compound **5**, which can exist in two resonant forms, the ^1^H-^15^N HMBC spectrum revealed a new upfield ^15^N signal resonating at δ −214.4 ppm. Furthermore, distinct ^1^H and ^13^C signals, which appeared to be broadened, were also observed (sites 2″, 3″, 5″, and 6″). Additionally, the key information for the structure elucidation of compounds **3h**, **5**, and **6** was obtained after an in-depth analysis of the long-range correlations in the ^1^H-^13^C HMBC, ^1^H-^13^C H2BC, and ^1^H-^13^C LR-HSQMBC spectra (Figure 4) [57].

**Scheme 5:**
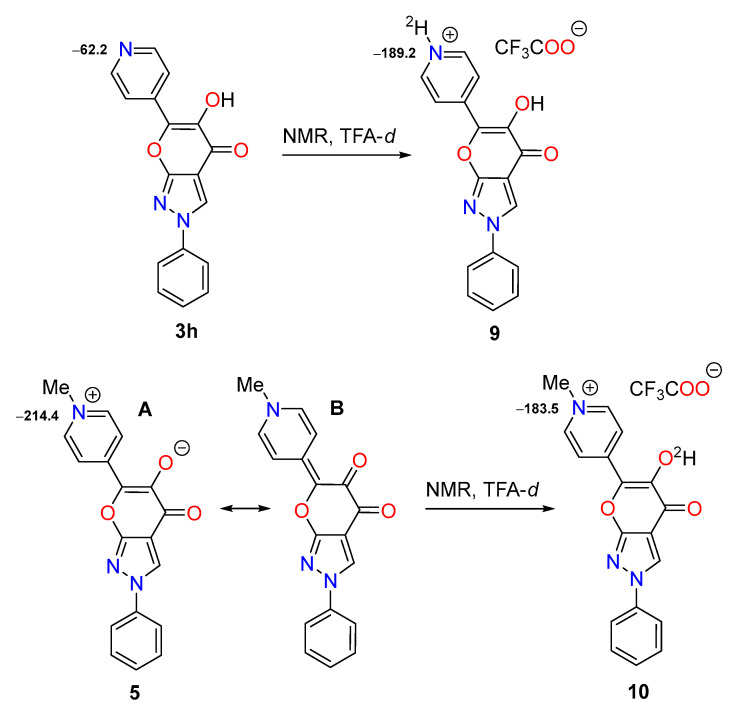
^15^N NMR (bold) chemical shifts of compounds **3h** (DMSO-*d*_6_), **5** (DMSO-*d*_6_), **9** (TFA-*d*), and **10** (TFA-*d*).

Then, we carried out NMR studies of compounds 3h and 5 at 25 °C in TFA-*d* solutions (Scheme 5) to convert them to pyridinium and methylpyridinium trifluoroacetates 9 and 10, respectively. The ^15^N NMR spectral data confirmed that it was easily achieved as “pyridinium-like” ^15^N signals comparable to compound **6** resonating at δ −189.2 ppm and δ −183.2 ppm were observed. Moreover, in the case of compound **10**, which was obtained from compound **5**, the broadening of the ^1^H and ^13^C signals was absent.

### 2.3. Single-Crystal X-ray Diffraction Analysis

**Figure 5:**
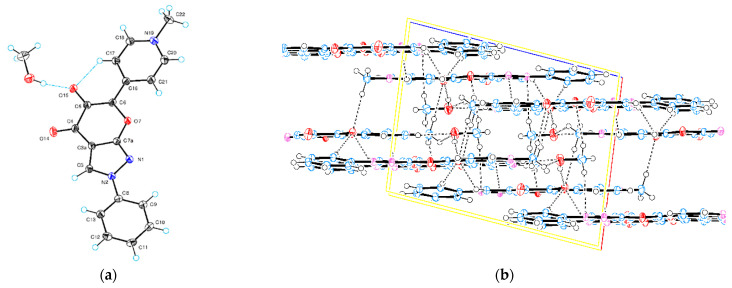
ORTEP view of compound **5**: (**a**) asymmetric unit; (**b**) crystal cell and hydrogen bonds.

The asymmetric molecular structure of compound **5** is shown in Figure 5a. The single crystal is composed of compound **5** solvated with molecules of methanol. The methanol formed hydrogen bonds in the monocrystal of **5**, including the hydrogen link to the O(15) (the H^…^O length is 1.917 Å) (Table S6). The intramolecular hydrogen bond is also observed between the O(15) enolate oxygen and the C(17)–H(17) hydrogen atom (the H^…^O length is 2.207 Å). The main core of compound **5** consists of the planar pyrano[2,3-*c*]pyrazole ring system, which possesses phenyl and the pyridin-4-yl substituents at N(2) and C(6), respectively. These substituents are slightly distorted from the pyrano[2,3-*c*]pyrazole plane. The phenyl ring is turned approx. 10° and the pyridinyl ring for approx. 6° counterclockwise when looking outward from the core. The N(19)–C(22) bond length of the *N*-methylpyridinium moiety is 1.4737(14) Å (Table S9), and the C(17)–C(18) and C(20)–C(21) bond lengths are 1.3721(15) and 1.3633(16) Å, respectively, and agree with the known bond lengths of the *N*-methylpyridynium salts [58]. All atoms of the pyridine moiety are located in the same plane in agreement with the data reported in the literature [59].

The selected bond lengths and angles of the pyrano[2,3-*c*]pyrazole ring are shown in Table 1 and Table 2. The C=O bond length of the pyran-4-one moiety is 1.2265(14) Å which is characteristic of ketone [60]. The C(5)–O(15) bond length [1.2723(13) Å] is shorter than the typical C–O single bond (~1.43 Å) [61], but longer than the typical C=O double bond (~1.23 Å) [62]. It is notable that the C(6)-O(7) bond length [1.4135(12) Å] is longer than that in the O(7)–C(7a) [1.3387(12) Å]. The N(1)–N(2) and N(2)–C(3) bond lengths are 1.3839(12) and 1.3458(14) Å, respectively, and agree with the known bond lengths of pyrazole compounds [63,64,65,66]. The sum of the angles between the covalent bonds around the N(2) atom is 360°, which indicates that a *trigonal planar* geometry exists at the sp^2^-hybridized nitrogen atom. The molecules in the crystal are located in columns made up of asymmetric units held by hydrogen bonds (Figure 5b).

### 2.4. Optical Investigations

**Figure 6:**
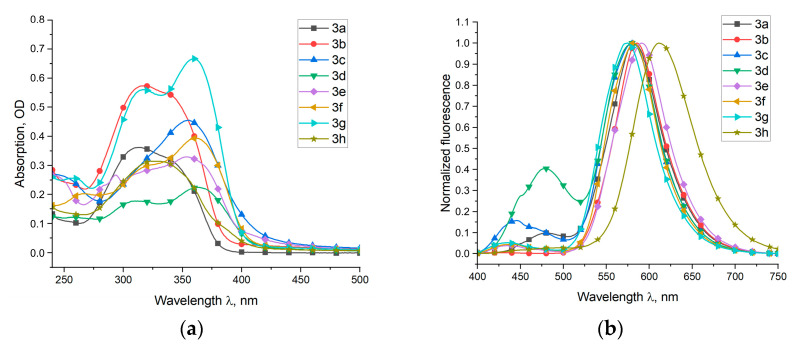
(**a**) UV–vis absorption spectra of compounds **3a**–**h** in MeOH; (**b**) fluorescence emission spectra (λ_ex_ = 380 nm) of compounds **3a**–**h** in MeOH.

The optical properties of 5-hydroxy-2,6-diphenylpyrano[2,3-*c*]pyrazol-4(2*H*)-ones **3a**–**h** in various solvents, such as polar protic (MeOH), polar aprotic (THF and DMF), and non-polar (toluene), were investigated by UV–vis spectroscopy; the compounds were also subjected to fluorimetric measurements. The UV–vis electronic absorption spectra of compounds **3a** and **3b** in MeOH showed the absorption maximum in the 337 and 341 nm, respectively (Figure 6a, Table 3, entries 1, 2). The presence of electron-donating substituents on the phenyl ring of compounds **3c**,**d** resulted in a bathochromic shift of the longest wavelength absorption transition. The presence of the 4-methoxyphenyl substituent of structure **3c** shifted λ_max_ upward by 18 nm (Table 3, entry 3), and the presence of the 3,4-dimethoxyphenyl substituent in structure **3d** shifted λ_max_ upward by 24 nm (Table 3, entry 4) compared to **3a**, respectively. The bathochromic effect of λ_max_ at 353 nm is also observed in the UV spectra of the naphthalene ring containing compound **3e**, with a significant delocalization of 10-π electrons (Table 3, entry 5). Moreover, the replacement of the phenyl ring in the molecular structure of the products by heterocyclic rings, thiophen-2-yl, furan-3-yl, and pyridin-4-yl moieties induced a significant bathochromic shift of the near-ultraviolet band compared to that of compound **3a**. Specifically, the spectra of the compounds **3f**, **3g**, and **3h** contained intense absorption bands with λ_max_ at 365, 360 and 355 nm, respectively (Table 3, entries 6, 7, 8). 

**Figure 7:**
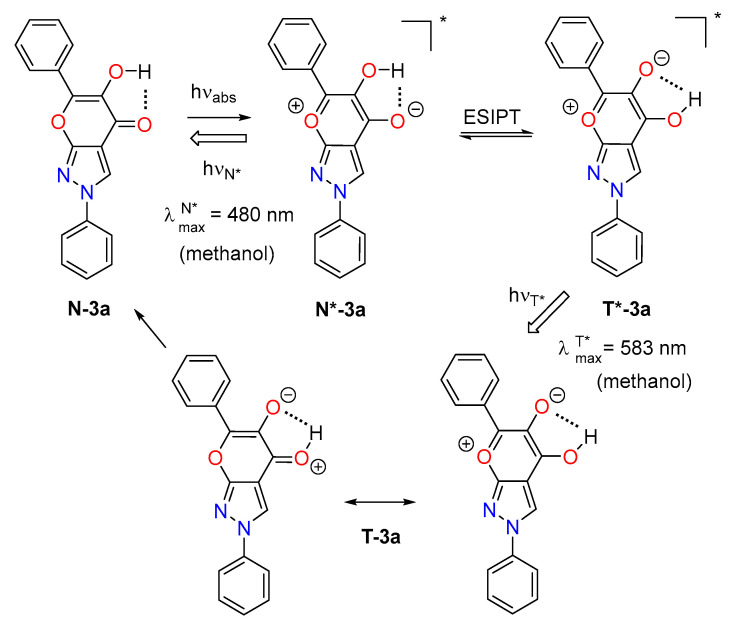
Depiction of the ESIPT process in **3a**.

The fluorescence spectra of compounds **3a**–**h** in the MeOH solution contained two well-separated fluorescence bands at around 440 and 590 nm (Figure 6b, Table 3). It is well known that the fluorescence spectra of 3-hydroxyflavone exhibit double emission due to excited-state intramolecular proton transfer (ESIPT) [67,68,69,70,71,72,73,74,75]. Similarly, in compounds **3a**–**h**, the proton transfer process (ESIPT) can occur, resulting in the formation of two forms in the excited state: the normal (N*) and tautomeric (ESIPT product, T*) forms. For example, the excitation of form **N**-**3a** leads to the excited state N*, which passes into the product T* by means of proton transfer (Figure 7). The T* form then relaxes to the ground state **T** form and emits fluorescence at a much longer wavelength compared to normal absorption [28,44]. Therefore, the form N* of the normal emission has a Stokes shift of 8927 cm^−1^, while the tautomeric product T* has a Stokes shift of 12491 cm^−1^.

Measurements of the intensity ratio of the N* and T* bands, *I*_N*_/*I*_T*_, in ESIPT compounds are used for ratiometric detection [70]. A strong effect of group substitution in the compounds **3a**–**h** was observed on the *I*_N*_/*I*_T*_ fluorescence intensity ratio. 4-Chlorophenyl-substituted compound **3b**, compared to the corresponding unsubstituted compound **3a**, showed a dramatically decreased *I*_N*_/*I*_T_ ratio of ~11-fold (Table 3, entry 2). Conversely, the 4-methoxyphenyl-substituted compound **3c** and 3,4-dimethoxyphenyl-substituted compound **3d**, compared to the corresponding compound **3a**, showed increased *I*_N*_/*I*_T*_ ratios by ~1.6- and ~4-fold, respectively (Table 3, entries 3,4). In addition, it was found that the corresponding compounds **3e**–**h**, containing the naphthalen-2-yl, thiophen-2-yl, furan-3-yl, and pyridin-4-yl groups replacing the phenyl group in compound **3a**, caused a decrease in *I*_N*_/*I*_T*_ ratios of ~2–3-fold (Table 3, entries 5–8). 

The fluorescence quantum yield (*Φ_f_*) of the solutions was estimated using the integrating sphere method. It appeared that the fluorescence quantum yield was sensitive to the structure of compounds **3a**–**h**. For unsubstituted compound **3a**, a high *Φ_f_* value was observed at 59.3%. The fluorescence quantum yield of 4-methoxyphenyl-group-containing compound **3c** was low and did not exceed 14%. The highest *Φ_f_* value (76.1%) was measured for naphthalen-2-yl-group-containing compound **3e**; the thiophen-2-yl, furan-3-yl, and pyridin-4-yl groups of compounds **3f**, **3g**, and **3h** emitted fluorescence with the observed *Φ_f_* values of 55.8%, 42.6%, and 13.1%, respectively. It is notable that 3-hydroxyflavone had low quantum yield values in methanol (*Φ*_f_ = 3%) and DMF (*Φ*_f_ = 1.3%) [70].

**Figure 8:**
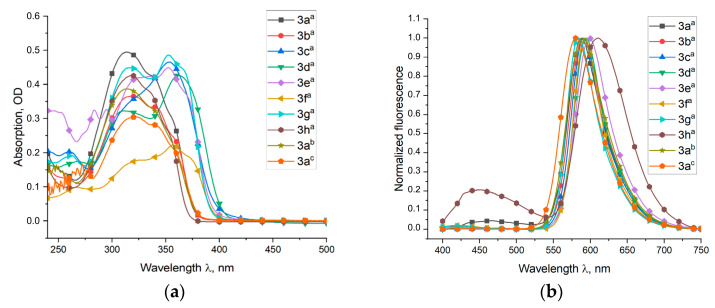
(**a**) UV–vis absorption spectra of compounds **3a**–**h** in aprotic solvents; (**b**) fluorescence emission spectra (λ_ex_ = 380 nm) of compounds **3a**–**h** in aprotic solvents.

Next, the UV–vis electronic absorption spectra of compounds **3a**–**h** in a polar aprotic solvent, THF, showed the absorption maximum in the 339–362 nm range (Figure 8a, Table 4, entries 1–8). The fluorescence spectra (*λ_ex_ = 380 nm) of compounds **3a**–**h** in the THF solution showed two fluorescence bands at around 441 nm and 591 nm (Figure 8b, Table 4, entries 1–8), which were similar to the bands in MeOH. The inhibition of the ESIPT reaction by protic solvents in 3-hydroxyflavones is associated with the formation of intermolecular H-bonds, which weaken the intramolecular H-bond necessary for the ESIPT reaction [69,74]. Therefore, the relative intensity of the N* band was very weak compared to that of the T* band for compounds **3a** in THF as an aprotic solvent. Compounds **3c**–**g**, especially the ones containing methoxyphenyl groups, presented dramatically decreased *I*_N*_/*I*_T*_ ratios in the THF solutions compared to those in MeOH, but the 4-chlorophenyl substituent possessing compound **3b** retained similar *I*_N*_/*I*_T*_ ratios. However, compound **3h** containing the pyridinyl substituent possessed reversed *I*_N*_/*I*_T*_ ratios of 0.221 from 0.031 in MeOH. It is possible that the molecule transfered the corresponding proton to pyridine instead of to the carbonyl group. In this case, the pyridin-4-yl substituent inhibits the proton transfer process (ESIPT).

The fluorescence spectra of compound **3a** in polar aprotic solvent, DMF, contained two fluorescence bands in the regions of 428 and 589 nm and showed an *I*_N*_/*I*_T*_ ratio of 0.009 (Figure 8b, Table 4, entry 9), while compound **3a** in toluene contained two fluorescence bands in the region of 430 and 589 nm and showed an *I*_N*_/*I*_T*_ ratio of 0.004 (Figure 8b, Table 4, entry 10).

Derivative **4** with the 5-MeO substituent had an electron spectrum very close to its analog **3a** (absorption maximum 306 nm and 302 nm, respectively) (Figure S1a). In the fluorescence spectrum of compound **4** in THF solution, two bands were observed at 475 and 582 nm with insignificant fluorescence (*Φ_f_* < 0.1%) (Figure S1b, Table S12, entry 1). Ormson et al. reported that the fluorescence quantum yield for 3-hydroxyflavone is much greater than for the corresponding methoxy compounds and their fluorescence lifetimes are longer [76].

The UV–vis absorption and fluorescence emission spectra of 5-substituted 2,6-diphenylpyrano[2,3-*c*]pyrazol-4(2*H*)-ones **8a**,**f**,**g** in THF were also investigated (Figure S1a, Table S12). The absorption maximum of compounds **8a**,**f**,**g** was in the range from 297 to 302 nm (near-ultraviolet region). None of the investigated compounds **8a**,**f**,**g** exhibited an absorption in the visible portion of the electronic spectrum. Upon excitation at 340 nm in THF solution, compounds **8a**,**f**,**g** showed fluorescence emission maxima (*λ*_em_) at around 593–603 nm, although fluorescence was weak (Figure S1b, Table S12). Compound **8a** derived from 2,6-diphenylpyrano[2,3-*c*]pyrazol-4(2*H*)-one containing 5-phenyl substituent produced a low quantum yield (*Φ_f_* = 1%) (Table S12, entry 2); for compounds **8f** and **8g**, the observed *Φ_f_* had a negligible value of only <0.1% (Table S12, entries 3, 4). All compounds **8a**,**f**,**g** possessed very high values of Stokes shift of ~Δυ = 16000 nm. 

**Figure 9:**
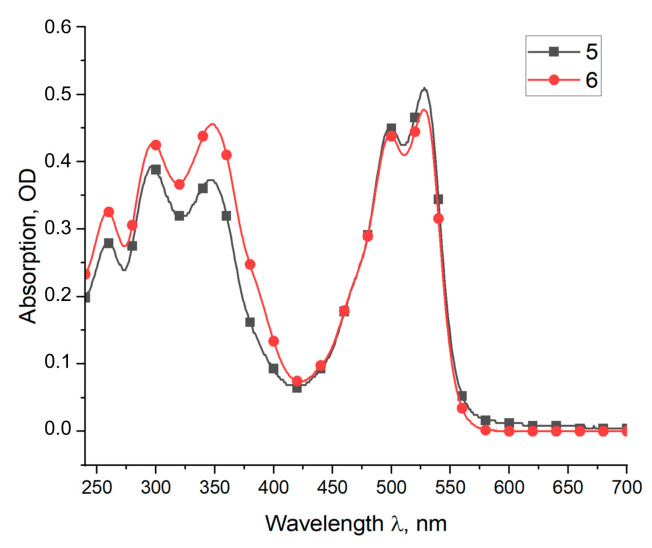
UV–vis electronic absorption spectra of compounds **5** and **6** in THF.

Finally, we investigated the UV–vis spectra of compounds **5** and **6**. In a polar aprotic solvent, THF, both compounds showed the same absorption maximum in the 528 nm (Figure 9, Table 5). The versatility of derivatives of zwitterionic chromophore, including pyran compounds, in synthetic and material applications, has been well documented [77,78,79].

## 3. Materials and Methods

### 3.1. General

All the chemicals and solvents were purchased from common commercial suppliers. Diffraction data were collected on a Rigaku, XtaLAB Synergy, Dualflex, HyPix diffractometer (Rigaku Corporation, Tokyo, Japan). The crystals were kept at 150.0(1) K while collecting the data. Using Olex2, the structure was solved with the ShelXT structure solution program using intrinsic phasing and refined with the olex2.refine refinement package using Gauss–Newton minimization. The ^1^H, ^13^C, and ^15^N NMR spectra were recorded in CDCl_3_ or DMSO-*d*_6_ at 25 °C on a Bruker Avance III 700 (700 MHz for ^1^H, 176 MHz for ^13^C, and 71 MHz for ^15^N) spectrometer (Bruker BioSpin GmbH, Rheinstetten, Germany) equipped with a 5 mm TCI ^1^H-^13^C/^15^N/D z-gradient cryoprobe. The chemical shifts were referenced to tetramethylsilane (TMS) and expressed in ppm. The ^15^N NMR spectra were referenced against neat, external nitromethane (coaxial capillary). ^19^F NMR spectrum (376 MHz) was obtained on a Bruker Avance III 400 instrument (Bruker BioSpin AG, Faellanden, Switzerland) with absolute referencing via δ ratio. The FT-IR spectra were recorded by ATR method on either a Bruker Vertex 70v spectrometer (Bruker Optik GmbH, Ettlingen, Germany) with an integrated Platinum ATR accessory or on a Bruker Tensor 27 spectrometer (Bruker Optik GmbH, Ettlingen, Germany) using KBr pellets. The melting points of the crystalline compounds were measured in open capillary tubes with a Buchi M 565 apparatus and are uncorrected. Mass spectra were obtained using a Shimadzu LCMS-2020 (ESI^+^) spectrometer (Shimadzu Corporation, Kyoto, Japan). High-resolution mass spectra (HRMS) were measured using a Bruker MicrOTOF-Q III (ESI^+^) apparatus (Bruker Daltonik GmbH, Bremen, Germany). All the reactions were performed in oven-dried glassware with magnetic stirring. The reaction progress was monitored by TLC analysis on Macherey-Nagel™ ALUGRAM^®^ Xtra SIL G/UV254 plates (Macherey-Nagel GmbH & Co. KG, Düren, Germany) which were visualized by UV light (254 and 365 nm wavelengths). The compounds were purified by flash chromatography in glass columns (stationary phase of silica gel, high-purity grade of 9385, pore size of 60 Å, and particle size of 230–400 mesh, supplied by Sigma-Aldrich; Merck KGaA, Darmstadt, Germany). The UV–vis spectra were recorded on a Shimadzu 2600 UV/vis spectrometer (Shimadzu Corporation, Japan). The fluorescence spectra were recorded on an FL920 fluorescence spectrometer from Edinburgh Instruments (Edinburgh Analytical Instruments Limited, Edinburgh, UK). The PL quantum yields were determined from dilute solutions by an absolute method using the Edinburgh Instruments integrating sphere excited with a Xe lamp. The optical densities of the sample solutions were ensured to be below 0.1 to avoid reabsorption effects. All the optical measurements were performed at room temperature under ambient conditions. The following abbreviations are used in reporting the NMR data: Ph, phenyl; Pyr, pyridine; Pz, pyrazole; Naph, naphtalene; and Th, thiophene. The ^1^H, ^13^C, and ^1^H-^15^N HMBC NMR spectra, as well as the HRMS data of the new compounds, are provided in Figures S2–S81 of the Supplementary Material. Crystallographic data have been deposited at the Cambridge Crystallographic Data Centre with CCDC reference number 2287991 for 6-(1-methylpyridin-1-ium-4-yl)-4-oxo-2-phenyl-2,4-dihydropyrano[2,3-*c*]pyrazol-5-olate (**5**); formula C_19_H_17_N_3_O_4_; unit cell parameters: a 10.39395(17) b 12.15508(19) c 13.08074(18), space group P21/c.

### 3.2. Synthetic Procedures

Compounds **2a**–**c** and **2e**–**h** were synthesized in accordance with the procedure described in ref. [44].

#### 3.2.1. (2*E*)-3-(3,4-Dimethoxyphenyl)-1-(3-hydroxy-1-phenyl-1*H*-pyrazol-4-yl)prop-2-en-1-one (**2d**)

The compound was synthesized in accordance with the procedure described in ref. [44] using 3,4-dimethoxybenzaldehyde. Orange solid. Yield 67% (2349 mg); m.p. 225–226 °C. ^1^H NMR (700 MHz, DMSO-*d*_6_): *δ*_H_ ppm 3.82 (s, 3H, 4-OCH_3_), 3.84 (s, 3H, 3-OCH_3_), 7.05 (d, *J* = 8.1 Hz, 1H, CPh 5-H), 7.33 (t, *J* = 7.4 Hz, 1H, NPh 4-H), 7.34–7.37 (m, 2H, CPh 2,6-H), 7.52 (t, *J* = 7.9 Hz, 2H, NPh 3,5-H), 7.57 (d, *J* = 15.6 Hz, 1H, C(O)CHCH), 7.66 (d, *J* = 15.6 Hz, 1H, C(O)CHCH), 7.84 (d, *J* = 7.8 Hz, 2H, NPh 2,6-H), 9.12 (s, 1H, Pz 5-H), 11.07 (s, 1H, OH). ^13^C NMR (176 MHz, DMSO-*d*_6_): *δ*_C_ ppm 55.6 (4-OCH_3_), 55.7 (3-OCH_3_), 111.0 (Pz C-4), 111.3 (CPh C-2), 111.8 (CPh C-5), 118.1 (NPh C-2,6), 121.8 (C(O)CHCH), 122.7 (CPh C-6), 126.6 (NPh C-4), 127.5 (CPh C-1), 129.6 (NPh C-3,5), 131.5 (Pz C-5), 138.9 (NPh C-1), 142.0 (C(O)CHCH), 149.0 (CPh C-3), 151.1 (CPh C-4), 162.0 (Pz C-3), 182.9 (C=O). ^15^N NMR (71 MHz, DMSO-*d*_6_): *δ*_N_ ppm −182.3 (Pz N-1), −118.3 (Pz N-2). IR (*ν*_max_, cm^−1^): 3118, 2932, 1652 (C=O), 1586, 1510, 1451, 1218, 1026, 977, 742, 679. HRMS (ESI^+^) for C_20_H_18_N_2_NaO_4_ ([M + Na]^+^) calcd 373.1159, found 373.1162.

#### 3.2.2. General Procedure for the Synthesis of **3a–h**

To a solution of **2a**–**h** (1 mmol) in EtOH (5 mL) at −10 °C, aq. NaOH (20%, 1 mL, 5 mmol) was added; the reaction mixture was cooled down to −25 °C and H_2_O_2_ 30% (0.51 mL, 5 mmol) was added dropwise. The reaction mixture was stirred for 2 h and at room temperature for another 16 h. The solids were filtered off, washed with warm water, cold MeOH, and ether, and dried. The product was recrystallized from ACN.

*5-Hydroxy-2,6-diphenylpyrano[2,3-c]pyrazol-4(2H)-one* (**3a**). Off white solid; yield 58% (177 mg); m.p. 183–184 °C. ^1^H NMR (700 MHz, DMSO-*d*_6_): *δ*_H_ ppm 7.46 (t, *J* = 7.4 Hz, 1H, NPh 4-H), 7.49 (t, *J* = 7.4 Hz, 1H, 6-CPh 4-H), 7.55–7.58 (m, 2H, 6-CPh 3,5-H), 7.58–7.61 (m, 2H, NPh 3,5-H), 8.01–8.03 (m, 2H, NPh 2,6-H), 8.13–8.14 (m, 2H, 6-CPh 2,6-H), 9.38 (s, 1H, 3-H), 9.44 (s, 1H, OH). ^13^C NMR (176 MHz, DMSO-*d*_6_): *δ*_C_ ppm 108.3 (C-3a), 119.9 (NPh C-2,6), 126.6 (C-3), 127.9 (6-CPh C-2,6), 128.5 (NPh C-4), 129.0 (6-CPh C-3,5), 130.0 (6-CPh C-4), 130.2 (NPh C-3,5), 131.8 (6-CPh C-1), 139.16 (C-5), 139.19 (NPh C-1), 144.4 (C-6), 161.2 (C-7a), 171.8 (C-4). ^15^N NMR (71 MHz, DMSO-*d*_6_): *δ*_N_ ppm −167.7 (N-2), −117.0 (N-1). IR (*ν*_max_, cm^−1^): 3110, 3062, 2920, 2850, 1679 (C=O), 1568, 1489, 1199, 1110, 913, 832, 761, 696. HRMS (ESI^+^) for C_18_H_12_N_2_NaO_3_ ([M + Na]^+^) calcd 327.0740, found 327.0740.
*6-(4-Chlorophenyl)-5-hydroxy-2-phenylpyrano[2,3-c]pyrazol-4(2H)-one* (**3b**). Light yellow solid; yield 63% (213 mg); m.p. 262–263 °C. ^1^H NMR (700 MHz, DMSO-*d*_6_): *δ*_H_ ppm 7.45 (t, *J* = 7.3 Hz, 1H, NPh 4-H), 7.58 (t, *J* = 7.8 Hz, 2H, NPh 3,5-H), 7.62 (d, *J* = 8.6 Hz, 2H, 6-CPh 3,5-H), 8.01 (d, *J* = 7.9 Hz, 2H, NPh 2,6-H), 8.15 (d, *J* = 8.6 Hz, 2H, 6-CPh 2,6-H), 9.38 (s, 1H, 3-H), 9.70 (s, 1H, OH). ^13^C NMR (176 MHz, DMSO-*d*_6_): *δ*_C_ ppm 107.9 (C-3a), 119.5 (NPh C-2,6), 126.3 (C-3), 128.1 (NPh C-4), 128.6 (6-CPh C-3,5), 129.1 (6-CPh C-2,6), 129.8 (NPh C-3,5), 130.2 (6-CPh C-1), 134.1 (6-CPh C-4), 138.7 (NPh C-1), 139.1 (C-5), 142.7 (C-6), 160.6 (C-7a), 171.3 (C-4). ^15^N NMR (71 MHz, DMSO-*d*_6_): *δ*_N_ ppm −167.5 (N-2), −117.2 (N-1). IR (*ν*_max_, cm^−1^): 3348, 3289, 3100, 1645 (C=O), 1576, 1495, 1442, 1098, 825, 752, 679. HRMS (ESI^+^) for C_18_H_11_ClN_2_NaO_3_ ([M + Na]^+^) calcd 361.0350, found 361.0350.
*5-Hydroxy-6-(4-methoxyphenyl)-2-phenylpyrano[2,3-c]pyrazol-4(2H)-one* (**3c**). Yellow solid; yield 51% (171 mg); m.p. 263–264 °C. ^1^H NMR (700 MHz, DMSO-*d*_6_): *δ*_H_ ppm 3.85 (s, 3H, CH_3_), 7.12–7.13 (m, 2H, 6-CPh 3,5-H), 7.45 (t, *J* = 7.4 Hz, 1H, NPh 4-H), 7.57–7.60 (m, 2H, NPh 3,5-H), 8.00–8.02 (m, 2H, NPh 2,6-H), 8.09–8.12 (m, 2H, 6-CPh 2,6-H), 9.27 (s, 1H, 3-H), 9.36 (s, 1H, OH). ^13^C NMR (176 MHz, DMSO-*d*_6_): *δ*_C_ ppm 55.3 (CH_3_), 107.9 (C-3a), 114.2 (6-CPh C-3,5), 119.4 (NPh C-2,6), 123.6 (6-CPh C-1), 126.0 (C-3), 128.0 (NPh C-4), 129.2 (6-CPh C-2,6), 129.8 (NPh C-3,5), 137.8 (C-5), 138.8 (NPh C-1), 144.4 (C-6), 160.2 (6-CPh C-4), 160.7 (C-7a), 171.2 (C-4). ^15^N NMR (71 MHz, DMSO-*d*_6_): *δ*_N_ ppm −168.3 (N-2). IR (*ν*_max_, cm^−1^): 3286, 3134, 1642 (C=O), 1580, 1509, 1441, 1256, 1108, 821, 748, 680. HRMS (ESI^+^) for C_19_H_14_N_2_NaO_4_ ([M + Na]^+^) calcd 357.0846, found 357.0841.
*6-(3,4-Dimethoxyphenyl)-5-hydroxy-2-phenylpyrano[2,3-c]pyrazol-4(2H)-one* (**3d**). Orange solid; yield 67% (245 mg); m.p. 252–253 °C. ^1^H NMR (700 MHz, DMSO-*d*_6_): *δ*_H_ ppm 3.84 (s, 3H, 6-CPh 3-OCH_3_), 3.85 (s, 3H, 6-CPh 4-OCH_3_), 7.15 (d, *J* = 8.7 Hz, 1H, 6-CPh 5-H), 7.45 (t, *J* = 7.4 Hz, 1H, NPh 4-H), 7.58 (t, *J* = 8.0 Hz, 2H, NPh 3,5-H), 7.71 (d, *J* = 2.1 Hz, 1H, 6-CPh 2-H), 7.78 (dd, *J* = 8.6, 2.1 Hz, 1H, 6-CPh 6-H), 8.02 (d, *J* = 7.7 Hz, 2H, NPh 2,6-H), 9.28 (s, 1H, OH), 9.35 (s, 1H, 3-H). ^13^C NMR (176 MHz, DMSO-*d*_6_): *δ*_C_ ppm 55.6 (6-CPh 3,4-OCH_3_), 107.8 (C-3a), 110.7 (6-CPh C-2), 111.5 (6-CPh C-5), 119.4 (NPh C-2,6), 121.3 (6-CPh C-6), 123.7 (6-CPh C-1), 126.0 (C-3), 128.0 (NPh C-4), 129.8 (NPh C-3,5), 137.9 (C-5), 138.8 (NPh C-1), 144.3 (C-6), 148.3 (6-CPh C-3), 150.0 (6-CPh C-4), 160.6 (C-7a), 171.1 (C-4). ^15^N NMR (71 MHz, DMSO-*d*_6_): *δ*_N_ ppm −168.2 (N-2), −117.3 (N-1). IR (*ν*_max_, cm^−1^): 3281, 2963, 1632 (C=O), 1583, 1515, 1439, 1106, 754, 657. HRMS (ESI^+^) for C_20_H_16_N_2_NaO_5_ ([M + Na]^+^) calcd 387.0951, found 387.0953.
*5-Hydroxy-6-(naphthalen-2-yl)-2-phenylpyrano[2,3-c]pyrazol-4(2H)-one* (**3e**). Yellow solid; yield 32% (113 mg); m.p. 256–257 °C. ^1^H NMR (700 MHz, DMSO-*d*_6_): *δ*_H_ ppm 7.46 (t, *J* = 7.4 Hz, 1H, NPh 4-H), 7.59–7.63 (m, 4H, NPh 3,5-H and Naph 6,7-H), 7.99 (d, *J* = 7.8 Hz, 1H, Naph 5-H), 8.04 (d, *J* = 7.9 Hz, 2H, NPh 2,6-H), 8.06–8.09 (m, 1H, Naph 4-H), 8.09–8.10 (m, 1H, Naph 8-H), 8.27 (dd, *J* = 8.7, 1.8 Hz, 1H, Naph 3-H), 8.71 (s, 1H, Naph 1-H), 9.41 (s, 1H, 3-H), 9.59 (s, 1H, OH). ^13^C NMR (176 MHz, DMSO-*d*_6_): *δ*_C_ ppm 107.8 (C-3a), 119.3 (NPh C-2,6), 124.3 (Naph C-3), 126.1 (C-3), 126.7 (Naph C-7), 127.38 (Naph C-1 and Naph C-6), 127.44 (Naph C-5), 127.82 (Naph C-4), 128.01 (NPh C-4), 128.76 (Naph C-8), 128.79 (Naph C-2), 129.7 (NPh C-3,5), 132.4 (Naph C-8a), 132.9 (Naph C-4a), 138.7 (NPh C-1), 139.0 (C-5), 143.8 (C-6), 160.7 (C-7a), 171.2 (C-4). ^15^N NMR (71 MHz, DMSO-*d*_6_): *δ*_N_ ppm −167.7 (N-2), −117.4 (N-1). IR (*ν*_max_, cm^−1^): 3240, 1629 (C=O), 1576, 1564, 1441, 1386, 1216, 1096, 753, 685. HRMS (ESI^+^) for C_22_H_14_N_2_NaO_3_ ([M + Na]^+^) calcd 377.0897, found 377.0908.
*5-Hydroxy-2-phenyl-6-(thiophen-2-yl)pyrano[2,3-c]pyrazol-4(2H)-one* (**3f**). Yellow solid; yield 62% (193 mg); m.p. 187–188 °C. ^1^H NMR (700 MHz, DMSO-*d*_6_): *δ*_H_ ppm 7.29 (dd, *J* = 5.0, 3.8 Hz, 1H, Th 5-H), 7.44–7.46 (m, 1H, NPh 4-H), 7.57–7.60 (m, 2H, NPh 3,5-H), 7.87 (dd, *J* = 3.8, 1.2 Hz, 1H, Th 3-H), 7.88 (dd, *J* = 5.0, 1.2 Hz, 1H, Th 4-H), 8.00–8.02 (m, 2H, NPh 2,6-H), 9.35 (s, 1H, 3-H), 10.12 (s, 1H, OH). ^13^C NMR (176 MHz, DMSO-*d*_6_): *δ*_C_ ppm 108.1 (C-3a), 119.3 (NPh C-2,6), 126.1 (C-3), 127.66 (Th C-3), 127.70 (Th C-5), 127.9 (NPh C-4), 129.6 (NPh C-3,5), 130.4 (Th C-4), 132.3 (Th C-2), 136.5 (C-5), 138.6 (NPh C-1), 141.9 (C-6), 160.2 (C-7a), 170.4 (C-4). ^15^N NMR (71 MHz, DMSO-*d*_6_): *δ*_N_ ppm −168.6 (N-2), −117.2 (N-1). IR (*ν*_max_, cm^−1^): 3259, 3113, 1629 (C=O), 1575, 1503, 1217, 1103, 826, 753, 685. HRMS (ESI^+^) for C_16_H_10_N_2_NaO_3_S ([M + Na]^+^) calcd 333.0304, found 333.0309.
*6-(Furan-3-yl)-5-hydroxy-2-phenylpyrano[2,3-c]pyrazol-4(2H)-one* (**3g**). Beige solid; yield 30% (89 mg); m.p. 228–229 °C. ^1^H NMR (700 MHz, DMSO-*d*_6_): *δ*_H_ ppm 6.78 (dd, *J* = 3.4, 1.7 Hz, 1H, Furanyl 5-H), 7.23 (d, *J* = 3.4 Hz, 1H, Furanyl 4-H), 7.45 (t, *J* = 7.4 Hz, 1H, NPh 4-H), 7.59 (t, *J* = 7.9 Hz, 2H, NPh 3,5-H), 8.00 (d, *J* = 7.8 Hz, 2H, NPh 2,6-H), 8.02 (d, *J* = 1.0 Hz, 1H, Furanyl 2-H), 9.36 (s, 1H, 3-H), 9.86 (s, 1H, OH). ^13^C NMR (176 MHz, DMSO-*d*_6_): *δ*_C_ ppm 108.2 (C-3a), 112.7 (Furanyl C-5), 114.5 (Furanyl C-4), 119.3 (NPh C-2,6), 126.1 (C-3), 127.9 (NPh C-4), 129.7 (NPh C-3,5), 136.9 (C-5), 138.0 (Furanyl C-3), 138.6 (NPh C-1), 144.0 (C-6), 144.9 (Furanyl C-2), 160.2 (C-7a), 170.3 (C-4). ^15^N NMR (71 MHz, DMSO-*d*_6_): *δ*_N_ ppm −168.4 (N-2), −117.1 (N-1). IR (*ν*_max_, cm^−1^): 3246, 3138, 2957, 2856, 1633 (C=O), 1576, 1483, 1221, 1124, 934, 845, 755, 681. HRMS (ESI^+^) for C_16_H_10_N_2_NaO_4_ ([M + Na]^+^) calcd 317.0533, found 317.0534.
*5-Hydroxy-2-phenyl-6-(pyridin-4-yl)pyrano[2,3-c]pyrazol-4(2H)-one* (**3h**). Yellow solid; yield 53% (163 mg); m.p. 298–299 °C. ^1^H NMR (700 MHz, DMSO-*d*_6_) δ 7.45–7.48 (m, 1H, Ph 4-H), 7.56–7.61 (m, 2H, Ph 3,5-H), 8.00–8.03 (m, 2H, Ph 2,6-H), 8.04–8.07 (m, 2H, Pyr 3,5-H), 8.74–8.77 (m, 2H, Pyr 2,4-H), 9.41 (s, 1H, 3-H), 10.13 (s, 1H, OH). ^13^C NMR (176 MHz, DMSO-*d*_6_) δ 107.8 (C-3a), 119.4 (Ph C-2,6), 120.6 (Pyr C-3,5), 126.4 (C-3), 128.1 (Ph C-4), 129.7 (Ph C-3,5), 138.45 (Pyr C-4), 138.53 (Ph C-1), 140.5 (C-6), 140.9 (C-5), 150.0 (Pyr C-2,6), 160.5 (C-7a), 171.2 (C-4). ^15^N NMR (71 MHz, DMSO-*d*_6_): *δ*_N_ ppm −166.9 (N-2), −117.1 (N-1), −62.2 (Pyr N). IR (*ν*_max_, cm^−1^): 3112, 3087, 1648 (C=O), 1571, 1500, 1442, 1228, 1026, 834, 754, 629. HRMS (ESI^+^) for C_17_H_11_N_3_O_3_ ([M + H]^+^) calcd 306.0873, found 306.0871.

#### 3.2.3. Procedure for the Synthesis of 5-Methoxy-2,6-diphenylpyrano[2,3-c]pyrazol-4(2H)-one (4)

To a solution of **3a** (304 mg, 1 mmol) in dioxane (30 mL) Cs_2_CO_3_, (0.65 g, 2 mmol) and MeI (0.07 mL, 1.1 mmol) were added. The reaction mixture was stirred at 40 °C for 3 h, neutralized with aq. KHSO_4_, and purified via column chromatography (SiO_2_, eluent: methanol/dichloromethane, 1:9, *v*/*v*). White solid; yield 79% (251 mg); m.p. 220–221 °C. ^1^H NMR (700 MHz, DMSO-*d*_6_): *δ*_H_ ppm 3.77 (s, 3H, CH_3_), 7.45 (t, *J* = 7.4 Hz, 1H, NPh 4-H), 7.56–7.61 (m, 5H, NPh 3,5-H and 6-CPh 3,4,5-H), 7.96–7.98 (m, 2H, 6-CPh 2,6-H), 8.00 (d, *J* = 8.0 Hz, 2H, NPh 2,6-H), 9.34 (s, 1H, 3-H). ^13^C NMR (176 MHz, DMSO-*d*_6_): *δ*_C_ ppm 60.1 (CH_3_), 109.8 (C-3a), 119.5 (NPh C-2,6), 126.5 (C-3), 128.1 (NPh C-4), 128.2 (6-CPh C-2,6), 128.7 (6-CPh C-3,5), 129.8 (NPh C-3,5), 130.4 (6-CPh C-1), 130.7 (6-CPh C-4), 138.7 (NPh C-1), 140.8 (C-5), 154.1 (C-6), 160.8 (C-7a), 172.1 (C-4). ^15^N NMR (71 MHz, DMSO-*d*_6_): *δ*_N_ ppm −168.0 (N-2), −115.9 (N-1). IR (*ν*_max_, cm^−1^): 3101, 2936, 1640 (C=O), 1578, 1554, 1443, 1351, 1134, 764, 753, 682. HRMS (ESI^+^) for C_19_H_14_N_2_NaO_3_ ([M + Na]^+^) calcd 341.0897, found 341.0899.

#### 3.2.4. Procedure for the Synthesis of 6-(1-Methylpyridin-1-ium-4-yl)-4-oxo-2-phenyl-2,4-dihydropyrano[2,3-*c*]pyrazol-5-olate (**5**)

To a solution of **3a** (304 mg, 1 mmol) in dioxane (30 mL), Cs_2_CO_3_ (0.65 g, 2 mmol) and MeI (0.07 mL, 1.1 mmol) were added. The reaction mixture was stirred at 40 °C for 3 h, neutralized with aq. KHSO_4_, and purified via column chromatography (SiO_2_, eluent: methanol/dichloromethane, 1:9, *v*/*v*). Red solid; yield 59% (264 mg); decomposition 240–241 °C. ^1^H NMR (700 MHz, DMSO-*d*_6_) δ 3.95 (CH_3_), 7.42 (m, 1H, Ph 4-H), 7.56 (m, 2H, Ph 3,5-H), 7.98 (m, 2H, Ph 2,6-H), 8.12–8.20 (m, 2H, Pyr 2,6-H), 9.14–9.20 (m, 2H, Pyr 3,5-H), 9.41 (s, 1H, 3-H). ^13^C NMR (176 MHz, DMSO-*d*_6_) δ 44.5 (CH_3_), 109.4 (C-3a), 112.9 (Pyr C-5), 114.6 (Pyr C-3), 119.1 (Ph C-2,6), 126.7 (C-3), 127.6 (Ph C-4), 129.6 (Ph C-3,5), 134.8 (C-6), 138.7 (Ph C-1), 140.9 (Pyr C-6), 141.6 (Pyr C-2), 143.3 (Pyr C-4), 161.2 (C-7a), 164.1 (C-5), 176.1 (C-4). ^15^N NMR (71 MHz, DMSO-*d*_6_): *δ*_N_ ppm −214.4 (Pyr N); −170.1 (N-2), −120.2 (N-1). IR (*ν*_max_, cm^−1^): 3089, 2920, 2852, 1629 (C=O), 1569, 1488, 1465, 1382, 1189, 756, 689. HRMS (ESI^+^) for C_18_H_14_N_3_O_3_ ([M + Na]^+^) calcd 342.0849, found 342.0852.

#### 3.2.5. Procedure for the Synthesis of 4-(5-Hydroxy-4-oxo-2-phenyl-2,4-dihydropyrano[2,3-*c*]pyrazol-6-yl)-1-methylpyridin-1-ium Iodide (**6**)

To a solution of **3a** (305 mg, 1 mmol) in ACN (15 mL), MeI (1 mL, 16.1 mmol) was added. The reaction mixture was stirred at 40 °C for 2 h and diluted with DMF (15 mL); the solution was slowly dripped into cold diethyl ether. The formed crystals were filtrated and washed with a small amount of MeOH and diethyl ether. Orange solid; yield 78% (348 mg); decomposition 277–278 °C. ^1^H NMR (700 MHz, DMSO-*d*_6_) δ 4.39 (CH_3_), 7.47 (m, 1H, Ph 4-H), 7.58 (m, 2H, Ph 3,5-H), 7.97 (m, 2H, Ph 2,6-H), 8.63 (m, 2H, Pyr 3,5-H), 9.03 (m, 2H, Pyr 2,6-H), 9.45 (s, 1H, 3-H), 11.49 (s, 1H, OH). ^13^C NMR (176 MHz, DMSO-*d*_6_) δ 48.0 (CH_3_), 108.4 (C-3a), 119.9 (Ph C-2,6), 123.8 (Pyr C-3,5), 127.4 (C-3), 128.9 (Ph C-4), 130.3 (Ph C-3,5), 146.1 (Pyr C-4), 138.8 (Ph C-1), 137.5 (C-6), 145.4 (C-5), 145.9 (Pyr C-2,6), 160.7 (C-7a), 171.4 (C-4). ^15^N NMR (71 MHz, DMSO-*d*_6_): *δ*_N_ ppm −183.2 (Pyr N); −165.7 (N-2), −117.1 (N-1). IR (*ν*_max_, cm^−1^): 3135, 3066, 1646 (C=O), 1575, 1497, 1441, 1388, 1232, 1197, 1109, 761. HRMS (ESI^+^) for C_18_H_14_N_3_O_3_ (M^+^) calcd 320.1030, found 320.1032.

#### 3.2.6. Procedure for the Synthesis of 4-Oxo-2,6-diphenyl-2,4-dihydropyrano[2,3-*c*]pyrazol-5-yl Trifluoromethanesulfonate (**7**)

To a solution of **3a** (304 mg, 1 mmol) in DCM (30 mL), at 0 °C, TEA (0.7 mL, 5 mmol) and Tf_2_O (0.34 mL, 2 mmol) were added dropwise. The reaction mixture was stirred at 24 °C for 16 h, diluted with DCM (100 mL), washed with brine (100 mL), and purified via column chromatography (SiO_2_, eluent: ethyl acetate/*n*-hexane, 1:6, *v*/*v*). Beige solid; yield 74% (323 mg); m.p. 200–201 °C. ^1^H NMR (700 MHz, CDCl_3_): *δ*_H_ ppm 7.46 (t, *J* = 7.3 Hz, 1H, NPh 4-H), 7.55–7.58 (m, 4H, NPh 3,5-H and 6-CPh 3,5-H), 7.62 (t, *J* = 7.3 Hz, 1H, 6-CPh 4-H), 7.78 (d, *J* = 8.0 Hz, 2H, NPh 2,6-H), 7.87 (d, *J* = 7.5 Hz, 2H, 6-CPh 2,6-H), 8.59 (s, 1H, 3-H). ^13^C NMR (176 MHz, CDCl_3_): *δ*_C_ ppm 109.4 (C-3a), 118.2 (q, ^1^*J_C,F_* = 320.8 Hz, CF_3_), 120.3 (NPh C-2,6), 125.5 (C-3), 128.4 (6-CPh C-1), 129.0 (6-CPh C-3,5), 129.1 (NPh C-4), 129.2 (6-CPh C-2,6), 130.1 (NPh C-3,5), 132.6 (6-CPh C-4), 134.6 (C-5), 138.9 (NPh C-1), 158.2 (C-6), 161.3 (C-7a), 168.8 (C-4). ^15^N NMR (71 MHz, CDCl_3_): *δ*_N_ ppm −165.8 (N-2), −112.7 (N-1). ^19^F NMR (376 MHz, CDCl_3_): *δ*_F_ ppm –74.0 (CF_3_). IR (*ν*_max_, cm^−1^): 3105, 2918, 1658 (C=O), 1594, 1553, 1425, 1208, 1134, 1019, 897, 757, 686, 600. HRMS (ESI^+^) for C_19_H_11_F_3_N_2_NaO_5_S ([M + Na]^+^) calcd 459.0233, found 459.0235.

#### 3.2.7. General Procedure for the Synthesis of 5-(Hetero)aryl-2,6-diphenylpyrano[2,3-*c*]pyrazol-4(2*H*)-ones **8a–e**

To a solution of **7** (436 mg, 1 mmol) in dioxane (15 mL), K_3_PO_4_ (634 mg, 3 mmol), KBr (131 mg, 1.1 mmol), appropriate (hetero)arylboronic acid (2.5 mmol), and Pd(PPh_3_)_4_ (69 mg, 0.06 mmol) were added. The reaction mixture was stirred at 90 °C for 16 h, diluted with H_2_O (80 mL), extracted with DCM (3 × 15 mL), and purified via column chromatography (SiO_2_, eluent: ethyl acetate/*n*-hexane, 1:6, *v*/*v*).

*2,5,6-Triphenylpyrano[2,3-c]pyrazol-4(2H)-one* (**8a**). Yellow solid; yield 95% (346 mg); m.p. 265–266 °C. ^1^H NMR (700 MHz, CDCl_3_): *δ*_H_ ppm 7.20–7.21 (m, 2H, 5-CPh 2,6-H), 7.24–7.25 (m, 2H, 6-CPh 3,5-H), 7.28–7.33 (m, 4H, 5-CPh 3,4,5-H and 6-CPh 4-H), 7.39 (d, *J* = 7.6 Hz, 2H, 6-CPh 2,6-H), 7.42 (t, *J* = 7.4 Hz, 1H, NPh 4-H), 7.53 (t, *J* = 7.9 Hz, 2H, NPh 3,5-H), 7.80 (d, *J* = 8.0 Hz, 2H, NPh 2,6-H), 8.55 (s, 1H, 3-H). ^13^C NMR (176 MHz, CDCl_3_): *δ*_C_ ppm 109.7 (C-3a), 119.9 (NPh C-2,6), 123.0 (C-5), 124.7 (C-3), 127.7 (5-CPh C-4), 128.0 (6-CPh C-3,5), 128.24 (NPh C-4), 128.29 (5-CPh C-3,5), 129.78 (6-CPh C-2,6), 129.83 (NPh C-3,5), 130.0 (6-CPh C-4), 131.4 (5-CPh C-2,6), 132.8 (5-CPh C-1), 133.0 (6-CPh C-1), 139.2 (NPh C-1), 160.8 (C-6), 162.4 (C-7a), 175.5 (C-4). ^15^N NMR (71 MHz, CDCl_3_): *δ*_N_ ppm −169.5 (N-2), −115.3 (N-1). IR (*ν*_max_, cm^−1^): 3093, 2922, 1642 (C=O), 1578, 1561, 1493, 1349, 1224, 1056, 755, 730, 694, 683. HRMS (ESI^+^) for C_24_H_16_N_2_NaO_2_ ([M + Na]^+^) calcd 387.1104, found 387.1107.
*5-(4-Methylphenyl)-2,6-diphenylpyrano[2,3-c]pyrazol-4(2H)-one* (**8b**). White solid; yield 62% (235 mg); m.p. 256–257 °C. ^1^H NMR (700 MHz, CDCl_3_): *δ*_H_ ppm 2.34 (s, 3H, CH_3_), 7.08 (m, 2H, 5-CPh 2,6-H), 7.11 (m, 2H, 5-CPh 3,5-H), 7.24–7.27 (m, 2H, 6-CPh 3,5-H), 7.32 (m, 2H, 6-CPh 4-H), 7.40–7.43 (m, 3H, 6-CPh 2,6-H, NPh 4-H), 7.53 (m, 2H, NPh 3,5-H), 7.78–7.81 (m, 2H, NPh 2,6-H), 8.55 (s, 1H, 3-H). ^13^C NMR (176 MHz, CDCl_3_): *δ*_C_ ppm 21.3 (CH_3_), 109.7 (C-3a), 119.8 (NPh C-2,6), 122.9 (C-5), 124.6 (C-3), 128.0 (6-CPh C-3,5), 128.2 (NPh C-4), 129.1 (5-CPh C-3,5), 129.6 (5-CPh C-1), 129.7 (6-CPh C-2,6), 129.8 (NPh C-3,5), 129.9 (6-Ph C-4), 131.2 (5-CPh C-2,6), 133.1 (6-CPh C-1), 137.4 (5-CPh C-4), 139.2 (NPh C-1), 160.5 (C-6), 162.3 (C-7a), 175.7 (C-4). ^15^N NMR (71 MHz, CDCl_3_): *δ*_N_ ppm −169.7 (N-2), −115.3 (N-1). IR (*ν*_max_, cm^−1^): 3098, 3023, 1649 (C=O), 1578, 1565, 1348, 1181, 1021, 755, 742, 732, 683. HRMS (ESI^+^) for C_25_H_18_N_2_O_2_ ([M + Na]^+^) calcd 401.1260, found 401.1262.
*5-(4-Methoxyphenyl)-2,6-diphenylpyrano[2,3-c]pyrazol-4(2H)-one* (**8c**). White solid; yield 77% (304 mg); m.p. 236–237 °C. ^1^H NMR (700 MHz, CDCl_3_): *δ*_H_ ppm 3.80 (s, 3H, CH_3_), 6.85 (m, 2H, 5-CPh 3,5-H), 7.12 (m, 2H, 5-CPh 2,6-H), 7.24–7.28 (m, 2H, 6-CPh 3,5-H), 7.32 (m, 2H, 6-CPh 4-H), 7.40–7.44 (m, 3H, 6-CPh 2,6-H, NPh 4-H), 7.54 (m, 2H, NPh 3,5-H), 7.80 (m, 2H, NPh 2,6-H), 8.54 (s, 1H, 3-H). ^13^C NMR (176 MHz, CDCl_3_): *δ*_C_ ppm 55.2 (CH_3_), 109.7 (C-3a), 113.9 (5-CPh C-3,5), 119.8 (NPh C-2,6), 122.5 (C-5), 124.6 (C-3), 124.8 (5-CPh C-1), 128.0 (6-CPh C-3,5), 128.2 (NPh C-4), 129.7 (6-CPh C-2,6), 129.8 (NPh C-3,5), 129.9 (6-Ph C-4), 132.5 (5-CPh C-2,6), 133.2 (6-CPh C-1), 139.2 (NPh C-1), 159.1 (5-CPh C-4), 160.5 (C-6), 162.3 (C-7a), 175.8 (C-4). ^15^N NMR (71 MHz, CDCl_3_): *δ*_N_ ppm −169.7 (N-2), −115.6 (N-1). IR (*ν*_max_, cm^−1^): 3102, 3024, 1650 (C=O), 1598, 1567, 1335, 1241, 1167, 1023, 748, 686, 549. HRMS (ESI^+^) for C_25_H_18_N_2_O_3_ ([M + Na]^+^) calcd 417.1210, found 417.1208.
*5-(4-Chlorophenyl)-2,6-diphenylpyrano[2,3-c]pyrazol-4(2H)-one* (**8d**). White solid; yield 44% (176 mg); m.p. 255–256 °C. ^1^H NMR (700 MHz, CDCl_3_): *δ*_H_ ppm 7.14 (m, 2H, 5-CPh 2,6-H), 7.27–7.31 (m, 4H, 5-CPh 3,5-H, 6-CPh 3,5-H), 7.35 (m, 2H, 6-CPh 4-H), 7.39 (m, 2H, 6-CPh 2,6-H), 7.43 (m, 1H, NPh 4-H), 7.54 (m, 2H, NPh 3,5-H), 7.80 (m, 2H, NPh 2,6-H), 8.55 (s, 1H, 3-H). ^13^C NMR (176 MHz, CDCl_3_): *δ*_C_ ppm 109.5 (C-3a), 119.9 (NPh C-2,6), 121.8 (C-5), 124.7 (C-3), 128.2 (6-CPh C-3,5), 128.4 (NPh C-4), 128.6 (5-CPh C-3,5), 129.7 (6-CPh C-2,6), 129.8 (NPh C-3,5), 130.3 (6-Ph C-4), 131.3 (5-CPh C-1), 132.7 (6-CPh C-1), 132.8 (5-CPh C-2,6), 133.7 (5-CPh C-4), 139.1 (NPh C-1), 161.0 (C-6), 162.32 (C-7a), 175.2 (C-4). ^15^N NMR (71 MHz, CDCl_3_): *δ*_N_ ppm −169.1 (N-2), −115.0 (N-1). IR (*ν*_max_, cm^−1^): 3206, 3105, 1650 (C=O), 1568, 1422, 1348, 1211, 1135, 757, 731, 686. HRMS (ESI^+^) for C_24_H_15_ClN_2_O_2_ ([M + Na]^+^) calcd 421.0714, found 421.0711.
*2,6-Diphenyl-5-(thiophen-3-yl)pyrano[2,3-c]pyrazol-4(2H)-one* (**8e**). Beige solid; yield 80% (297 mg); m.p. 265–266 °C. ^1^H NMR (700 MHz, CDCl_3_): *δ*_H_ ppm 6.88 (m, 1H, Th 4-H), 7.20 (m, 1H, Th 2-H), 7.24 (m, 1H, Th 5-H), 7.31 (m, 2H, 6-CPh 3,5-H), 7.37 (m, 2H, 6-CPh 4-H), 7.42 (m, 1H, NPh 4-H), 7.44 (m, 2H, 6-CPh 2,6-H), 7.54 (m, 2H, NPh 3,5-H), 7.80 (m, 2H, NPh 2,6-H), 8.54 (s, 1H, 3-H). ^13^C NMR (176 MHz, CDCl_3_): *δ*_C_ ppm 109.6 (C-3a), 118.1 (C-5), 119.9 (NPh C-2,6), 124.6 (C-3), 124.7 (Th C-5), 126.4 (Th C-2), 128.1 (6-CPh C-3,5), 128.3 (NPh C-4), 129.8 (Th C-4), 129.5 (6-CPh C-2,6), 129.8 (NPh C-3,5), 130.2 (6-Ph C-4), 131.9 (Th C-3), 132.2 (6-CPh C-1), 139.1 (NPh C-1), 160.8 (C-6), 162.2 (C-7a), 175.3 (C-4). ^15^N NMR (71 MHz, CDCl_3_): *δ*_N_ ppm −169.4 (N-2), −115.2 (N-1). IR (*ν*_max_, cm^−1^): 3100, 1644 (C=O), 1577, 1564, 1441, 1328, 1218, 753, 739, 685. HRMS (ESI^+^) for C_22_H_14_N_2_O_2_S ([M + H]^+^) calcd 393.0668, found 393.0669.

#### 3.2.8. Procedure for the Synthesis of *tert*-Butyl (2*E*)-3-(4-oxo-2,6-diphenyl-2,4-dihydropyrano[2,3-*c*]pyrazol-5-yl)prop-2-enoate (**8f**)

To a solution of **7** (436 mg, 1 mmol) in dry DMF (10 mL), TEA (0.28 mL, 2 mmol), *tert*-butyl acrylate (0.29 mL, 2 mmol), and Pd(PPh_3_)_2_Cl_2_ (35 mg, 0.05 mmol) were added. The reaction mixture was stirred at 100 °C for 72 h, diluted with H_2_O (100 mL), extracted with EtOAc (3 × 50 mL), washed with brine (100 mL), and purified via column chromatography (SiO_2_, eluent: dichloromethane). White solid; yield 24% (99 mg); decomposition 312 °C. ^1^H NMR (700 MHz, CDCl_3_): *δ*_H_ ppm 1.48 (s, 9H, C(CH_3_)_3_), 7.31 (d, *J* = 15.9 Hz, 1H, CHCHCOOC(CH_3_)_3_), 7.35 (d, *J* = 15.8 Hz, 1H, CHCHCOOC(CH_3_)_3_), 7.42 (t, *J* = 7.4 Hz, 1H, NPh 4-H), 7.52–7.56 (m, 5H, NPh 3,5-H and 6-CPh 3,4,5-H), 7.68 (d, *J* = 6.8 Hz, 2H, 6-CPh 2,6-H), 7.79 (d, *J* = 8.0 Hz, 2H, NPh 2,6-H), 8.54 (s, 1H, 3-H). ^13^C NMR (176 MHz, CDCl_3_): *δ*_C_ ppm 28.2 (C(CH_3_)_3_), 80.3 (C(CH_3_)_3_), 110.0 (C-3a), 115.9 (C-5), 120.0 (NPh C-2,6), 125.0 (C-5), 125.5 (CHCHCOOC(CH_3_)_3_), 128.6 (NPh C-4), 128.8 (6-CPh C-3,5), 130.0 (NPh C-3,5), 130.2 (6-CPh C-2,6), 131.6 (6-CPh C-4), 132.1 (6-CPh C-1), 135.3 (CHCHCOOC(CH_3_)_3_), 139.2 (NPh C-1), 161.7 (C-7a), 166.0 (C-6), 167.1 (CHCHCOOC(CH_3_)_3_), 175.2 (C-4). ^15^N NMR (71 MHz, CDCl_3_): *δ*_N_ ppm −168.8 (N-2), −114.4 (N-1). IR (*ν*_max_, cm^−1^): 3106, 2971, 1647 (C=O), 1584, 1554, 1445, 1290, 1149, 753, 688. HRMS (ESI^+^) for C_25_H_22_N_2_NaO_4_ ([M + Na]^+^) calcd 437.1472, found 437.1473.

#### 3.2.9. Procedure for the Synthesis of 2,6-Diphenyl-5-(phenylethynyl)pyrano[2,3-*c*]pyrazol-4(2*H*)-one (**8g**) 

To a solution of **7** (436 mg, 1 mmol) in dry DMF (10 mL), TEA (0.28 mL, 2 mmol), CuI (19 mg, 0.1 mmol), phenylacetylene (0.16 mL, 1.5 mmol), and Pd(PPh_3_)_2_Cl_2_ (42 mg, 0.06 mmol) were added. The reaction mixture was stirred at 65 °C for 1 h, diluted with H_2_O (100 mL), extracted with EtOAc (3 × 50 mL), washed with brine (100 mL), and purified via column chromatography, (SiO_2_, eluent: dichloromethane). White solid; yield 71% (276 mg); m.p. 222–223 °C. ^1^H NMR (700 MHz, CDCl_3_): *δ*_H_ ppm 7.31–7.34 (m, 3H, C≡CPh 3,4,5-H), 7.42 (t, *J* = 7.4 Hz, 1H, NPh 4-H), 7.49–7.50 (m, 2H, C≡CPh 2,6-H), 7.52–7.57 (m, 5H, NPh 3,5-H and 6-CPh 3,4,5-H), 7.79 (d, *J* = 8.1 Hz, 2H, NPh 2,6-H), 8.25 (d, *J* = 7.9 Hz, 2H, 6-CPh 2,6-H), 8.55 (s, 1H, 3-H). ^13^C NMR (176 MHz, CDCl_3_): *δ*_C_ ppm 82.0 (C≡CPh), 97.9 (C≡CPh), 107.7 (C-5), 108.8 (C-3a), 120.0 (NPh C-2,6), 123.3 (C≡CPh C-1), 124.8 (C-3), 128.3 (6-CPh C-3,5), 128.4 (C≡CPh C-3,5), 128.6 (C≡CPh C-4 and NPh C-4), 129.4 (6-CPh C-2,6), 130.0 (NPh C-3,5), 131.66 (6-CPh C-4), 131.71 (C≡CPh C-2,6), 132.3 (6-CPh C-1), 139.1 (NPh C-1), 161.9 (C-7a), 165.1 (C-6), 174.2 (C-4). ^15^N NMR (71 MHz, CDCl_3_): *δ*_N_ ppm −168.6 (N-2), −114.3 (N-1). IR (*ν*_max_, cm^−1^): 3094, 3057, 1649 (C=O), 1577, 1542, 1442, 1361, 1264, 1118, 750, 684. HRMS (ESI^+^) for C_26_H_16_N_2_NaO_2_ ([M + Na]^+^) calcd 411.1104, found 411.1101.

## 4. Conclusions

In conclusion, we showed that the diverse 6-aryl-5-hydroxy-2-phenylpyrano[2,3-*c*]pyrazol-4(2*H*)-one derivatives as analogues of 3-hydroxyflavones can be conveniently synthesized from appropriate (*E*)-1-(3-hydroxy-1-phenyl-1*H*-pyrazol-4-yl)prop-2-en-1-ones employing Algar–Flynn–Oyamada reaction conditions. Further functionalization of the 5-position of the pyrano[2,3-*c*]pyrazol-4(2*H*)-one ring was achieved by employing various Pd-catalyzed coupling reactions of the intermediate 5-triflate. Extensive NMR spectroscopic studies were undertaken using standard and advanced methods to unambiguously determine the structure and configuration of the synthesized compounds. The synthesized 3-hydroxyflavone analogues were characterized by good quantum yields and large Stokes shifts. In addition, the excited-state intramolecular proton transfer (ESIPT) reaction of 5-hydroxypyrano[2,3-*c*]pyrazol-4(2*H*)-one from the 5-hydroxy moiety to the carbonyl group in polar protic, polar aprotic, and non-polar solvents was observed, resulting in a well-resolved two-band fluorescence.

## Figures and Tables

**Table 1:** Selected bond lengths [Å] for compound **5**.

Atom	Atom	Length/Å	Atom	Atom	Length/Å
N1	N2	1.3839(12)	C4	C5	1.5140(14)
N1	C7A	1.3233(14)	C4	O14	1.2265(14)
N2	C3	1.3458(14)	C5	C6	1.4047(15)
N2	C8	1.4287(13)	C5	O15	1.2723(13)
C3	C3A	1.3842(14)	C6	O7	1.4135(12)
C3A	C4	1.4411(14)	C6	C16	1.4308(14)
C3A	C7A	1.4017(14)	O7	C7A	1.3387(12)

**Table 2:** Selected bond angles [°] for compound **5**.

Atom	Atom	Atom	Angle/°	Atom	Atom	Atom	Angle/°
C7A	N1	N2	102.23(8)	O14	C4	C5	120.99(10)
C3	N2	N1	113.26(8)	C6	C5	C4	119.08(9)
C8	N2	N1	119.35(8)	O15	C5	C4	116.91(9)
C8	N2	C3	127.39(9)	O15	C5	C6	124.01(10)
C3A	C3	N2	106.46(9)	O7	C6	C5	123.66(9)
C4	C3A	C3	134.37(10)	C16	C6	C5	125.60(10)
C7A	C3A	C3	104.07(9)	C16	C6	O7	110.74(9)
C7A	C3A	C4	121.56(9)	C7A	O7	C6	116.70(8)
C5	C4	C3A	113.86(9)	C3A	C7A	N1	113.97(9)
O14	C4	C3A	125.15(10)	O7	C7A	N1	120.98(9)

**Table 3:** Absorption (λ_abs_ of the absorption maxima and ε), fluorescence emission (*λ*^N*^_em_, λ^T*^_em_, ratio *I*_N*_/*I*_T*_ and quantum yield *Φ_f_*) parameters, and Stokes shifts for **3a**–**h** in MeOH (^∗^λ_ex_ = 380 nm); sh = shoulder.

Entry	Comp.	*λ*_abs_ (nm)	ε × 10^3^ (dm^3^ mol^−1^ cm^−1^)	*λ*^N*^_em_ (nm)	*λ*^T*^_em_ (nm)	*I*_N*_/*I*_T*_	Stokes Shift (cm^−1^)	*Φ_f_* (%)
**1**	**3a**	337sh 311	70.89 78.45	482	582	0.102	8927 12491	59.3
**2**	**3b**	341sh 317	112.50 119.51	428	586	0.009	5961 12261	42.7
**3**	**3c**	355 320sh 240	93.74 66.27 54.91	446	582	0.161	5747 10987	13.4
**4**	**3d**	361 311 261	43.82 34.60 23.65	479	580	0.406	6824 10459	52.7
**5**	**3e**	353 321sh 310sh 293 245sh	70.76 60.39 57.42 56.73 55.59	435	591	0.043	5340 11408	76.1
**6**	**3f**	365 317sh 266	80.34 59.96 41.23	438	582	0.046	4566 10215	55.8
**7**	**3g**	360 317 260	138.88 116.75 53.16	435	575	0.054	4789 10386	42.6
**8**	**3h**	355sh 329	49.99 61.54	493	611	0.031	7885 11802	13.1

**Table 4:** Absorption (*λ*_abs_ absorption maxima and ε), fluorescence emission (*λ*^N*^_em_, λ^T*^_em_, ratio *I*_N*_/*I*_T*_, and quantum yield *Φ_f_*) parameters, and Stokes shifts for **3a**–**h** in aprotic solvents (^a^ THF, ^b^ DMF, and ^c^ toluene) (^*^λ_ex_ = 380 nm); sh = shoulder.

Entry	Comp.	*λ*_abs_ (nm)	ε × 10^3^ (dm^3^ mol^−1^ cm^−1^)	*λ*^N*^_em_ (nm)	*λ*^T*^_em_ (nm)	*I*_N*_/*I*_T*_	Stokes Shift (cm^−1^)	*Φ_f_* (%)
**1**	**3a** ^a^	339sh 315	58.67 69.15	466	588	0.014	8039 12492	59.2
**2**	**3b** ^a^	339 317 240	49.24 53.74 22.77	428	590	0.019	6134 6134	75.5
**3**	**3c** ^a^	353 318sh 260	69.71 52.84 30.30	428	591	0.005	4964 11408	19.2
**4**	**3d** ^a^	362 311 268	58.61 44.19 23.99	429	594	0.006	4314 10789	39.6
**5**	**3e** ^a^	352 334sh 295 283	63.18 59.61 46.02 45.73	416	598	0.012	4371 11687	50.6
**6**	**3f** ^a^	357 327sh 266	35.90 28.75 15.87	423	593	0.023	43,701 11148	41.2
**7**	**3g** ^a^	352 317 262	82.58 75.99 32.57	421	584	0.020	4656 11286	55.0
**8**	**3h** ^a^	351sh 335sh 319	40.75 57.39 64.62	442	610	0.221	5866 12097	30.1
**9**	**3a** ^b^	338sh 315	64.22 75.82	428	589	0.009	6221 12608	45.5
**10**	**3a** ^c^	357sh 338 322	43.31 57.48 62.27	430	581	0.004	4755 10800	67.7

**Table 5:** Absorption (*λ*_abs_ absorption maxima and ε) of compounds **5** and **6** in THF.

Entry	Comp.	*λ*_abs_ (nm)	ε × 10^3^ (dm^3^ mol^−1^ cm^−1^)
**1**	**5**	528	0.55
		499	0.48
		346	0.39
		299	0.42
		261	0.30
**2**	**6**	528	0.54
		499	0.49
		348	0.52
		297	0.49
		261	0.37

## Data Availability

The data that support the findings of this study are available from the corresponding author upon reasonable request.

## Supplementary Materials

The following supporting information can be downloaded at: https://www.mdpi.com/article/10.3390/molecules28186599/s1. Table S1: Optimization of AFO reaction conditions of (*E*)-1-(3-hydroxy-1-phenyl-1*H*-pyrazol-4-yl)-3-phenylprop-2-en-1-one (**2a**) to get 5-hydroxy-2,6-diphenylpyrano[2,3-*c*]pyrazol-4(2*H*)-one (**3a**); Table S2: Relevant ^1^H and ^13^C NMR spectral data of 6-(hetero)aryl-5-hydroxy-2-phenylpyrano[2,3-*c*]pyrazol-4(2*H*)-ones **3a**–**h** in DMSO-*d*_6_ (δ in ppm); Tables S3–S11: Data for X-ray analysis of compound **5**: Table S3: Experimental parameters and CCDC-2287991; Table S4: Sample and crystal data of compound **5**; Table S5: Data collection and structure refinement of compound **5**; Table S6: Methanol formed and other selected hydrogen bonds in monocrystal of compound **5**; Table S7: Fractional Atomic Coordinates (×10^4^) and Equivalent Isotropic Displacement Parameters (Å^2^×10^3^) for compound **5**; Table S8: Anisotropic displacement parameters (Å^2^×10^3^) for compound **5**; Table S9: Bond lengths for compound **5**; Table S10: Bond angles for compound **5**; Table S11: Hydrogen atom coordinates (Å×10^4^) and isotropic displacement parameters (Å^2^×10^3^) for compound **5**; Figure S1: (a) UV absorption spectra of compounds **4** and **8a**,**f**,**g** in THF; (b) fluorescence emission spectra (λ_ex_ = 340 nm) of compounds **4** and **8a**,**f**,**g** in THF; Table S12**:** Absorption (*λ*_abs_ absorption maxima and ε), fluorescence emission (*λ*_em_ and quantum yield Φ*_f_*), and Stokes shift parameters for **4** and **8a**,f,g in THF (^∗^*λ*_ex_ = 340 nm); Figures S2–S81: ^1^H, ^13^C, ^1^H-^15^N HMBC NMR, and HRMS (ESI) spectra of compounds **2d**, **3a**–**h**, **4–7**, **8a**–**g**. References [80,81,82] are cited in the Supplementary Materials.

## Sample Availability

Not available.
