# Supplementary material for: Synthesis and Characterization of New Pyrano[2,3-c]pyrazole Derivatives as 3-Hydroxyflavone Analogues
Source: Molecules. 2023 Sep 13;28(18):6599. doi: 10.3390/molecules28186599 (PMC10537540; doi:10.3390/molecules28186599)
Supplement: Supplementary file 1 [file molecules-28-06599-s001.zip › molecules-2528522-supplementary.pdf]

Supplementary Material

Synthesis and Characterization of New Pyrano[2,3-*c*]pyrazole Derivatives as 3-Hydroxyflavone Analogues

Arminas Urbonavičius [1,2], Sonata Krikštolaitytė [1], Aurimas Bieliauskas [2], Vytas Martynaitis [1], Joana Solovjova [1], Asta Žukauskaitė [1,3], Eglė Arbačiauskienė [1,*] and Algirdas Šačkus [2,*]

1 Department of Organic Chemistry, Kaunas University of Technology, Radvilėnų pl. 19,

LT-50254 Kaunas, Lithuania; arminas.urbonavicius@ktu.lt (A.U.); sonata.krikstolaityte@ktu.lt (S.K.);

vytas.martynaitis@ktu.lt (V.M.); joana.solovjova@ktu.lt (J.S.); asta.zukauskaite@upol.cz (A.Ž.)

2 Institute of Synthetic Chemistry, Kaunas University of Technology, K. Baršausko g. 59,

LT-51423 Kaunas, Lithuania; aurimas.bieliauskas@ktu.lt

3 Department of Chemical Biology, Palacký University, Šlechtitelů 27, CZ-78371 Olomouc, Czech Republic

* Correspondence: egle.arbaciauskiene@ktu.lt (E.A.); algirdas.sackus@ktu.lt (A.Š.)

Table S1. Optimization of AFO reaction conditions of (*E*)-1-(3-hydroxy-1-phenyl-1*H*-pyrazol-4-yl)-3-phenylprop-2-en-1-one (**2a**) to get 5-hydroxy-2,6-diphenylpyrano[2,3-*c*]pyrazol-4(2*H*)-one (**3a**)

Entry	Base	Solvent, v/v ratio	H_2O_2 (eq)	Yield (%)
1	NaOAc	EtOH/H_2O, 2:1	5	0%
2	$NaHCO_3$	EtOH/H_2O, 2:1	5	0%
3	Na_2CO_3	EtOH/H_2O, 2:1	5	0%
4	TEA	EtOH	5	0%
5	NaOH	EtOH/H_2O, 2:1	5	24%
**6**	**NaOH**	**EtOH**	**5**	**58%**
7	NaOH	EtOH	2.5	49%
8	NaOH	EtOH	10	19%
9	KOH	EtOH	5	17%

Table S2. Relevant ^{1}H and ^{13}C NMR spectral data of 6-(hetero)aryl-5-hydroxy-2-phenylpyrano[2,3-*c*]pyrazol-4(2*H*)-ones **3a**–**h** in DMSO-d_6 (δ in ppm).

	**3a**		**3b**		**3c**		**3d**		**3e**		**3f**		**3g**		**3h**	
**Position**	δ_H	δ_C	δ_H	δ_C	δ_H	δ_C	δ_H	δ_C	δ_H	δ_C	δ_H	δ_C	δ_H	δ_C	δ_H	δ_C
3	9.38	126.6	9.38	126.3	9.36	126.0	9.35	126.0	9.41	126.1	9.35	126.1	9.36	126.1	9.42	126.4
3a	-	108.3	-	107.9	-	107.9	-	107.8	-	107.8	-	108.1	-	108.2	-	107.8
4	-	171.8	-	171.3	-	171.2	-	171.1	-	171.3	-	170.4	-	170.3	-	171.2
5	-	139.16	-	139.1	-	137.8	-	137.9	-	139.0	-	136.5	-	136.9	-	140.9
6	-	144.4	-	142.7	-	144.4	-	144.3	-	143.8	-	141.9	-	144.0	-	140.5
7a	-	161.2	-	160.6	-	160.7	-	160.6	-	160.7	-	160.2	-	160.2	-	160.5
5-OH	9.44	-	9.70	-	9.27	-	9.28	-	9.59	-	10.12	-	9.86	-	10.13	-

X-ray analysis of compound 5**:**

Single crystals of $C_{19}H_{17}N_3O_4$ 5 were investigated on a Rigaku, XtaLAB Synergy, Dualflex, HyPix diffractometer. The crystal was kept at 150.0(2) K during data collection. Using Olex2 [1], the structure was solved with the ShelXT [2] structure solution program using Intrinsic Phasing and refined with the olex2.refine [3] refinement package using Gauss-Newton minimisation.

Table S3. Experimental parameters and CCDC-2287991

Sample	Machine	Source	Temp.	Detector distance	Time/Frame	#Frames	Frame width	CCDC
			[K]	[mm]	[s]		[°]	
**5**	Rigaku, XtaLAB Synergy, Dualflex, HyPix	micro-focus sealed X-ray tube, PhotonJet (Cu)	150.0(2)	34.0	1.54	3190	0.50	2287991

Table S4. Sample and crystal data of compound **5**.

**Chemical formula**	$C_{19}H_{17}N_3O_4$	**Crystal system**	Monoclinic	
**Formula weight [g/mol]**	351.36	**Space group**	$P2_1/c$	
**Temperature [K]**	150	**Z**	4	
**Measurement method**	ϕ and ω scans	**Volume [Å^3]**	1638.14(4)	
**Radiation vawelength [Å]**	1.54184	**Unit cell dimensions [Å^3] and [°]**	10.3940(17)<br>12.1551(19)<br>13.0807(18)	90<br>97.5883(14)<br>90
**Crystal size/ [mm^3]**	0.13 × 0.09 × 0.02			
**Crystal habit**	Red prism			
**Density (calculated)/ [g/cm^3]**	1.4246	**Absorption coefficient [mm^{-1}]**	0.843	
**Abs. Correction T_{min}**		**Abs. Correction T_{max}**		
**Abs. Correction type**		**F(000) [e$^-$]**	736	

Table S5. Data collection and structure refinement of compound **5**.

**Index ranges**	$-13 \leq h \leq 13$<br>$-15 \leq k \leq 13$<br>$-13 \leq l \leq 16$	**2θ range for data collection [°]**	160.0	
**Reflections numbers**	18063	**Data / restraints / parameters**	3569/0/249	
**Refinement method**	Least squares matrix: full	**Final R indices**	All data	R_1 = 0.0399<br>wR_2 = 0.1097
**Function minimized**	$\Sigma w[\lvert Fo\rvert^2 - (1/k)\lvert Fc\rvert^2]$		I > 2σ(I)	R_1 = 0.0382<br>wR_2 = 0.1080
**Goodness-of-fit on F2**	1.045	**Weighting scheme**	$w = 1/[\sigma^2(F_o^2) + (0.0635P)^2 0.5590P]$ where $P = (F_o^2 + 2F_c^2)/3$	
**Largest diff. peak and hole [e Å^{-3}]**	+0.34/-0.23	**Flack's *x* parameter**		

Table S6. Methanol formed and other selected hydrogen bonds in monocrystal of compound **5**.

Bond	Length (Å)		Angle/°
	X-H	H···A	X-H···A
C(1m)-H(1mc)···N(2)	0.98	2.670	150.08
C(1m)-H(1ma)···O(7)	0981	2.696	131.28
O(1m)-H(1m)···O15	0.822	1.917	171.87
C(18)-H(18)···O(1m)	0.95	2.294	161.73
C(22)-H(22a)···O(1m)	0.98	2.759	133.24
C(22)-H(22b)···O(15)	0.98	2.483	172.27
C(20)-H(20)···N(1)	0.95	2.859	135.83
C(3)-H(3)···O(15)	0.95	2.417	137.47
C(17)-H(17)···O(15)	0.927	2.207	123.89

Table S7. Fractional atomic coordinates (×10^4) and equivalent isotropic displacement parameters (Å^2×10^3) for compound **5**. U_{eq} is defined as 1/3 of of the trace of the orthogonalised U_{IJ} tensor.

Atom	*X*	*Y*	*z*	*U*(eq)
N1	1681.7(9)	5763.5(8)	5121.7(7)	20.0(2)
N2	1451.4(8)	5072.8(7)	4276.3(7)	18.0(2)
C3	1583.6(10)	4000.1(9)	4522.8(8)	19.2(2)
C3A	1911.7(10)	3953.5(8)	5582.5(8)	18.2(2)
C4	2166.6(10)	3073.0(9)	6319.2(8)	19.9(2)
C5	2540.8(10)	3461.6(9)	7417.8(8)	19.2(2)
C6	2575.4(10)	4596.0(9)	7624.7(8)	19.1(2)
O7	2250.5(8)	5410.6(6)	6863.2(6)	21.49(19)
C7A	1955.4(10)	5058.7(9)	5890.7(8)	18.2(2)
C8	1120.7(10)	5536.8(9)	3272.6(8)	18.7(2)
C9	836.7(10)	6652.9(9)	3174.4(8)	21.3(2)
C10	522.1(11)	7098.8(10)	2193.0(9)	24.5(2)
C11	446.4(11)	6435.2(10)	1321.9(9)	26.3(3)
C12	730.4(11)	5322.9(10)	1435.3(8)	25.3(2)
C13	1093.3(10)	4868.2(9)	2405.5(8)	21.9(2)
O14	2092.8(9)	2089.0(7)	6111.9(6)	30.1(2)
O15	2819.0(8)	2723.3(6)	8100.8(6)	23.54(19)
C16	2931.9(10)	5088.6(9)	8615.0(8)	19.9(2)

C17	3183.3(11)	4472.5(9)	9537.8(8)	22.3(2)
C18	3500.5(11)	4993.3(10)	10467.6(8)	23.0(2)
N19	3578.3(9)	6100.0(8)	10535.2(7)	22.5(2)
C20	3346.5(12)	6719.5(10)	9668.6(9)	27.6(3)
C21	3038.0(12)	6246.6(10)	8723.0(9)	26.8(3)
C22	3912.5(12)	6633.8(11)	11546.9(9)	29.1(3)
C1M	4650.1(13)	504.6(12)	8750.7(10)	36.1(3)
O1M	4300.4(10)	936.6(9)	7759.9(7)	37.6(2)

Table S8. Anisotropic displacement parameters (Å^2×10^3) for compound **5**. The anisotropic displacement factor exponent takes the form: $-2\pi^2[h^2a^{*2}U_{11}+2hka^*b^*U_{12}+\ldots]$.

**Atom**	U_{11}	U_{22}	U_{33}	U_{12}	U_{13}	U_{23}
N1	26.0(5)	16.6(4)	16.9(4)	0.8(3)	0.9(3)	-1.2(3)
N2	21.2(4)	17.0(4)	15.4(4)	0.1(3)	1.1(3)	-0.6(3)
C3	22.3(5)	16.2(5)	18.7(5)	-1.9(4)	1.5(4)	0.1(4)
C3A	20.8(5)	16.2(5)	17.7(5)	-0.8(4)	2.6(4)	-0.4(4)
C4	24.3(5)	16.5(5)	19.1(5)	0.1(4)	3.8(4)	0.5(4)
C5	21.9(5)	18.4(5)	17.6(5)	0.1(4)	3.3(4)	1.2(4)
C6	23.5(5)	17.3(5)	16.4(5)	1.8(4)	2.5(4)	2.8(4)
O7	33.3(4)	15.0(4)	15.5(4)	2.5(3)	0.6(3)	0.5(3)
C7A	20.2(5)	17.8(5)	16.3(5)	0.8(4)	2.1(4)	-0.5(4)
C8	17.0(5)	21.2(5)	17.6(5)	-0.7(4)	1.2(4)	2.4(4)
C9	22.4(5)	20.9(5)	20.3(5)	1.1(4)	1.4(4)	0.1(4)
C10	25.7(5)	22.3(5)	25.0(5)	3.9(4)	1.2(4)	5.4(4)
C11	27.5(6)	31.6(6)	19.2(5)	2.9(5)	0.4(4)	6.2(4)
C12	28.2(5)	29.2(6)	18.3(5)	-0.6(5)	2.0(4)	-1.1(4)
C13	24.5(5)	20.5(5)	20.7(5)	0.3(4)	2.5(4)	0.4(4)
O14	51.7(5)	16.0(4)	22.3(4)	-0.8(4)	3.7(4)	-0.8(3)
O15	34.2(4)	17.1(4)	18.9(4)	1.9(3)	2.1(3)	3.2(3)

C16	21.8(5)	19.5(5)	18.5(5)	1.4(4)	3.2(4)	-0.4(4)
C17	27.9(6)	20.5(5)	18.5(5)	-0.6(4)	3.4(4)	-0.1(4)
C18	27.6(5)	23.1(5)	18.4(5)	-0.2(4)	3.1(4)	1.4(4)
N19	24.7(5)	24.0(5)	18.6(4)	-0.8(4)	1.8(3)	-3.5(4)
C20	38.9(6)	19.2(5)	23.7(5)	1.5(5)	0.3(5)	-1.2(4)
C21	38.2(6)	20.6(5)	20.7(5)	2.7(5)	0.5(4)	0.4(4)
C22	33.8(6)	32.0(6)	20.6(5)	-2.9(5)	0.8(4)	-7.7(5)
C1M	30.7(6)	44.1(8)	32.7(6)	4.0(5)	1.1(5)	6.7(6)
O1M	48.2(6)	36.1(5)	26.5(5)	15.3(4)	-2.5(4)	-3.0(4)

Table S9. Bond lengths for compound **5**.

Atom	Atom	Length/Å	Atom	Atom	Length/Å
N1	N2	1.3839(12)	C8	C9	1.3907(15)
N1	C7A	1.3233(14)	C8	C13	1.3925(15)
N2	C3	1.3458(14)	C9	C10	1.3920(15)
N2	C8	1.4287(13)	C10	C11	1.3897(16)
C3	C3A	1.3842(14)	C11	C12	1.3877(17)
C3A	C4	1.4411(14)	C12	C13	1.3900(15)
C3A	C7A	1.4017(14)	C16	C17	1.4152(15)
C4	C5	1.5140(14)	C16	C21	1.4174(16)
C4	O14	1.2265(14)	C17	C18	1.3721(15)
C5	C6	1.4047(15)	C18	N19	1.3498(15)
C5	O15	1.2723(13)	N19	C20	1.3557(15)
C6	O7	1.4135(12)	N19	C22	1.4737(14)
C6	C16	1.4308(14)	C20	C21	1.3633(16)
O7	C7A	1.3387(12)	C1M	O1M	1.4010(16)

Table S10. Bond angles for compound **5**.

Atom	Atom	Atom	Angle/°	Atom	Atom	Atom	Angle/°
C7A	N1	N2	102.23(8)	O7	C7A	C3A	125.04(9)
C3	N2	N1	113.26(8)	C9	C8	N2	119.50(9)
C8	N2	N1	119.35(8)	C13	C8	N2	119.65(10)
C8	N2	C3	127.39(9)	C13	C8	C9	120.85(10)
C3A	C3	N2	106.46(9)	C10	C9	C8	119.09(10)
C4	C3A	C3	134.37(10)	C11	C10	C9	120.73(11)
C7A	C3A	C3	104.07(9)	C12	C11	C10	119.32(10)
C7A	C3A	C4	121.56(9)	C13	C12	C11	120.87(10)
C5	C4	C3A	113.86(9)	C12	C13	C8	119.05(10)
O14	C4	C3A	125.15(10)	C17	C16	C6	123.19(10)
O14	C4	C5	120.99(10)	C21	C16	C6	120.88(10)
C6	C5	C4	119.08(9)	C21	C16	C17	115.93(10)
O15	C5	C4	116.91(9)	C18	C17	C16	120.50(10)
O15	C5	C6	124.01(10)	N19	C18	C17	121.54(10)
O7	C6	C5	123.66(9)	C20	N19	C18	119.75(9)
C16	C6	C5	125.60(10)	C22	N19	C18	120.18(10)
C16	C6	O7	110.74(9)	C22	N19	C20	120.07(10)
C7A	O7	C6	116.70(8)	C21	C20	N19	121.26(11)
C3A	C7A	N1	113.97(9)	C20	C21	C16	121.00(11)
O7	C7A	N1	120.98(9)				

Table S11. Hydrogen atom coordinates (Å×10^4) and isotropic displacement parameters (Å^2×10^3) for compound **5**.

Atom	*x*	*Y*	*z*	*U*(iso)
H3	1473.5(10)	3394.3(9)	4060.6(8)	23.0(3)
H9	857.2(10)	7104.8(9)	3768.8(8)	25.6(3)
H10	357.4(11)	7865.3(10)	2117.8(9)	29.4(3)

H11	202.7(11)	6740.0(10)	655.7(9)	31.6(3)
H12	676.0(11)	4866.0(10)	841.9(8)	30.4(3)
H13	1319.4(10)	4112.2(9)	2476.1(8)	26.3(3)
H17	3104(14)	3713(14)	9527(12)	28(4)
H18	3669.3(11)	4565.0(10)	11078.0(8)	27.6(3)
H20	3400.2(12)	7498.1(10)	9721.4(9)	33.1(3)
H21	2856(16)	6701(15)	8115(13)	40(4)
H22a	3637(9)	6164(4)	12086.5(12)	43.6(4)
H22b	4853.2(17)	6747(8)	11680(4)	43.6(4)
H22c	3470(8)	7346(4)	11547(3)	43.6(4)
H1Ma	5414(7)	31(8)	8752(2)	54.1(5)
H1Mb	4854(11)	1108.3(13)	9242.1(18)	54.1(5)
H1Mc	3928(4)	72(8)	8950(4)	54.1(5)
H1M	3818(19)	1465(18)	7804(14)	50(5)

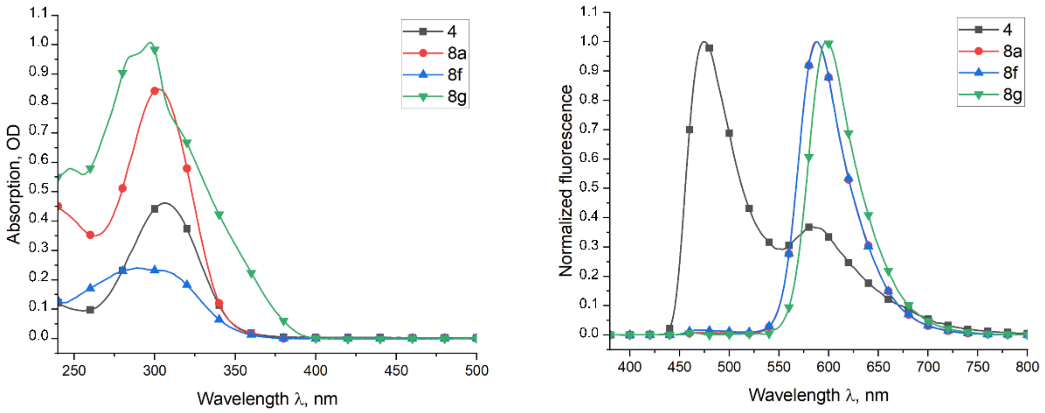

(**a**) (**b**)

Figure S1. (**a**) UV absorption spectra of compounds **4** and **8a**,**f**,**g** in THF; (**b**) fluorescence emission spectra (λ_{ex} = 340 nm) of compounds **4** and **8a**,**f**,**g** in THF.

Table S12. Absorption (λ_{abs} absorption maxima and ε), fluorescence emission (λ_{em} and quantum yield Φ_f), and Stokes shift parameters for **4** and **8a**,**f**,**g** in THF (*λ_{ex} = 340 nm).

Entry	Comp.	λ_{abs} (nm)	ε×10^3 (dm^3 mol^{-1} cm^{-1})	λ_{em} (nm)	Stokes shift (cm^{-1})	Φ_f (%)
**1**	**4**	306	62.75	475	11627	<0.1
				582	15498	
**2**	**8a**	302	110.31	593	16249	1
**3**	**8f**	302sh	28.02	593	16249	<0.1
**4**	**8g**	297	118.62	603	17086	<0.1

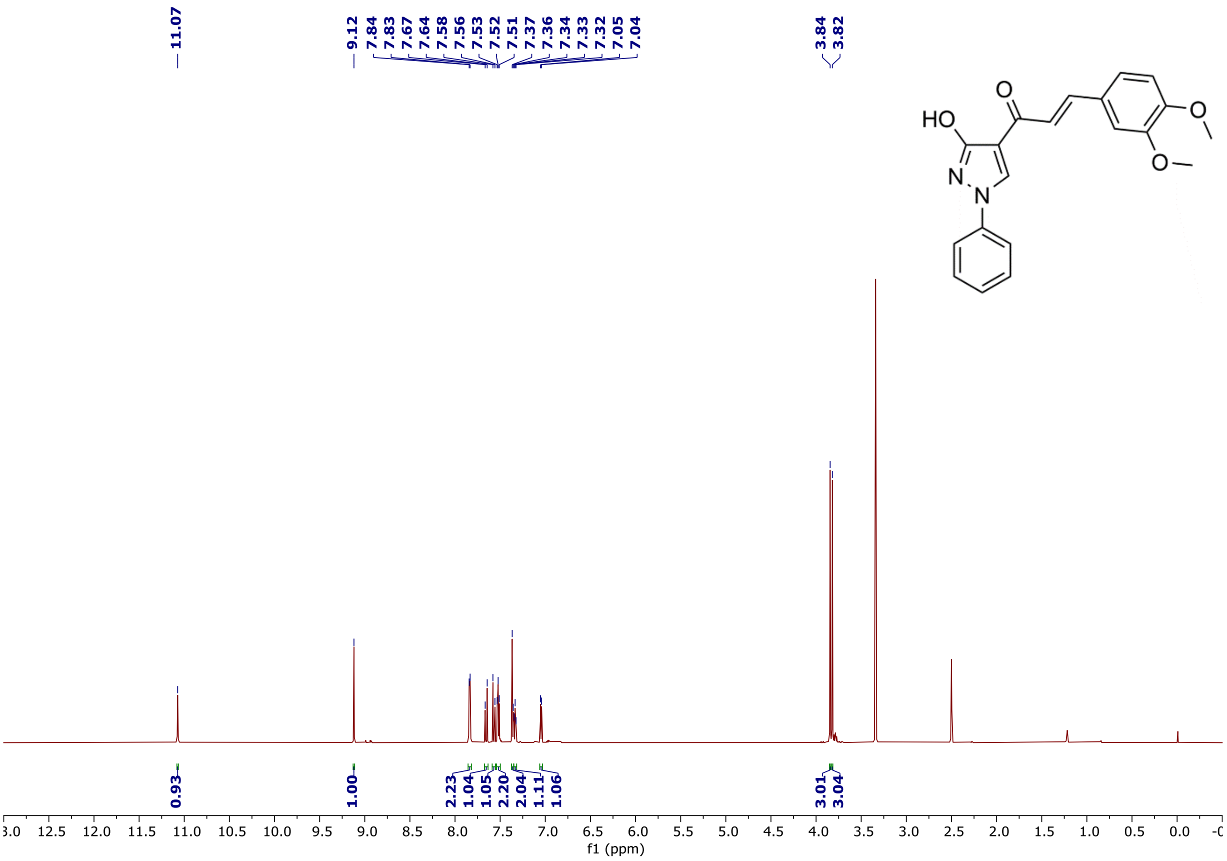

Figure S2. (2*E*)-3-(3,4-Dimethoxyphenyl)-1-(3-hydroxy-1-phenyl-1*H*-pyrazol-4-yl)prop-2-en-1-one 2d. ^{1}H NMR spectrum (700 MHz, DMSO-d_6)

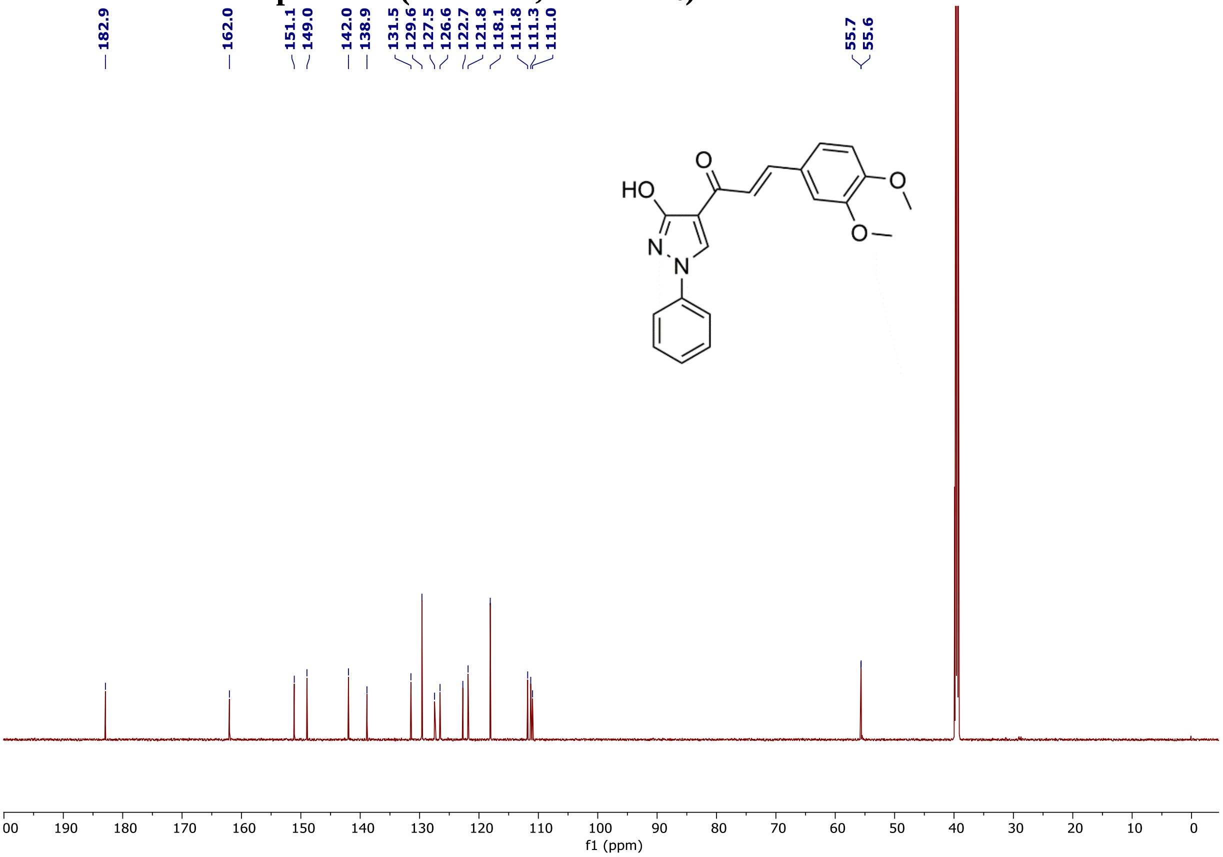

Figure S3. (2*E*)-3-(3,4-Dimethoxyphenyl)-1-(3-hydroxy-1-phenyl-1*H*-pyrazol-4-yl)prop-2-en-1-one 2d. ^{13}C NMR spectrum (176 MHz, DMSO-d_6)

Compound Spectrum SmartFormula Report

Analysis Info

Analysis Name	D:\Data\AUM-207.d	Acquisition Date	6/7/2023 7:47:51 PM
Method	DirectInfusion_TuneLow_pos.m	Operator	hplc
Sample Name	AUM-207	Instrument	microTOF-Q III 8228888.20448
Comment	AB		

Acquisition Parameter

Source Type	ESI	Ion Polarity	Positive	Set Nebulizer	0.4 Bar
Focus	Not active	Set Capillary	4500 V	Set Dry Heater	180 °C
Scan Begin	50 m/z	Set End Plate Offset	-500 V	Set Dry Gas	4.0 l/min
Scan End	1000 m/z	Set Collision Cell RF	140.0 Vpp	Set Divert Valve	Waste

#	RT [min]	Area	Int. Type	I	S/N	Chromatogram	Max. m/z	FWHM [min]
n.a.	3.5	n.a.	Single spectrum	n.a.	n.a.	n.a.	373.1162	n.a.

+MS, 3.5min #209

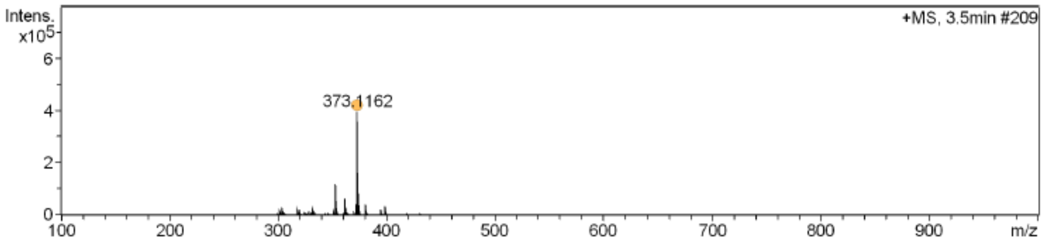

Meas. m/z	#	Ion Formula	m/z	err [ppm]	mSigma	# Sigma	Score	rdb	e⁻ Conf	N-Rule
373.1162	1	C20H18N2NaO4	373.1159	1.0	3.0	1	100.00	12.5	even	ok

Figure S4. (2*E*)-3-(3,4-Dimethoxyphenyl)-1-(3-hydroxy-1-phenyl-1*H*-pyrazol-4-yl)prop-2-en-1-one 2d. HRMS (ESI)

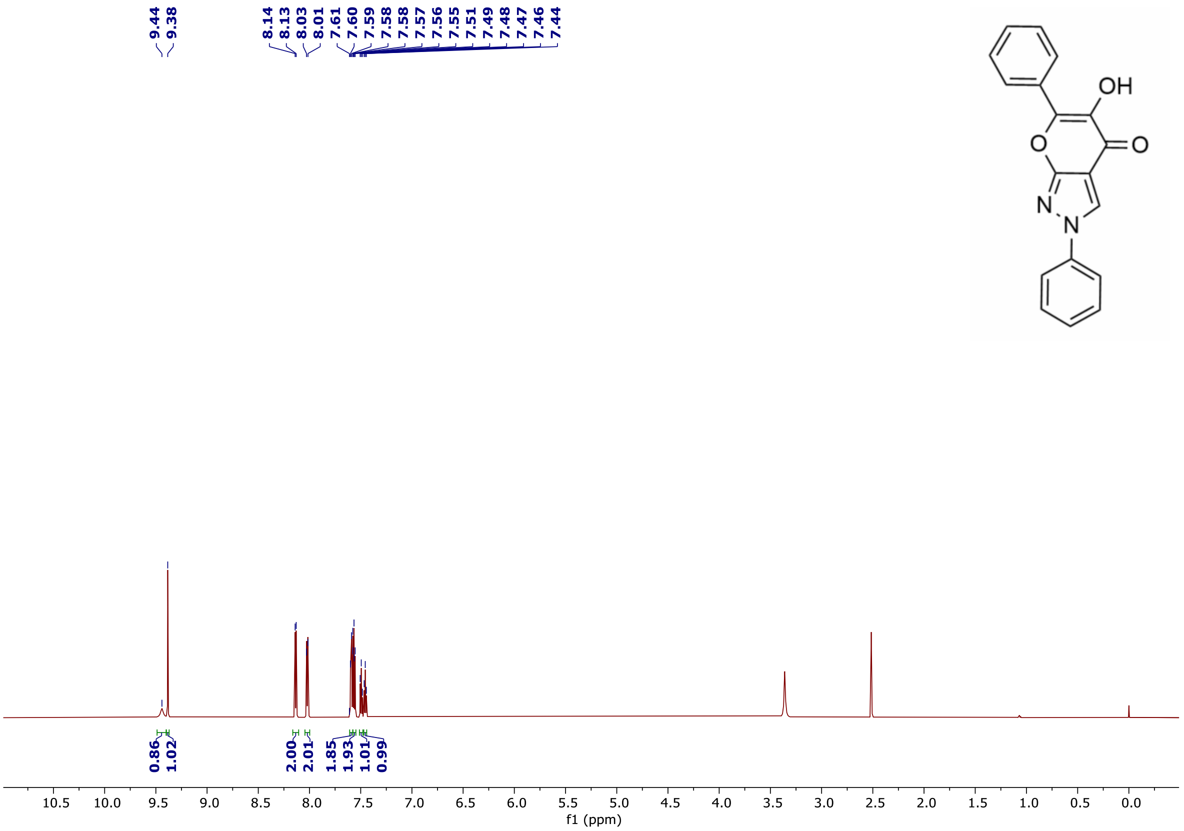

Figure S5. 5-Hydroxy-2,6-diphenylpyrano[2,3-*c*]pyrazol-4(2*H*)-one 3a. ^{1}H NMR spectrum (700 MHz, DMSO-*d*$_6$)

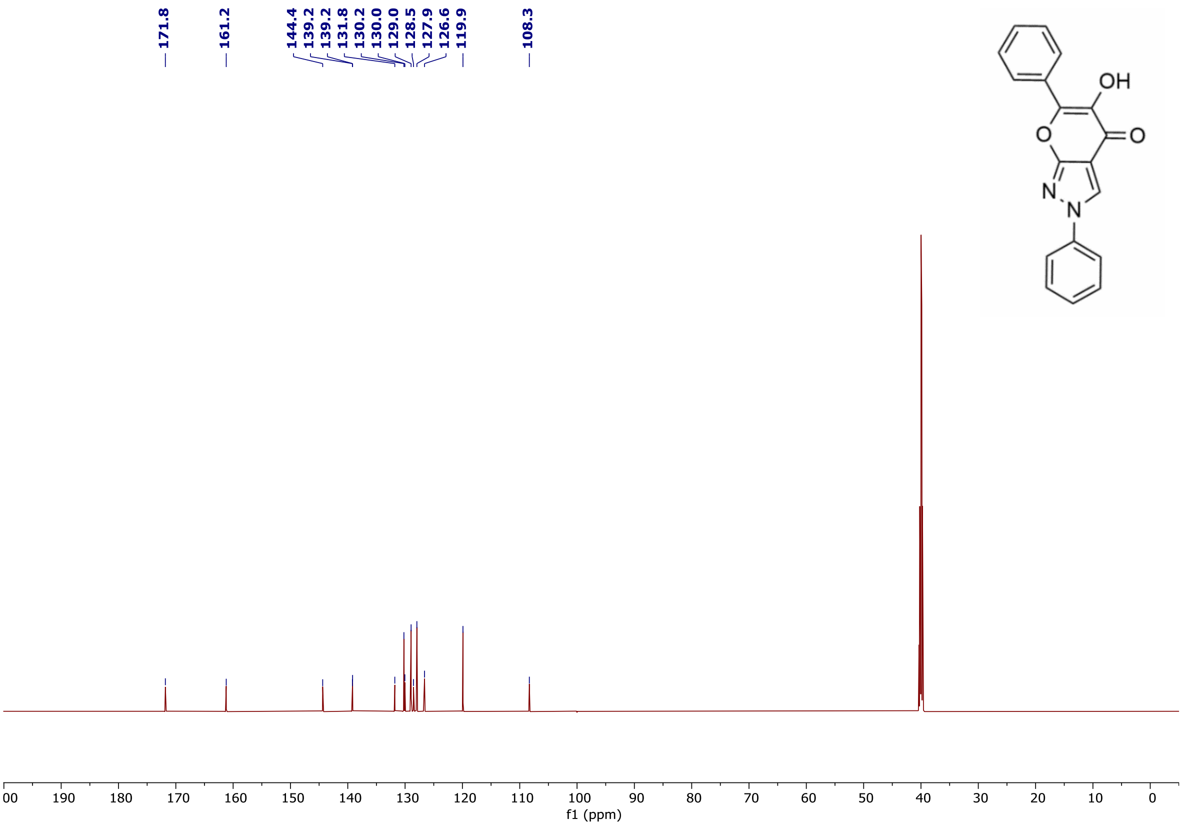

Figure S6. 5-Hydroxy-2,6-diphenylpyrano[2,3-*c*]pyrazol-4(2*H*)-one 3a. ^{13}C NMR spectrum (176 MHz, DMSO-*d*$_6$)

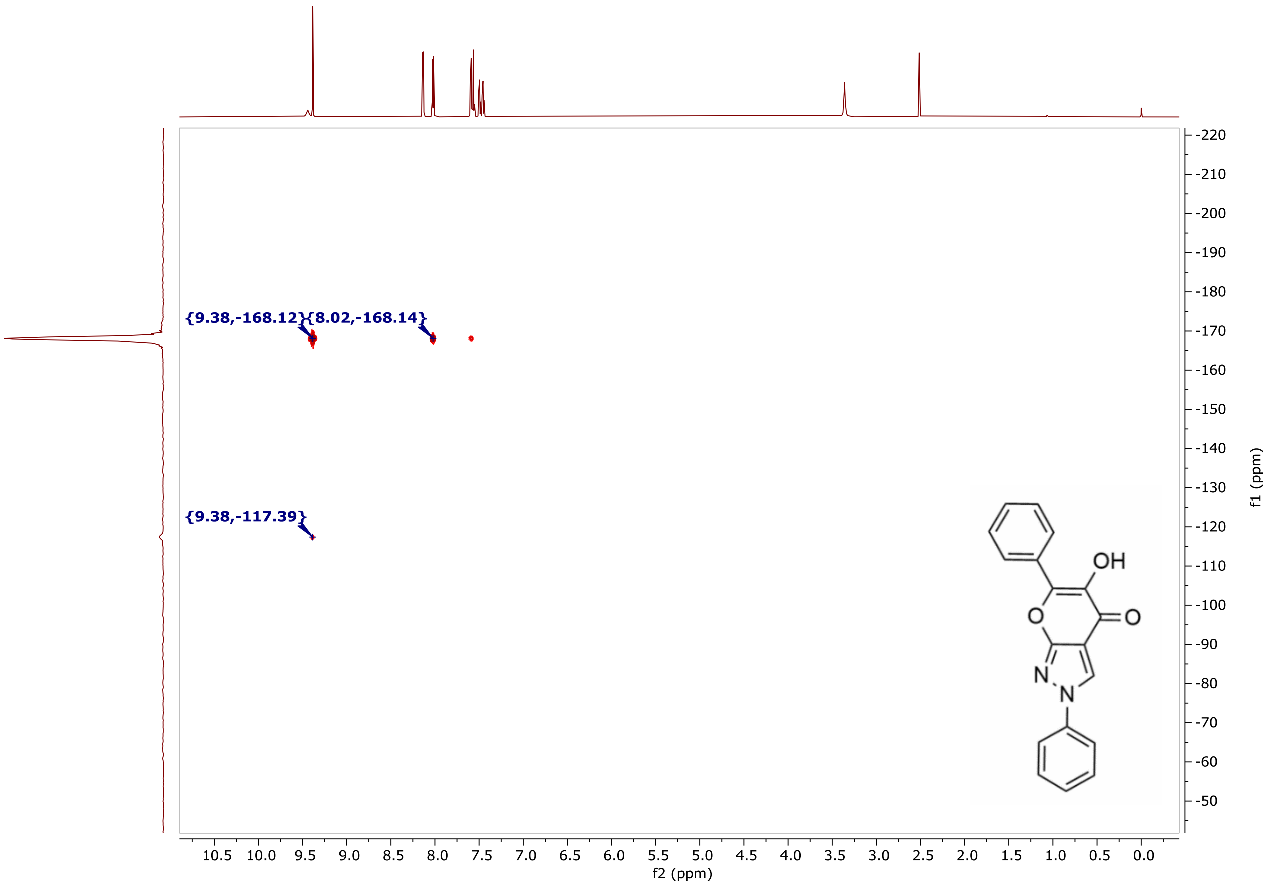

Figure S7. 5-Hydroxy-2,6-diphenylpyrano[2,3-*c*]pyrazol-4(2*H*)-one 3a. ^{1}H-^{15}N HMBC NMR spectrum (71 MHz, DMSO-d_6)

Compound Spectrum SmartFormula Report

Analysis Info

Analysis Name	D:\Data\AUM-103.d	Acquisition Date	11/6/2020 11:16:12 AM
Method	DirectInfusion_TuneLow_pos.m	Operator	hplc
Sample Name	AUM-103	Instrument	microTOF-Q III 8228888.20448
Comment	SB		

Acquisition Parameter

Source Type	ESI	Ion Polarity	Positive	Set Nebulizer	0.4 Bar
Focus	Not active	Set Capillary	4500 V	Set Dry Heater	180 °C
Scan Begin	50 m/z	Set End Plate Offset	-500 V	Set Dry Gas	4.0 l/min
Scan End	1000 m/z	Set Collision Cell RF	140.0 Vpp	Set Divert Valve	Waste

#	RT [min]	Area	Int. Type	I	S/N	Chromatogram	Max. m/z	FWHM [min]
n.a.	0.2	n.a.	Single spectrum	n.a.	n.a.	n.a.	226.9515	n.a.
n.a.	10.1	n.a.	Single spectrum	n.a.	n.a.	n.a.	327.0740	n.a.

+MS, 10.1min #605

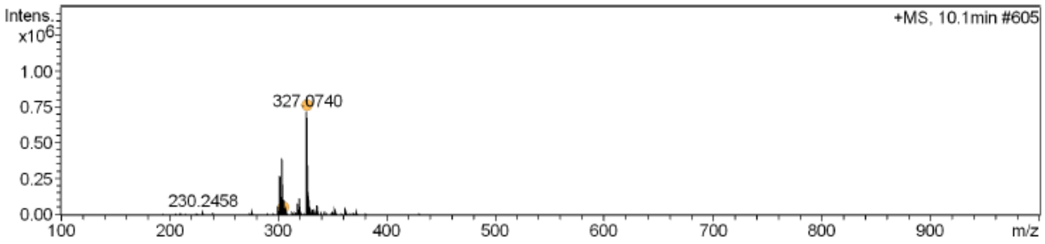

Meas. m/z	#	Ion Formula	m/z	err [ppm]	mSigma	# Sigma	Score	rdb	e⁻ Conf	N-Rule
305.0915	1	C18H13N2O3	305.0921	2.0	65.3	1	100.00	13.5	even	ok
327.0740	1	C18H12N2NaO3	327.0740	0.1	14.6	1	100.00	13.5	even	ok

Figure S8. 5-Hydroxy-2,6-diphenylpyrano[2,3-*c*]pyrazol-4(2*H*)-one 3a. HRMS (ESI)

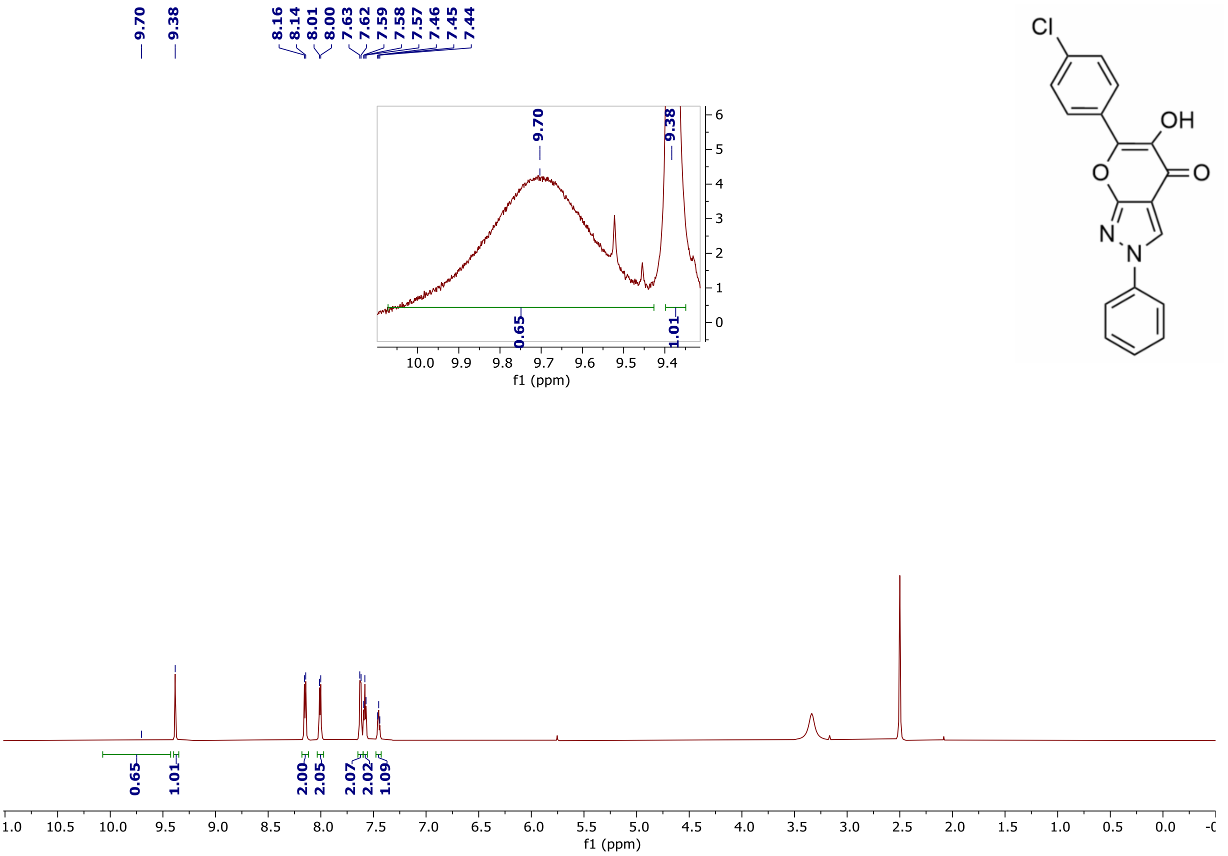

Figure S9. 6-(4-Chlorophenyl)-5-hydroxy-2-phenylpyrano[2,3-*c*]pyrazol-4(2*H*)-one 3b. ^{1}H NMR spectrum (700 MHz, DMSO-*d*$_6$)

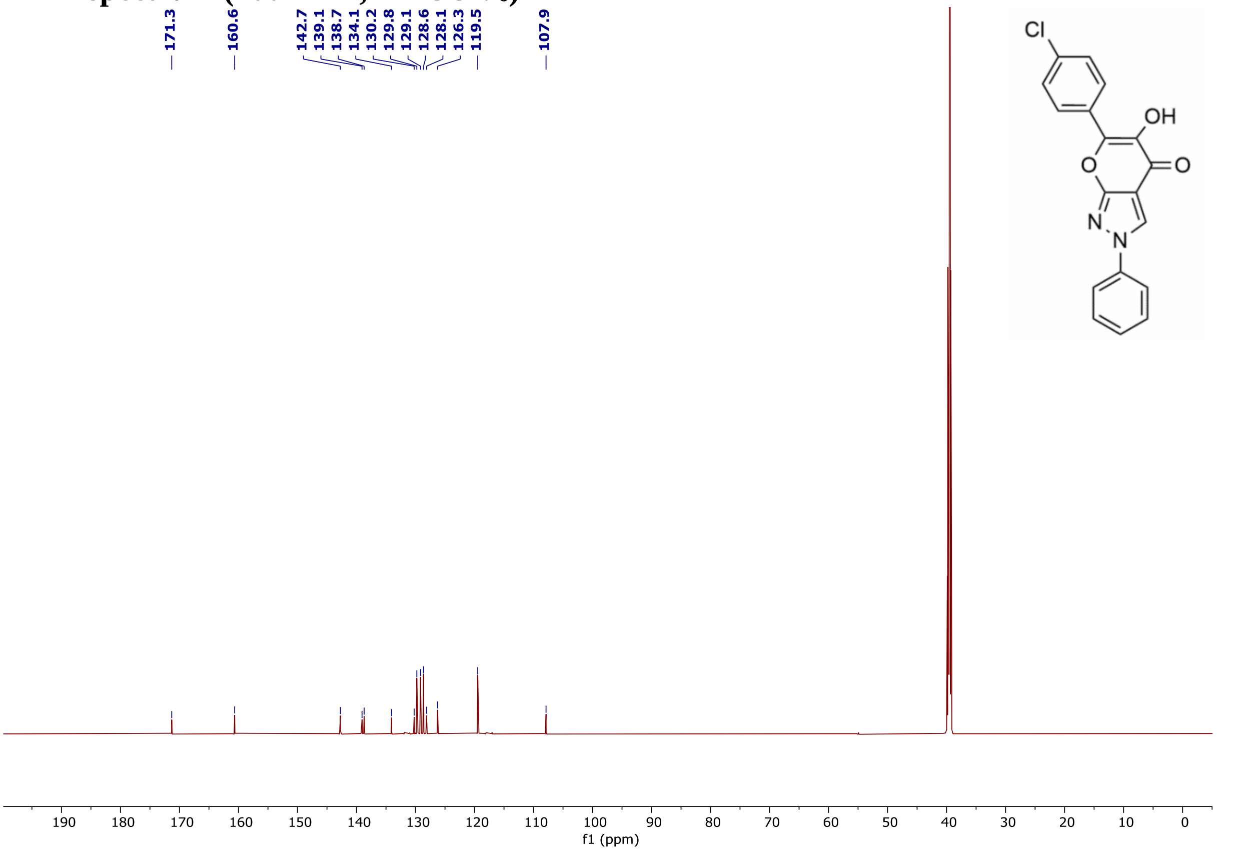

Figure S10. 6-(4-Chlorophenyl)-5-hydroxy-2-phenylpyrano[2,3-*c*]pyrazol-4(2*H*)-one 3b. ^{13}C NMR spectrum (176 MHz, DMSO-*d*$_6$)

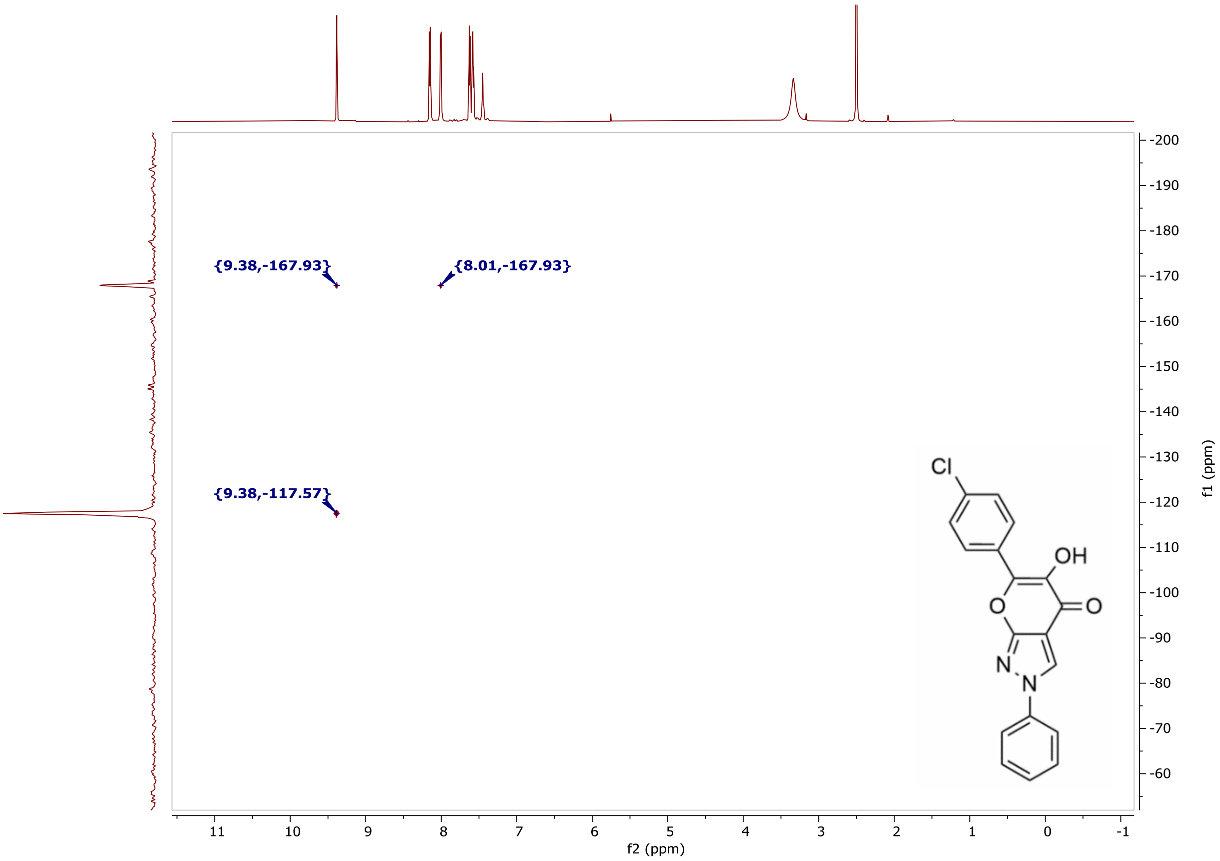

Figure S11. 6-(4-Chlorophenyl)-5-hydroxy-2-phenylpyrano[2,3-*c*]pyrazol-4(2*H*)-one 3b. ^{1}H-^{15}N HMBC NMR spectrum (71 MHz, DMSO-*d*$_6$)

Compound Spectrum SmartFormula Report

Analysis Info

Analysis Name	D:\Data\AUM-112.d	Acquisition Date	11/10/2020 1:15:04 PM
Method	DirectInfusion_TuneLow_pos.m	Operator	hplc
Sample Name	AUM-112	Instrument	microTOF-Q III 8228888.20448
Comment	SB		

Acquisition Parameter

Source Type	ESI	Ion Polarity	Positive	Set Nebulizer	0.4 Bar
Focus	Not active	Set Capillary	4500 V	Set Dry Heater	180 °C
Scan Begin	50 m/z	Set End Plate Offset	-500 V	Set Dry Gas	4.0 l/min
Scan End	1000 m/z	Set Collision Cell RF	140.0 Vpp	Set Divert Valve	Waste

#	RT [min]	Area	Int. Type	I	S/N	Chromatogram	Max. m/z	FWHM [min]
n.a.	0.6	n.a.	Single spectrum	n.a.	n.a.	n.a.	226.9520	n.a.
n.a.	10.2	n.a.	Single spectrum	n.a.	n.a.	n.a.	361.0350	n.a.

+MS, 10.2min #614

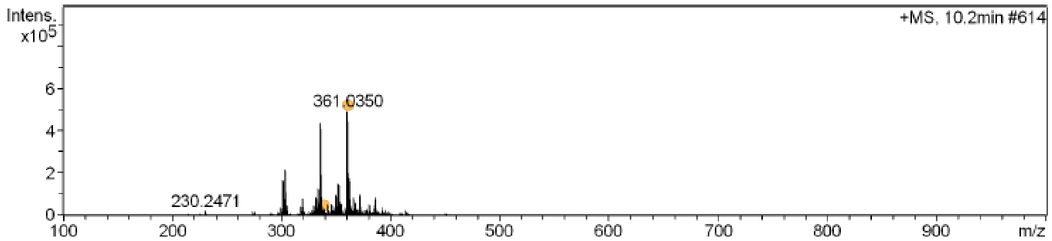

Meas. m/z	#	Ion Formula	m/z	err [ppm]	mSigma	# Sigma	Score	rdb	e⁻ Conf	N-Rule
339.0532	1	C18H12ClN2O3	339.0531	-0.2	45.6	1	100.00	13.5	even	ok
361.0350	1	C18H11ClN2NaO3	361.0350	-0.0	6.9	1	100.00	13.5	even	ok

Figure S12. 6-(4-Chlorophenyl)-5-hydroxy-2-phenylpyrano[2,3-*c*]pyrazol-4(2*H*)-one 3b. HRMS (ESI)

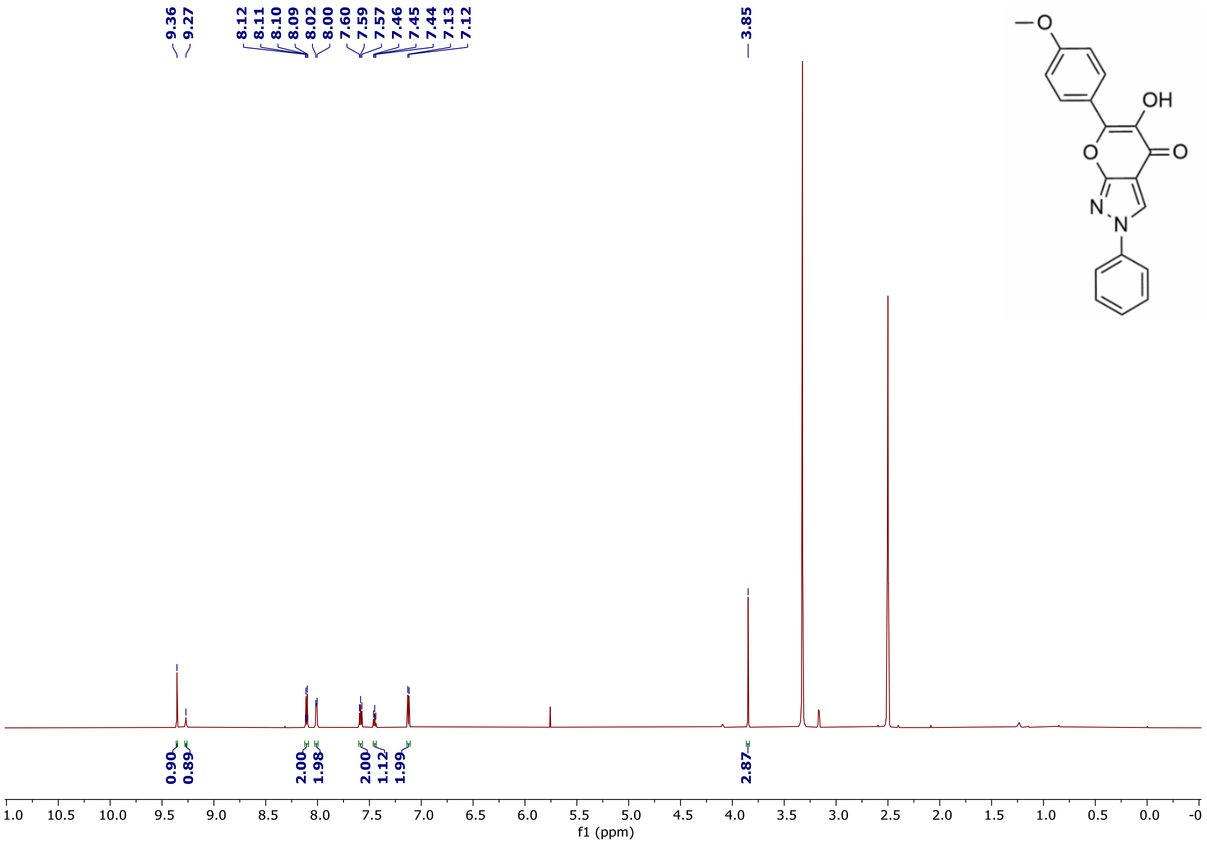

Figure S13. 5-Hydroxy-6-(4-methoxyphenyl)-2-phenylpyrano[2,3-*c*]pyrazol-4(2*H*)-one 3c. ^{1}H NMR spectrum (700 MHz, DMSO-d_6)

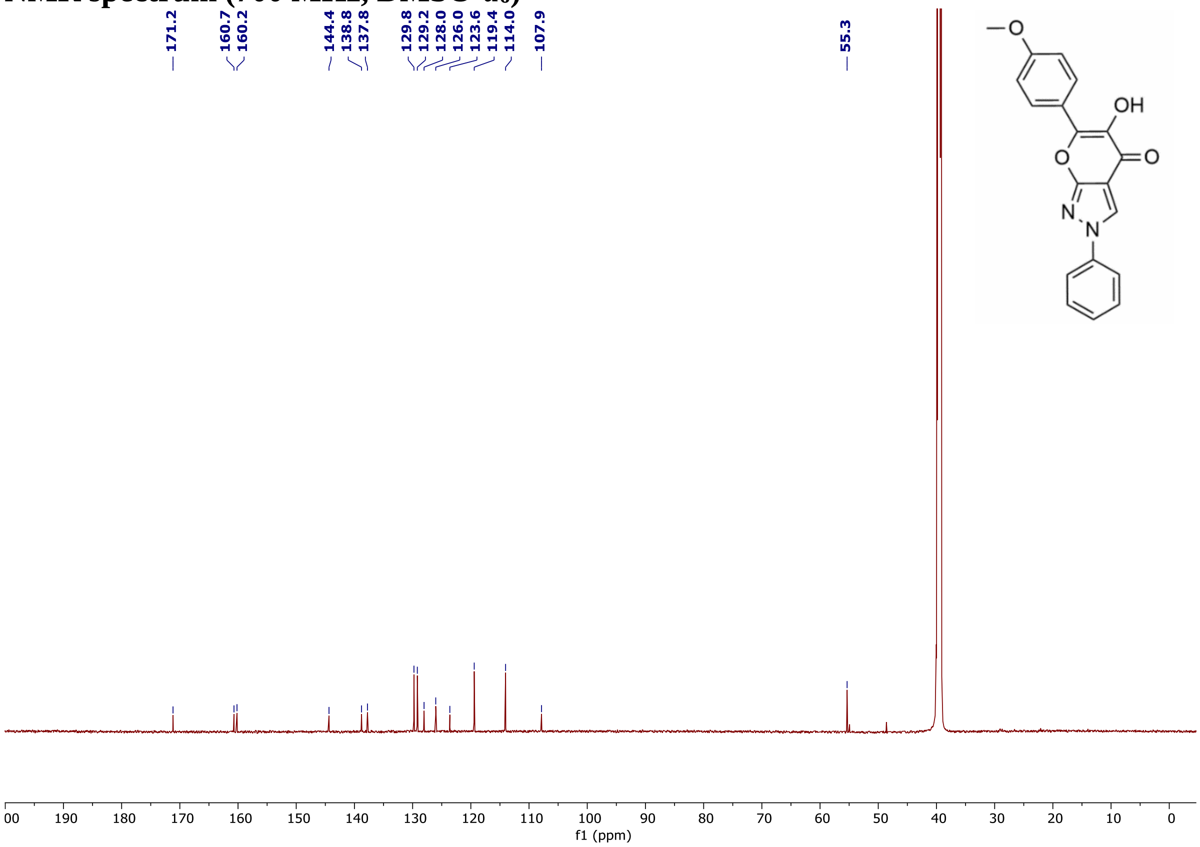

Figure S14. 5-Hydroxy-6-(4-methoxyphenyl)-2-phenylpyrano[2,3-*c*]pyrazol-4(2*H*)-one 3c. ^{13}C NMR spectrum (176 MHz, DMSO-d_6)

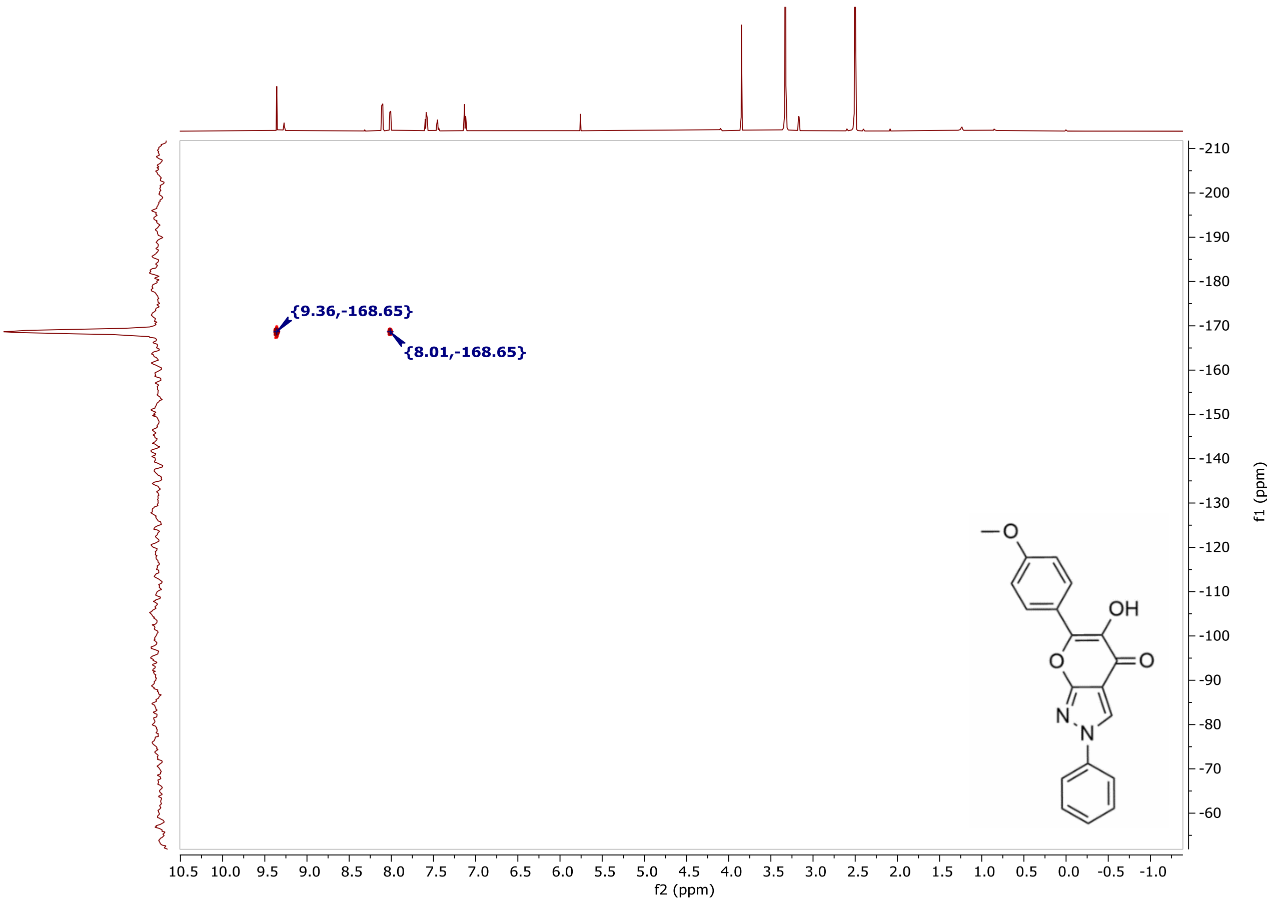

Figure S15. 5-Hydroxy-6-(4-methoxyphenyl)-2-phenylpyrano[2,3-*c*]pyrazol-4(2*H*)-one 3c. ^{1}H-^{15}N HMBC NMR spectrum (71 MHz, DMSO-*d*$_6$)

Compound Spectrum SmartFormula Report

Analysis Info

Analysis Name	D:\Data\AUM-108_new_positive4.d	Acquisition Date	6/7/2023 10:42:41 PM
Method	DirectInfusion_TuneLow_pos.m	Operator	hplc
Sample Name	AUM-108_new_positive4	Instrument	micrOTOF-Q III 8228888.20448
Comment	AB		

Acquisition Parameter

Source Type	ESI	Ion Polarity	Positive	Set Nebulizer	0.4 Bar
Focus	Not active	Set Capillary	4500 V	Set Dry Heater	180 °C
Scan Begin	50 m/z	Set End Plate Offset	-500 V	Set Dry Gas	4.0 l/min
Scan End	1000 m/z	Set Collision Cell RF	250.0 Vpp	Set Divert Valve	Waste

#	RT [min]	Area	Int. Type	I	S/N	Chromatogram	Max. m/z	FWHM [min]
n.a.	2.7	n.a.	Single spectrum	n.a.	n.a.	n.a.	497.3157	n.a.

+MS, 2.7min #162

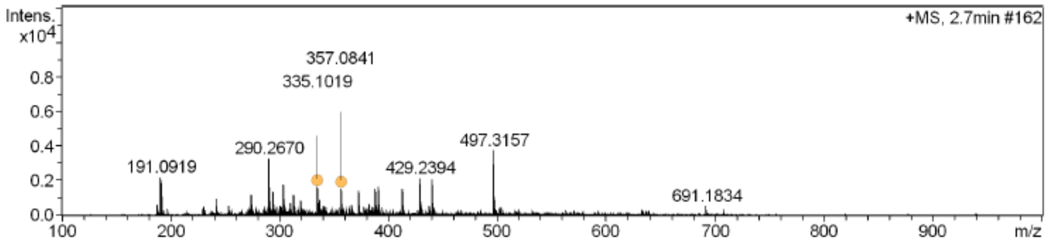

Meas. m/z	#	Ion Formula	m/z	err [ppm]	mSigma	# Sigma	Score	rdb	e⁻ Conf	N-Rule
335.1019	1	C19H15N2O4	335.1026	-2.3	290.0	1	100.00	13.5	even	ok
357.0841	1	C19H14N2NaO4	357.0846	-1.4	112.3	1	100.00	13.5	even	ok

Figure S16. 5-Hydroxy-6-(4-methoxyphenyl)-2-phenylpyrano[2,3-*c*]pyrazol-4(2*H*)-one 3c. HRMS (ESI)

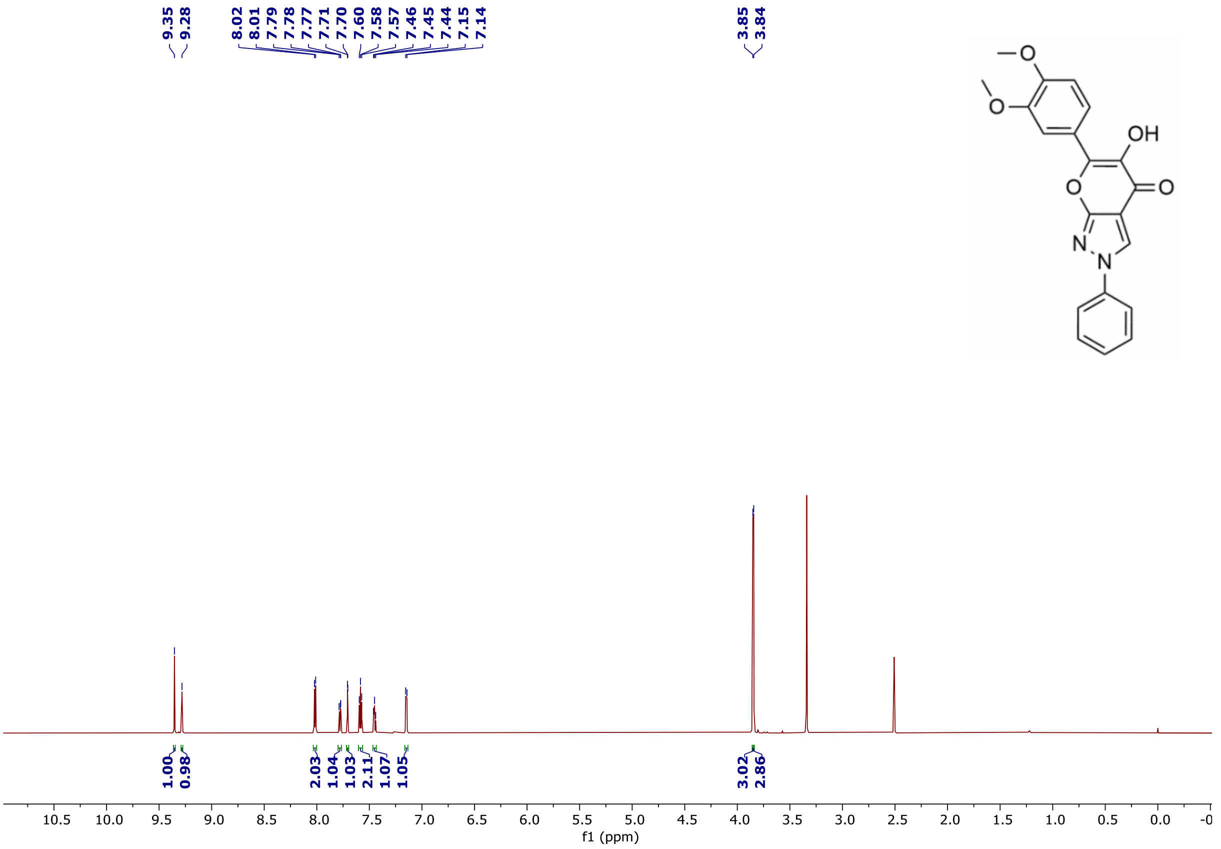

Figure S17. 6-(3,4-Dimethoxyphenyl)-5-hydroxy-2-phenylpyrano[2,3-*c*]pyrazol-4(2*H*)-one 3d. ^{1}H NMR spectrum (700 MHz, DMSO-*d*$_6$)

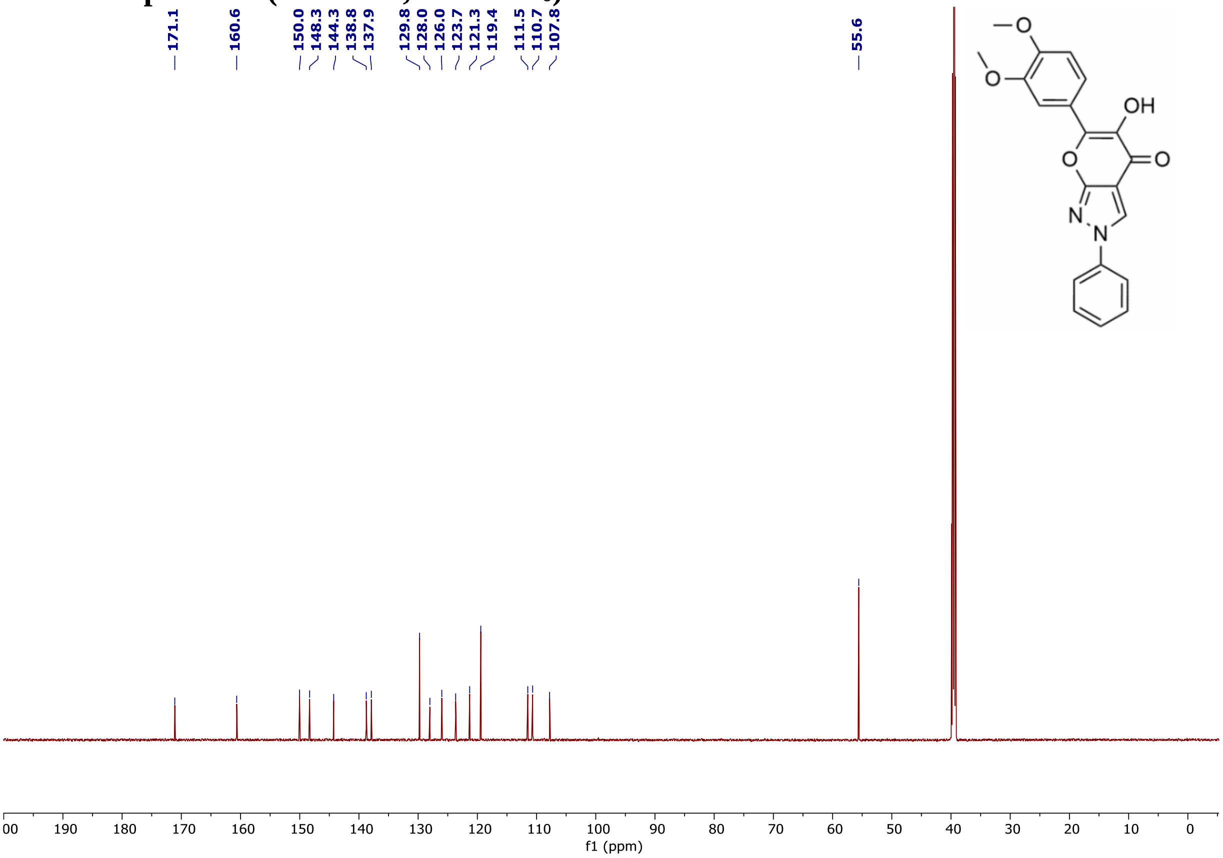

Figure S18. 6-(3,4-Dimethoxyphenyl)-5-hydroxy-2-phenylpyrano[2,3-*c*]pyrazol-4(2*H*)-one 3d. ^{13}C NMR spectrum (176 MHz, DMSO-*d*$_6$)

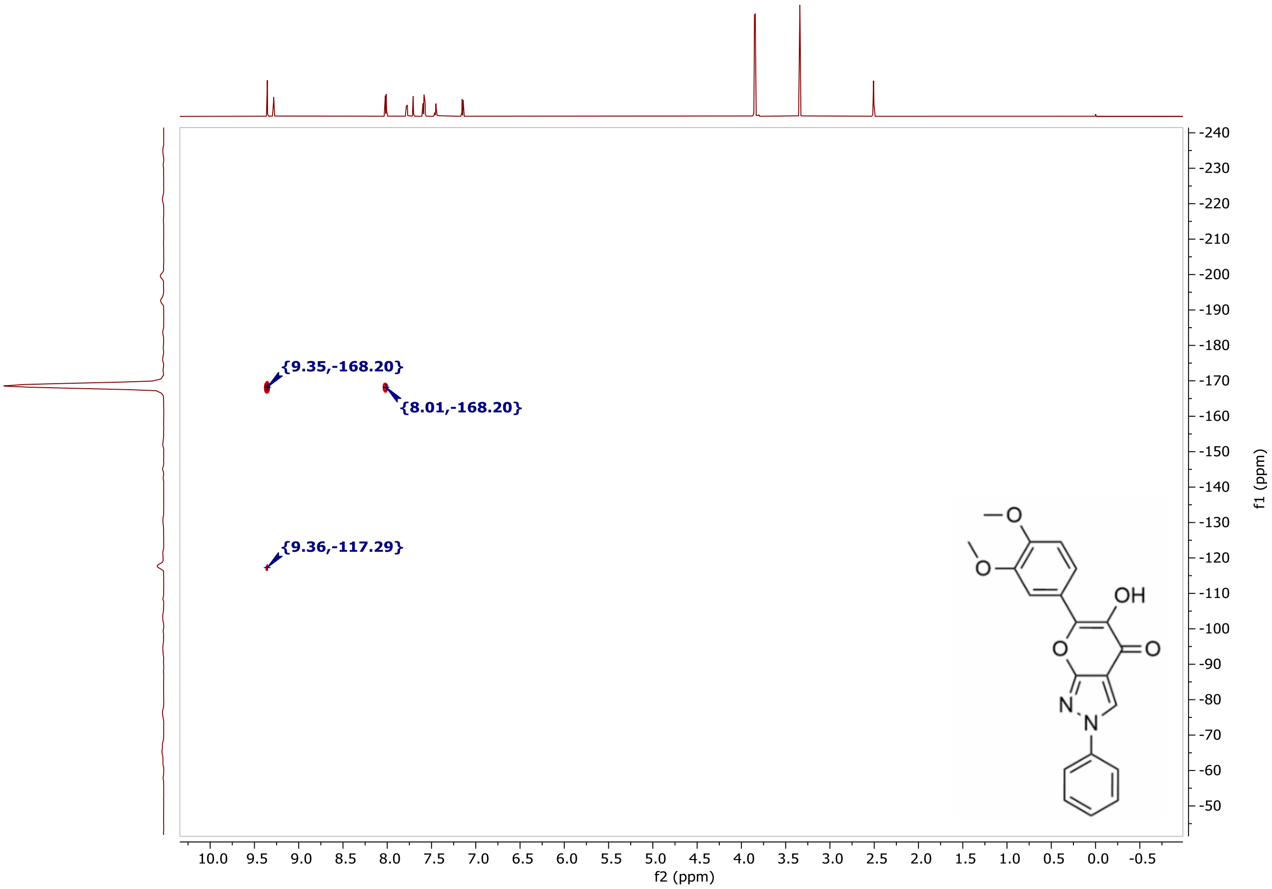

Figure S19. 6-(3,4-Dimethoxyphenyl)-5-hydroxy-2-phenylpyrano[2,3-*c*]pyrazol-4(2*H*)-one 3d. ^{1}H-^{15}N HMBC NMR spectrum (71 MHz, DMSO-*d*$_6$)

Compound Spectrum SmartFormula Report

Analysis Info

Analysis Name	D:\Data\AUM-211.d	Acquisition Date	6/7/2023 7:09:27 PM
Method	DirectInfusion_TuneLow_pos.m	Operator	hplc
Sample Name	AUM-211	Instrument	microTOF-Q III 8228888.20448
Comment	AB		

Acquisition Parameter

Source Type	ESI	Ion Polarity	Positive	Set Nebulizer	0.4 Bar
Focus	Not active	Set Capillary	4500 V	Set Dry Heater	180 °C
Scan Begin	50 m/z	Set End Plate Offset	-500 V	Set Dry Gas	4.0 l/min
Scan End	1000 m/z	Set Collision Cell RF	140.0 Vpp	Set Divert Valve	Waste

#	RT [min]	Area	Int. Type	I	S/N	Chromatogram	Max. m/z	FWHM [min]
n.a.	15.5	n.a.	Single spectrum	n.a.	n.a.	n.a.	387.0953	n.a.

+MS, 15.5min #929

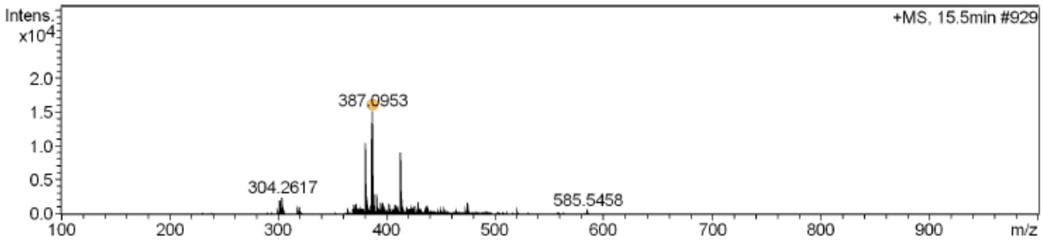

Meas. m/z	#	Ion Formula	m/z	err [ppm]	mSigma	# Sigma	Score	rdb	e¯ Conf	N-Rule
387.0953	1	C20H16N2NaO5	387.0951	0.3	7.2	1	100.00	13.5	even	ok

Figure S20 6-(3,4-Dimethoxyphenyl)-5-hydroxy-2-phenylpyrano[2,3-*c*]pyrazol-4(2*H*)-one 3d. HRMS (ESI)

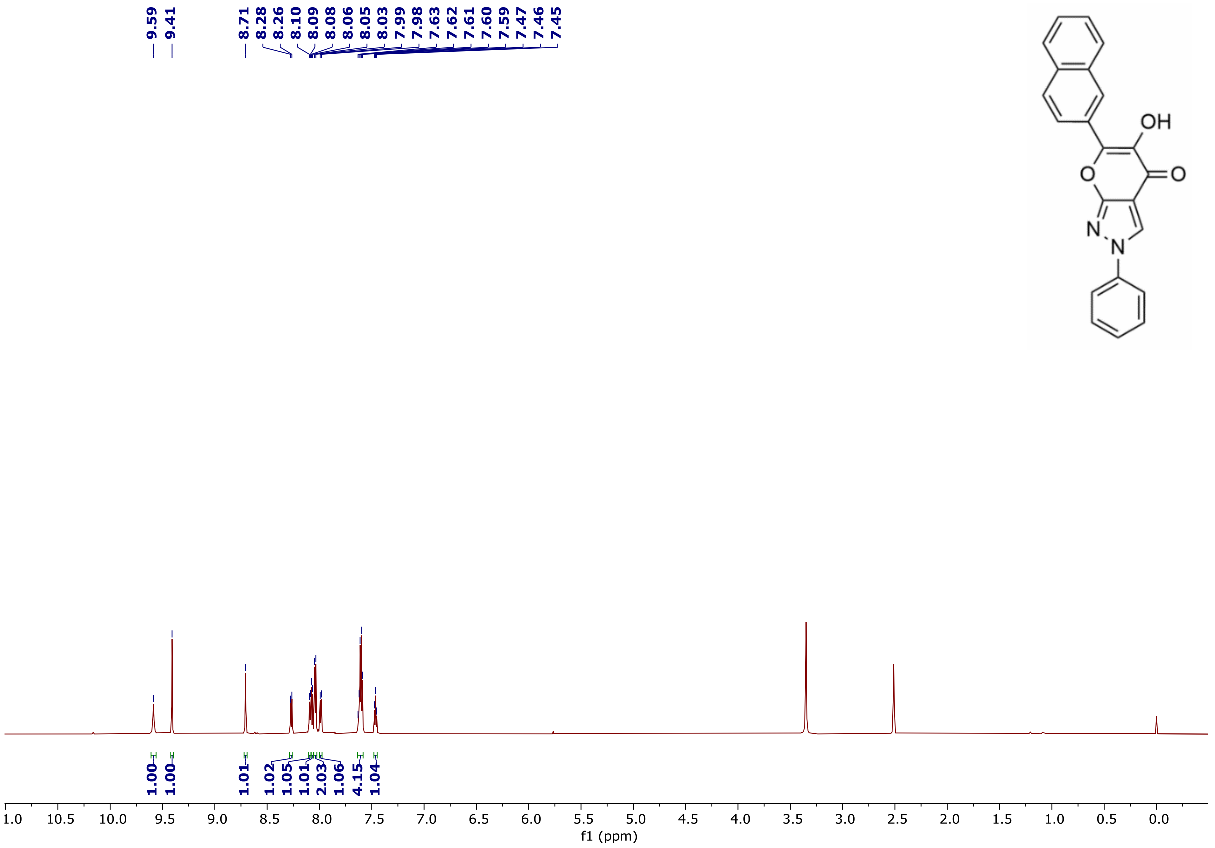

Figure S21. 5-Hydroxy-6-(naphthalen-2-yl)-2-phenylpyrano[2,3-*c*]pyrazol-4(2*H*)-one 3e. ^{1}H NMR spectrum (700 MHz, DMSO-*d*$_6$)

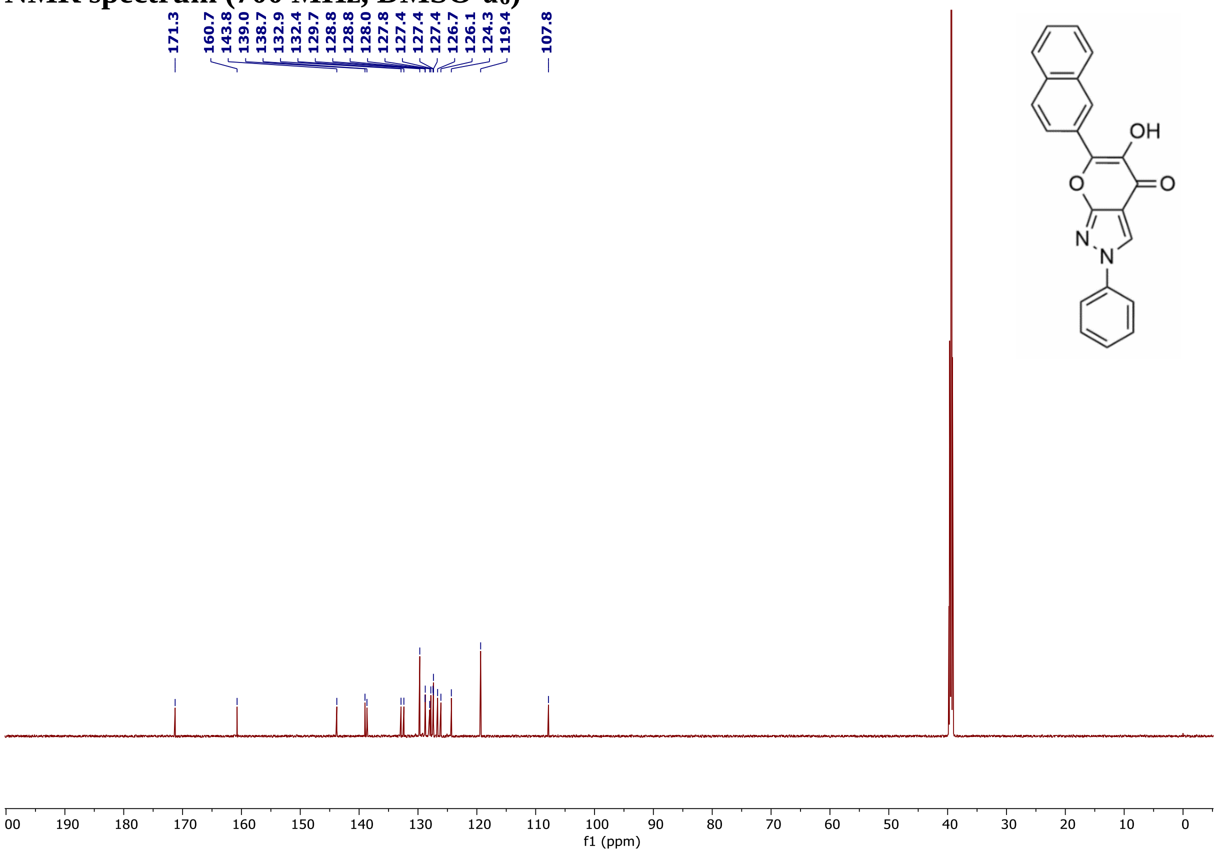

Figure S22. 5-Hydroxy-6-(naphthalen-2-yl)-2-phenylpyrano[2,3-*c*]pyrazol-4(2*H*)-one 3e. ^{13}C NMR spectrum (176 MHz, DMSO-*d*$_6$)

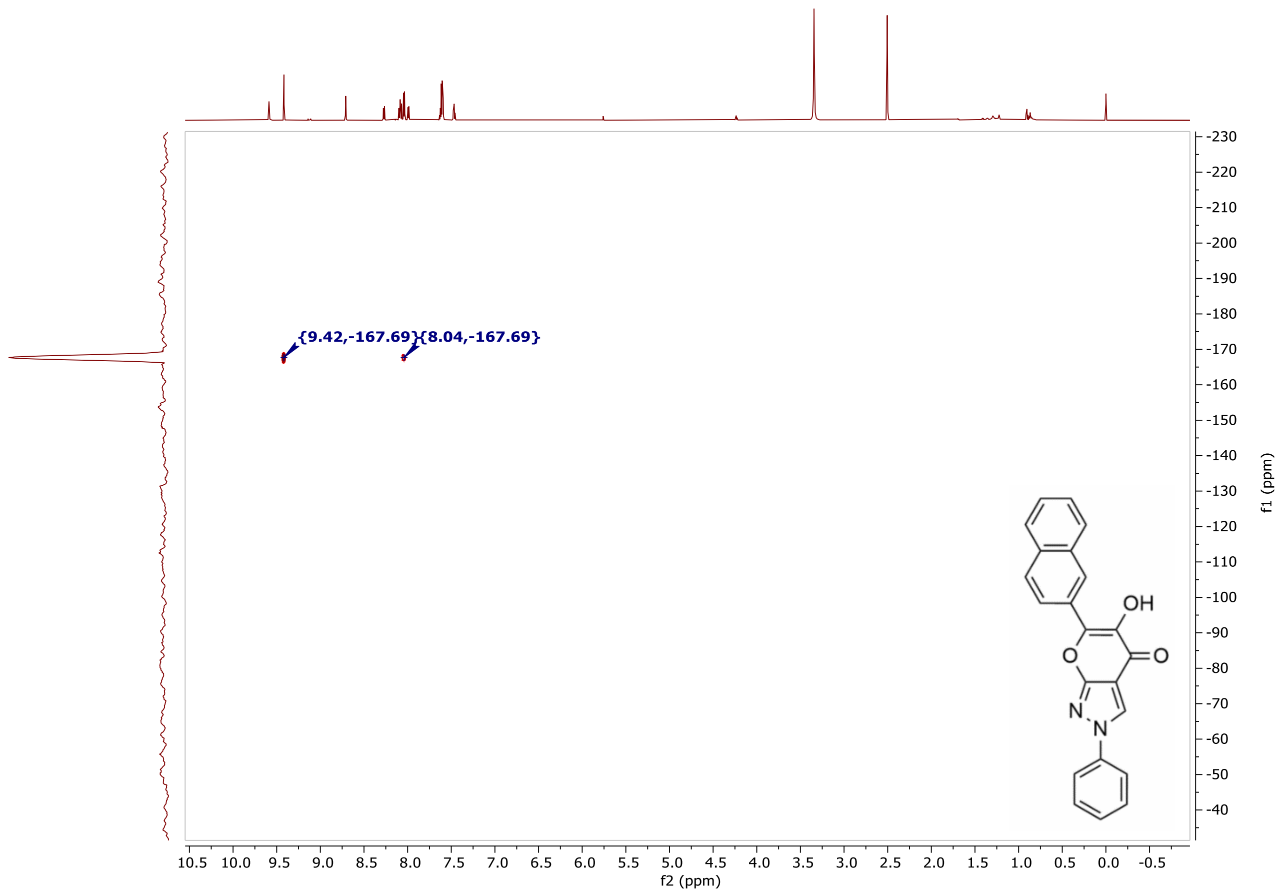

Figure S23. 5-Hydroxy-6-(naphthalen-2-yl)-2-phenylpyrano[2,3-*c*]pyrazol-4(2*H*)-one 3e. ^{1}H-^{15}N HMBC NMR spectrum (71 MHz, DMSO-d_6)

Compound Spectrum SmartFormula Report

Analysis Info

Analysis Name	D:\Data\AUM-193-2.d	Acquisition Date	6/7/2023 4:09:43 PM
Method	DirectInfusion_TuneLow_pos.m	Operator	hplc
Sample Name	AUM-193-2	Instrument	micrOTOF-Q III 8228888.20448
Comment	AB		

Acquisition Parameter

Source Type	ESI	Ion Polarity	Positive	Set Nebulizer	0.4 Bar
Focus	Not active	Set Capillary	4500 V	Set Dry Heater	180 °C
Scan Begin	50 m/z	Set End Plate Offset	-500 V	Set Dry Gas	4.0 l/min
Scan End	1000 m/z	Set Collision Cell RF	377.0 Vpp	Set Divert Valve	Waste

#	RT [min]	Area	Int. Type	I	S/N	Chromatogram	Max. m/z	FWHM [min]
n.a.	0.2	n.a.	Single spectrum	n.a.	n.a.	n.a.	413.2688	n.a.

+MS, 0.2min #11

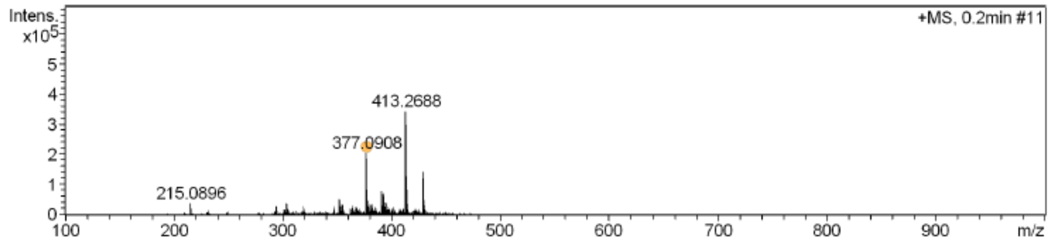

Meas. m/z	#	Ion Formula	m/z	err [ppm]	mSigma	# Sigma	Score	rdb	e¯ Conf	N-Rule
377.0908	1	C22H14N2NaO3	377.0897	-3.1	50.6	2	100.00	16.5	even	ok

Figure S24. 5-Hydroxy-6-(naphthalen-2-yl)-2-phenylpyrano[2,3-*c*]pyrazol-4(2*H*)-one 3e. HRMS (ESI)

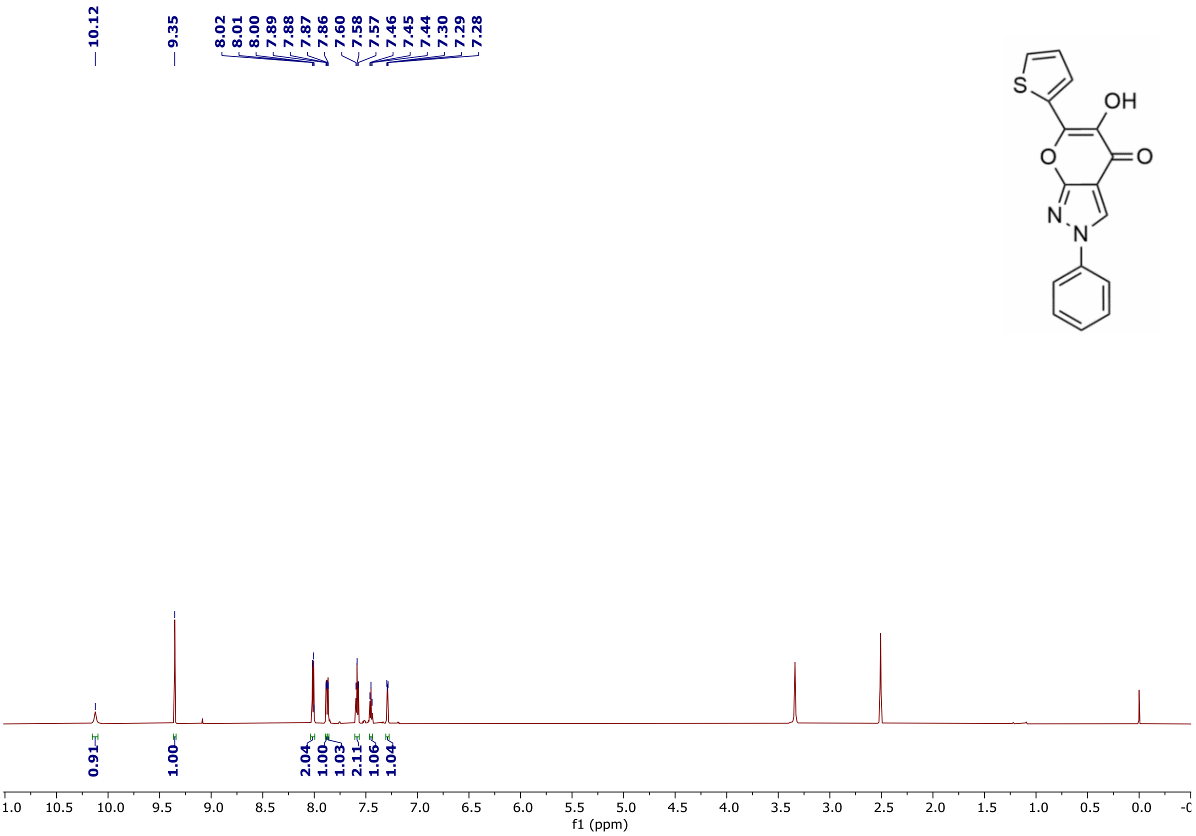

Figure S25. 5-Hydroxy-2-phenyl-6-(thiophen-2-yl)pyrano[2,3-*c*]pyrazol-4(2*H*)-one 3f. ^{1}H NMR spectrum (700 MHz, DMSO-*d*$_6$)

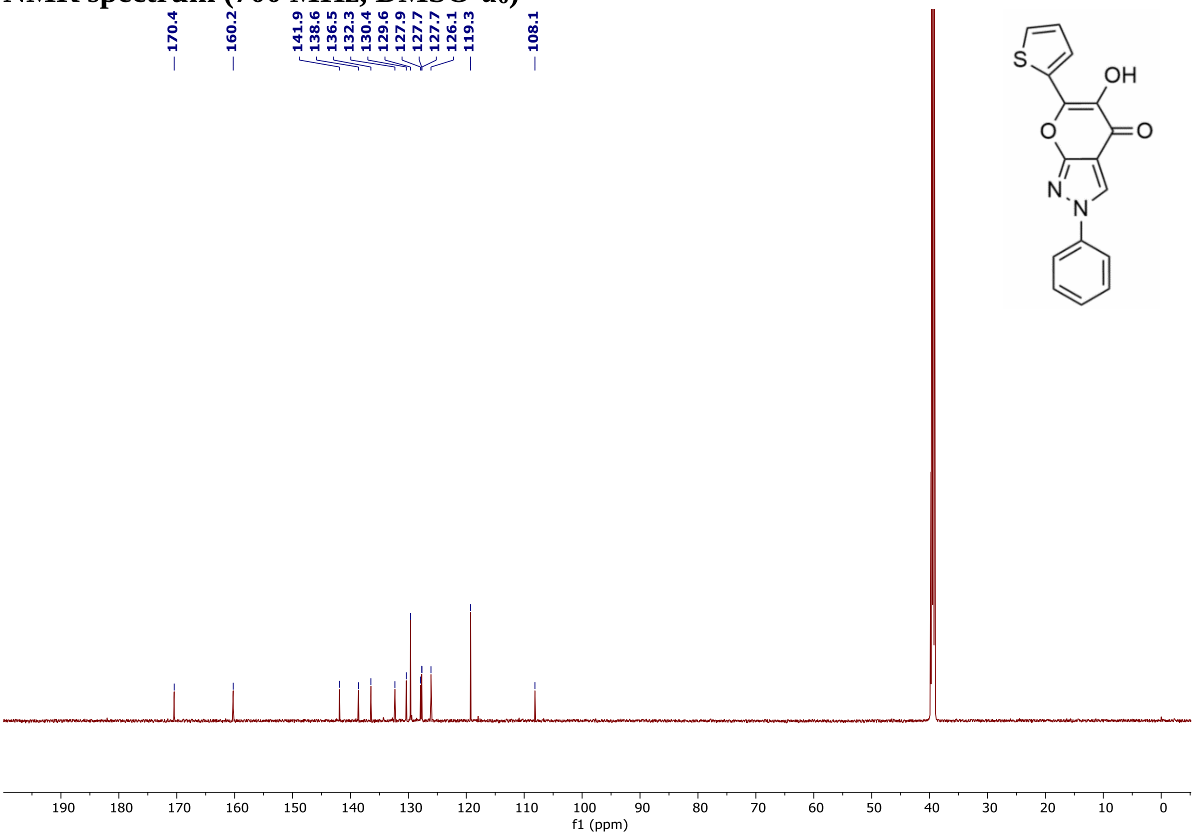

Figure S26. 5-Hydroxy-2-phenyl-6-(thiophen-2-yl)pyrano[2,3-*c*]pyrazol-4(2*H*)-one 3f. ^{13}C NMR spectrum (176 MHz, DMSO-*d*$_6$)

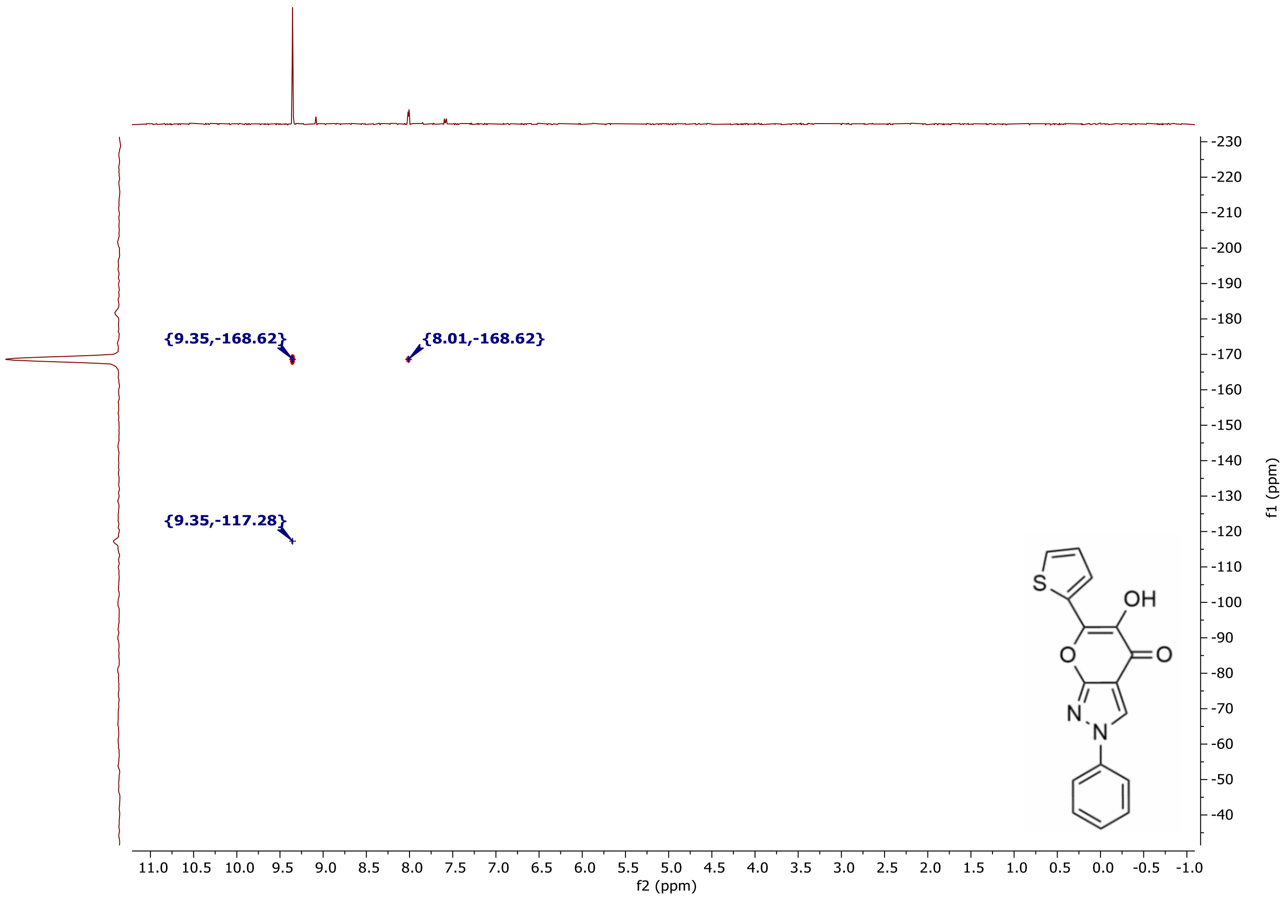

Figure S27. 5-Hydroxy-2-phenyl-6-(thiophen-2-yl)pyrano[2,3-*c*]pyrazol-4(2*H*)-one 3f. ^{1}H-^{15}N HMBC NMR spectrum (71 MHz, DMSO-*d*$_6$)

Compound Spectrum SmartFormula Report

Analysis Info

Analysis Name	D:\Data\AUM-190.d	Acquisition Date	6/7/2023 8:08:41 PM
Method	DirectInfusion_TuneLow_pos.m	Operator	hplc
Sample Name	AUM-190	Instrument	micrOTOF-Q III 8228888.20448
Comment	AB		

Acquisition Parameter

Source Type	ESI	Ion Polarity	Positive	Set Nebulizer	0.4 Bar
Focus	Not active	Set Capillary	4500 V	Set Dry Heater	180 °C
Scan Begin	50 m/z	Set End Plate Offset	-500 V	Set Dry Gas	4.0 l/min
Scan End	1000 m/z	Set Collision Cell RF	140.0 Vpp	Set Divert Valve	Waste

#	RT [min]	Area	Int. Type	I	S/N	Chromatogram	Max. m/z	FWHM [min]
n.a.	12.9	n.a.	Single spectrum	n.a.	n.a.	n.a.	333.0309	n.a.

+MS, 12.9min #775

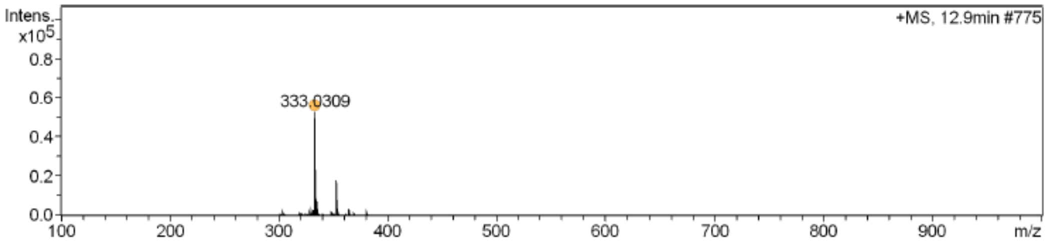

Meas. m/z	#	Ion Formula	m/z	err [ppm]	mSigma	# Sigma	Score	rdb	e⁻ Conf	N-Rule
333.0309	1	C16H10N2NaO3S	333.0304	1.4	35.6	1	100.00	12.5	even	ok

Figure S28. 5-Hydroxy-2-phenyl-6-(thiophen-2-yl)pyrano[2,3-*c*]pyrazol-4(2*H*)-one 3f. HRMS (ESI)

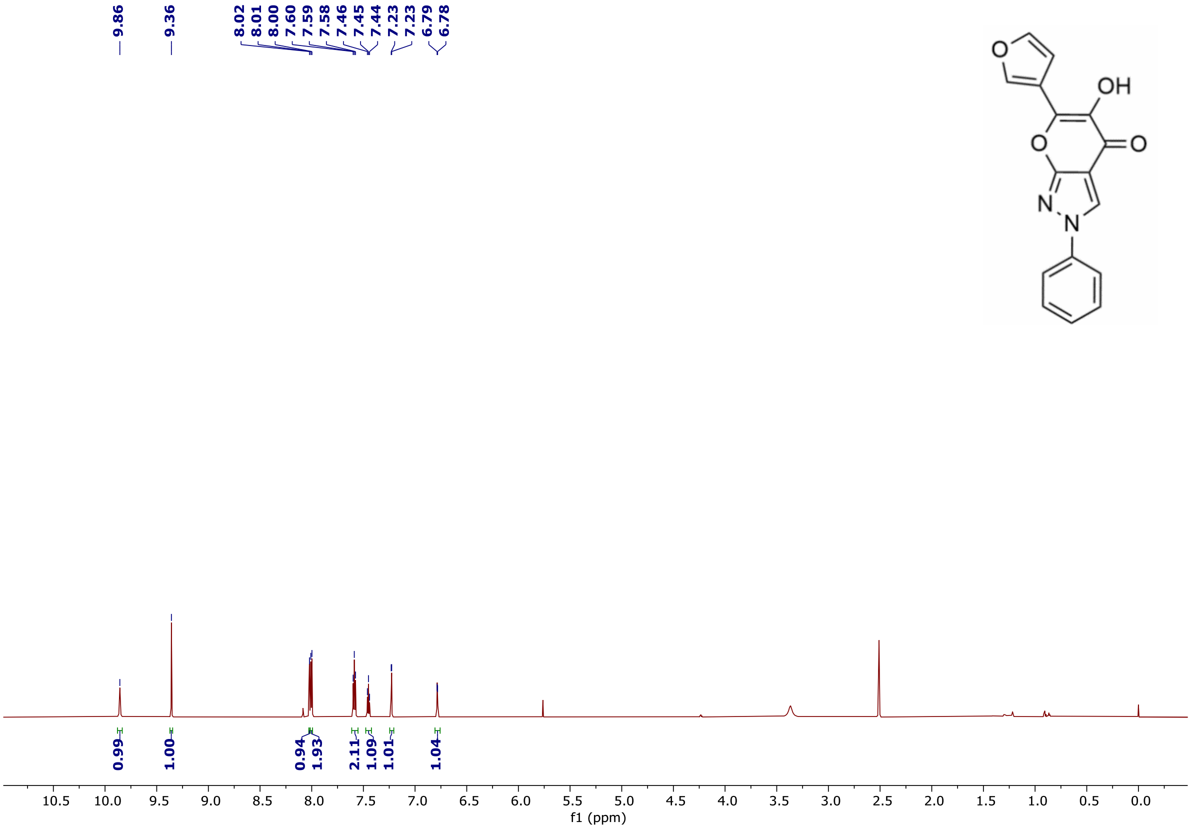

Figure S29. 6-(Furan-3-yl)-5-hydroxy-2-phenylpyrano[2,3-*c*]pyrazol-4(2*H*)-one 3g. ^{1}H NMR spectrum (700 MHz, DMSO-*d*$_6$)

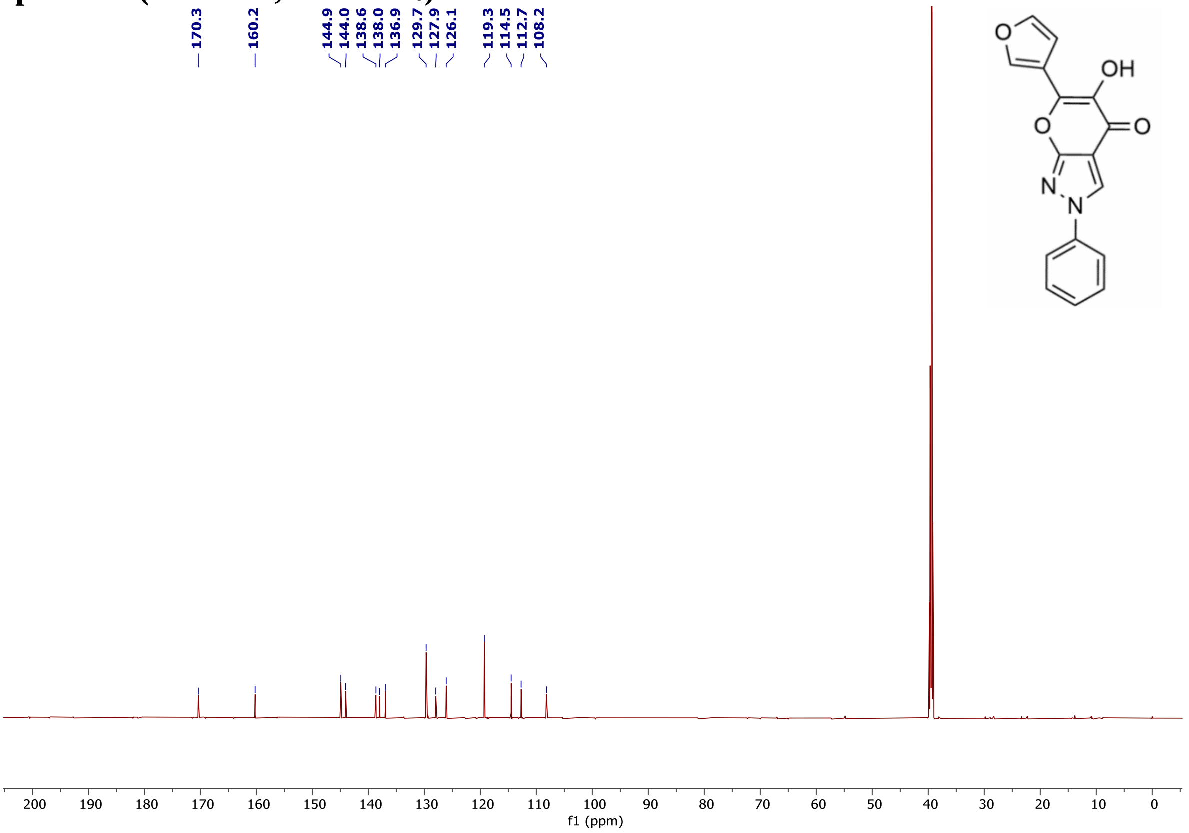

Figure S30. 6-(Furan-3-yl)-5-hydroxy-2-phenylpyrano[2,3-*c*]pyrazol-4(2*H*)-one 3g. ^{13}C NMR spectrum (176 MHz, DMSO-*d*$_6$)

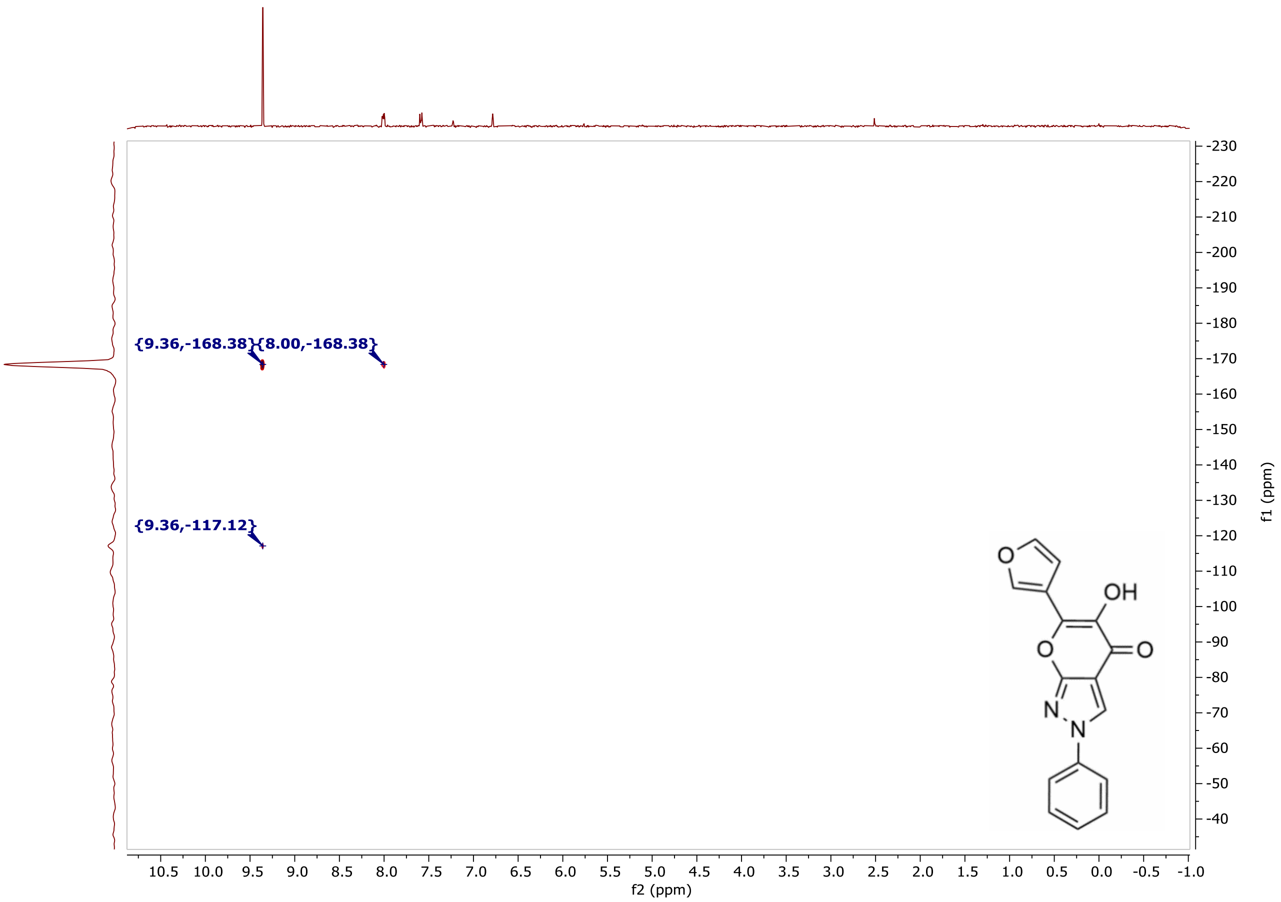

Figure S31. 6-(Furan-3-yl)-5-hydroxy-2-phenylpyrano[2,3-*c*]pyrazol-4(2*H*)-one 3g. ^{1}H-^{15}N HMBC NMR spectrum (71 MHz, DMSO-d_6)

Compound Spectrum SmartFormula Report

Analysis Info

Analysis Name	D:\Data\AUM-192.d	Acquisition Date	6/7/2023 7:56:52 PM
Method	DirectInfusion_TuneLow_pos.m	Operator	hplc
Sample Name	AUM-192	Instrument	micrOTOF-Q III 8228888.20448
Comment	AB		

Acquisition Parameter

Source Type	ESI	Ion Polarity	Positive	Set Nebulizer	0.4 Bar
Focus	Not active	Set Capillary	4500 V	Set Dry Heater	180 °C
Scan Begin	50 m/z	Set End Plate Offset	-500 V	Set Dry Gas	4.0 l/min
Scan End	1000 m/z	Set Collision Cell RF	140.0 Vpp	Set Divert Valve	Waste

#	RT [min]	Area	Int. Type	I	S/N	Chromatogram	Max. m/z	FWHM [min]
n.a.	7.0	n.a.	Single spectrum	n.a.	n.a.	n.a.	317.0534	n.a.

+MS, 7.0min #421

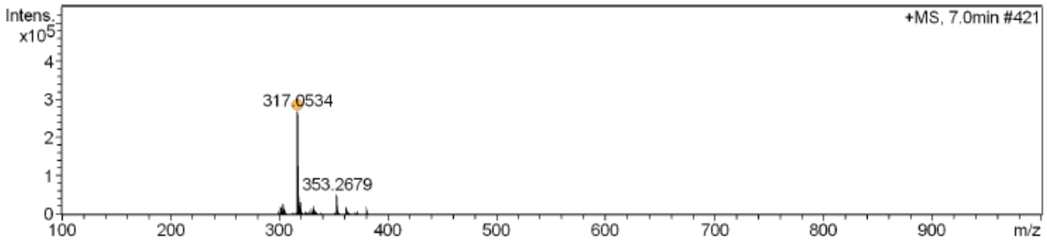

Meas. m/z	#	Ion Formula	m/z	err [ppm]	mSigma	# Sigma	Score	rdb	e¯ Conf	N-Rule
317.0534	1	C16H10N2NaO4	317.0533	0.4	5.1	1	100.00	12.5	even	ok

Figure S32. 6-(Furan-3-yl)-5-hydroxy-2-phenylpyrano[2,3-*c*]pyrazol-4(2*H*)-one 3g. HRMS (ESI)

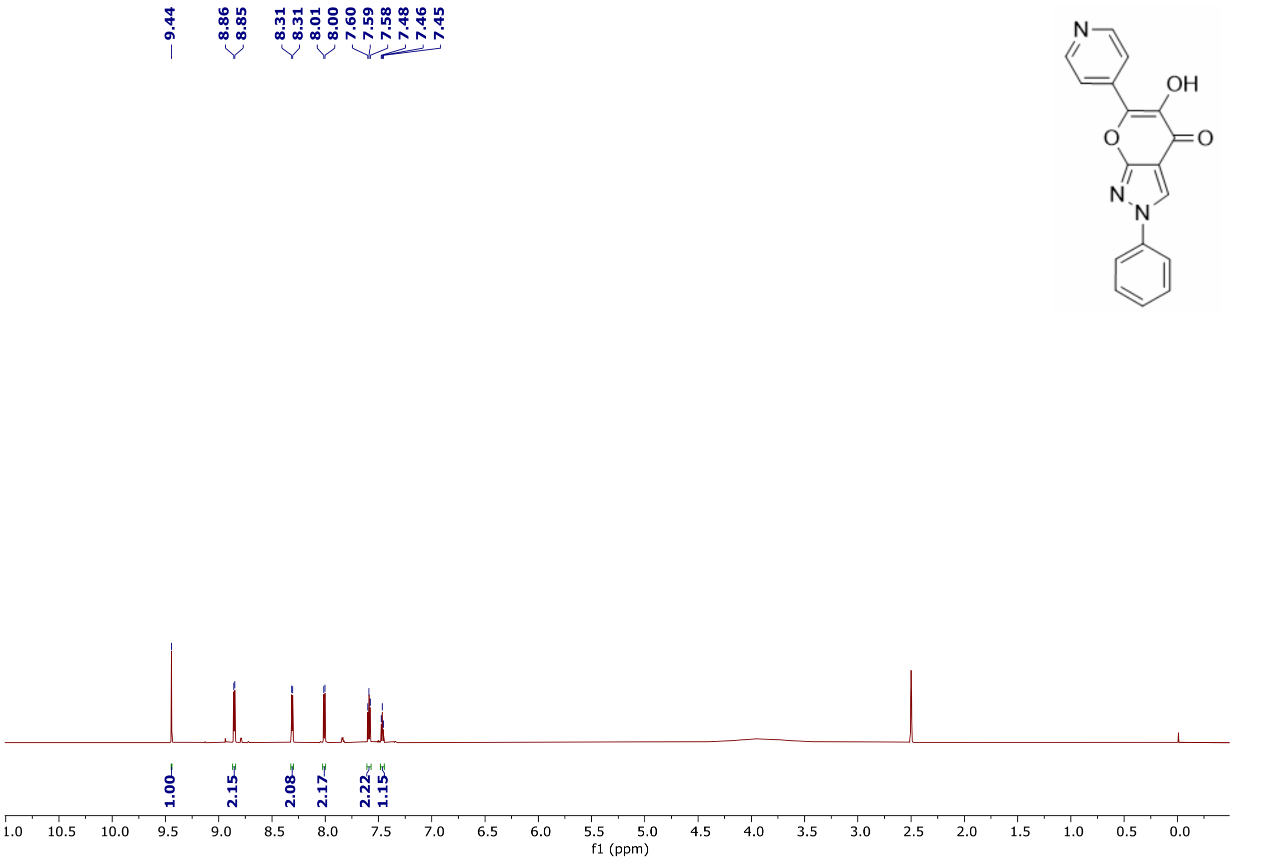

Figure S33. 5-Hydroxy-2-phenyl-6-(pyridin-4-yl)pyrano[2,3-*c*]pyrazol-4(2*H*)-one 3h. ^{1}H NMR spectrum (700 MHz, DMSO-*d*$_6$)

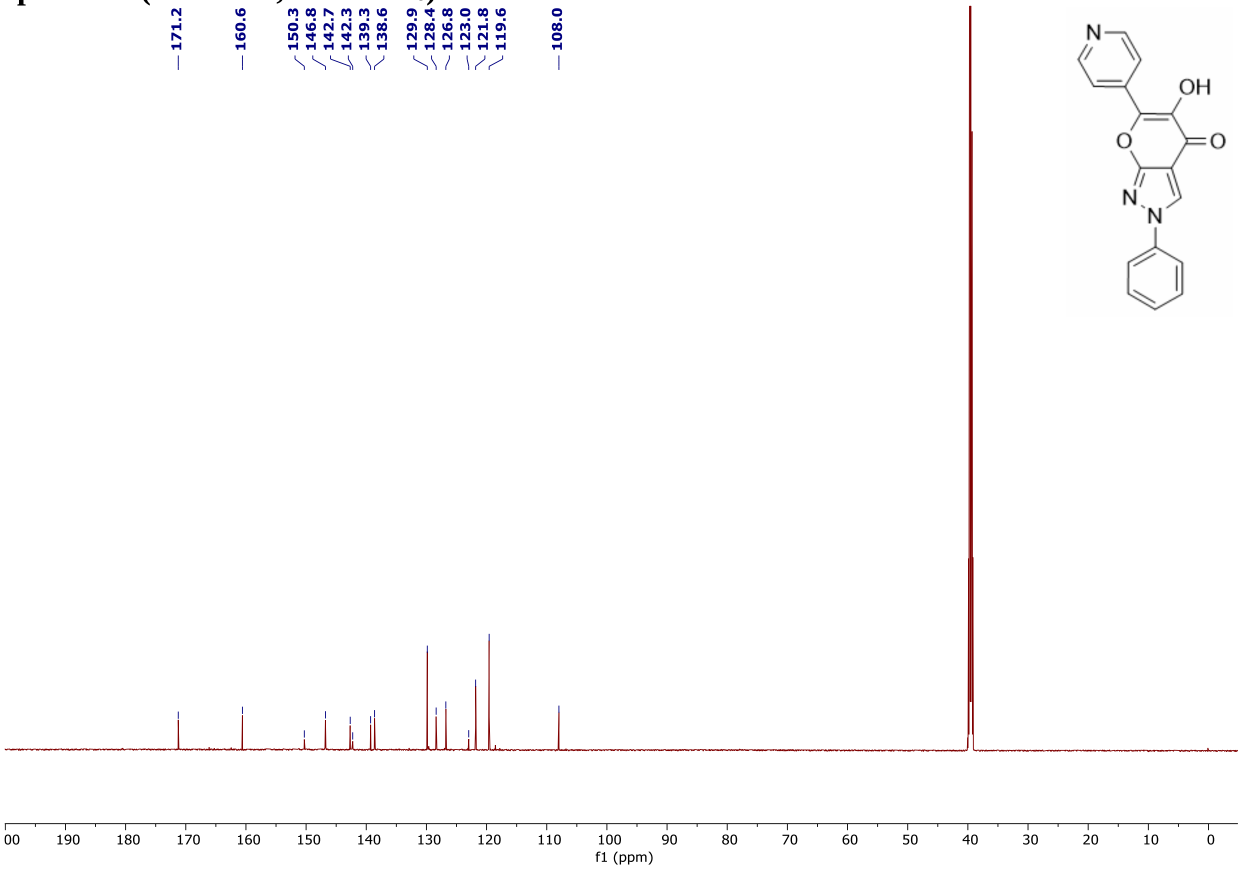

Figure S34. 5-Hydroxy-2-phenyl-6-(pyridin-4-yl)pyrano[2,3-*c*]pyrazol-4(2*H*)-one 3h. ^{13}C NMR spectrum (176 MHz, DMSO-*d*$_6$)

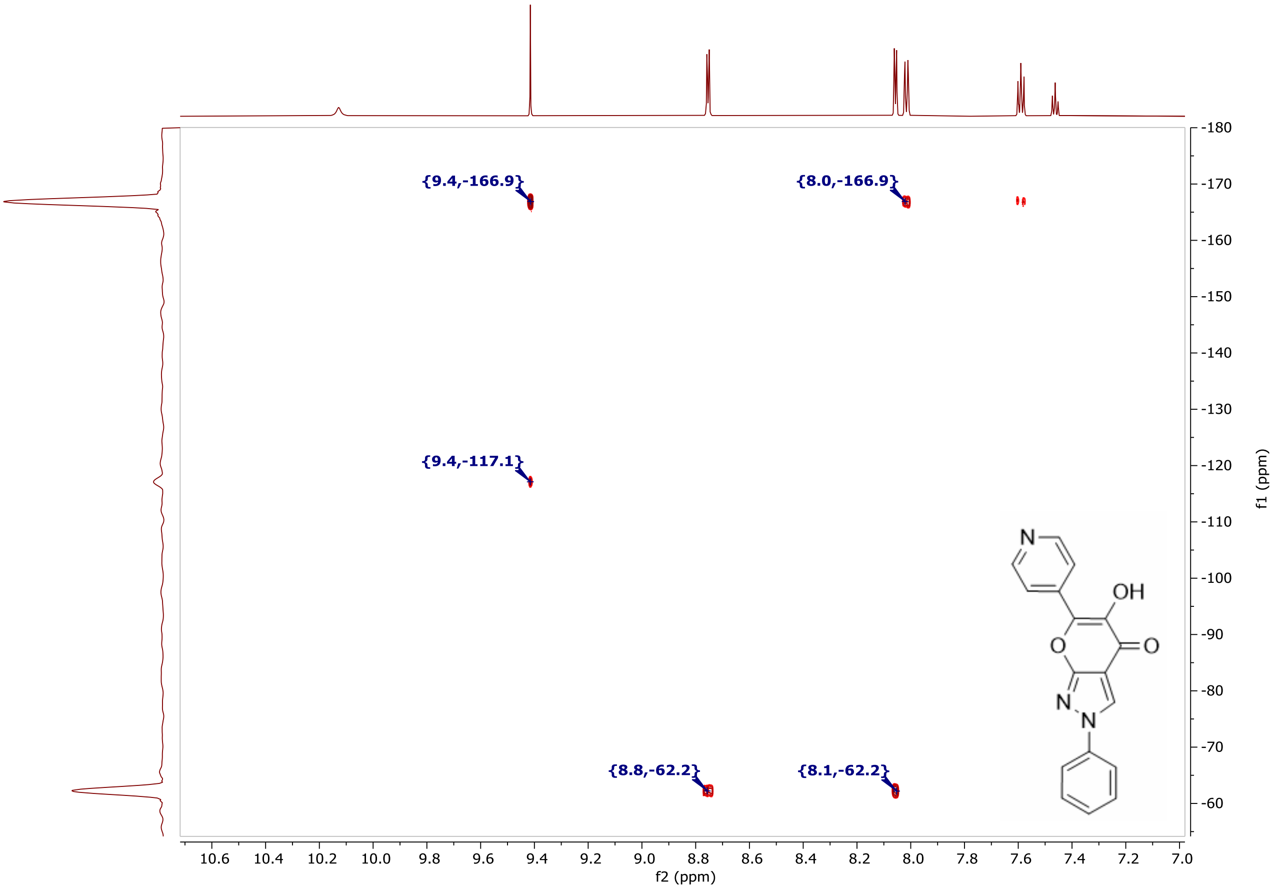

Figure S35. 5-Hydroxy-2-phenyl-6-(pyridin-4-yl)pyrano[2,3-*c*]pyrazol-4(2*H*)-one 3h. ^{1}H-^{15}N HMBC NMR spectrum (71 MHz, DMSO-d_6)

Compound Spectrum SmartFormula Report

Analysis Info

Analysis Name	D:\Data\AUM-208.d	Acquisition Date	8/4/2023 6:34:16 PM
Method	DirectInfusion_TuneLow_pos.m	Operator	hplc
Sample Name	AUM-208	Instrument	micrOTOF-Q III 8228888.20448
Comment	AB		

Acquisition Parameter

Source Type	ESI	Ion Polarity	Positive	Set Nebulizer	0.4 Bar
Focus	Not active	Set Capillary	4500 V	Set Dry Heater	180 °C
Scan Begin	50 m/z	Set End Plate Offset	-500 V	Set Dry Gas	4.0 l/min
Scan End	1000 m/z	Set Collision Cell RF	140.0 Vpp	Set Divert Valve	Waste

#	RT [min]	Area	Int. Type	I	S/N	Chromatogram	Max. m/z	FWHM [min]
n.a.	5.1	n.a.	Single spectrum	n.a.	n.a.	n.a.	306.0871	n.a.

+MS, 5.1min #303

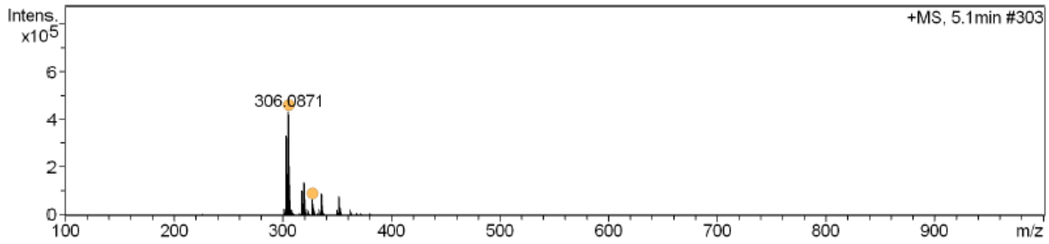

Meas. m/z	#	Ion Formula	m/z	err [ppm]	mSigma	# Sigma	Score	rdb	e⁻ Conf	N-Rule
306.0871	1	C17H12N3O3	306.0873	-0.7	58.6	1	100.00	13.5	even	ok
328.0687	1	C17H11N3NaO3	328.0693	1.6	3.8	1	100.00	13.5	even	ok

Figure S36. 5-Hydroxy-2-phenyl-6-(pyridin-4-yl)pyrano[2,3-*c*]pyrazol-4(2*H*)-one 3h. HRMS (ESI)

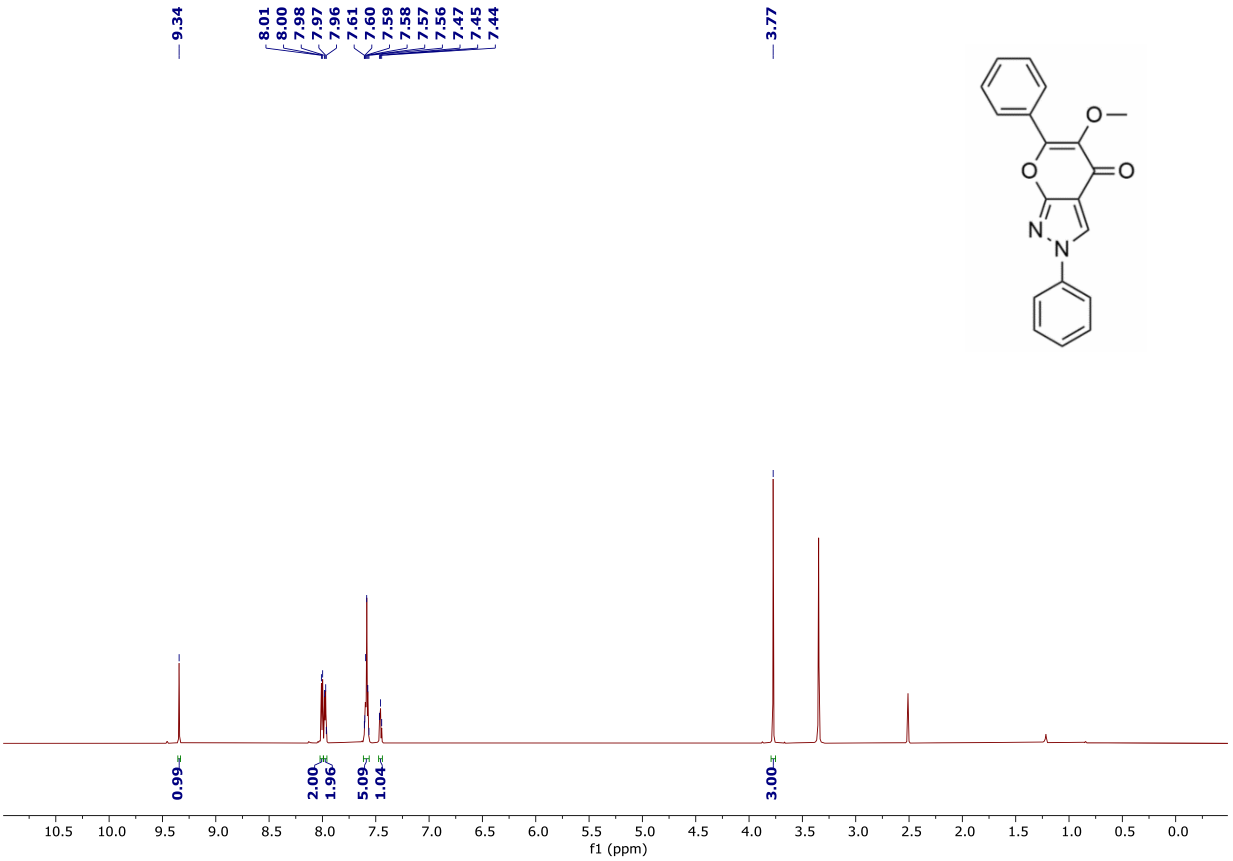

Figure S37. 5-Methoxy-2,6-diphenylpyrano[2,3-*c*]pyrazol-4(2*H*)-one 4. ^{1}H NMR spectrum (700 MHz, DMSO-d_6)

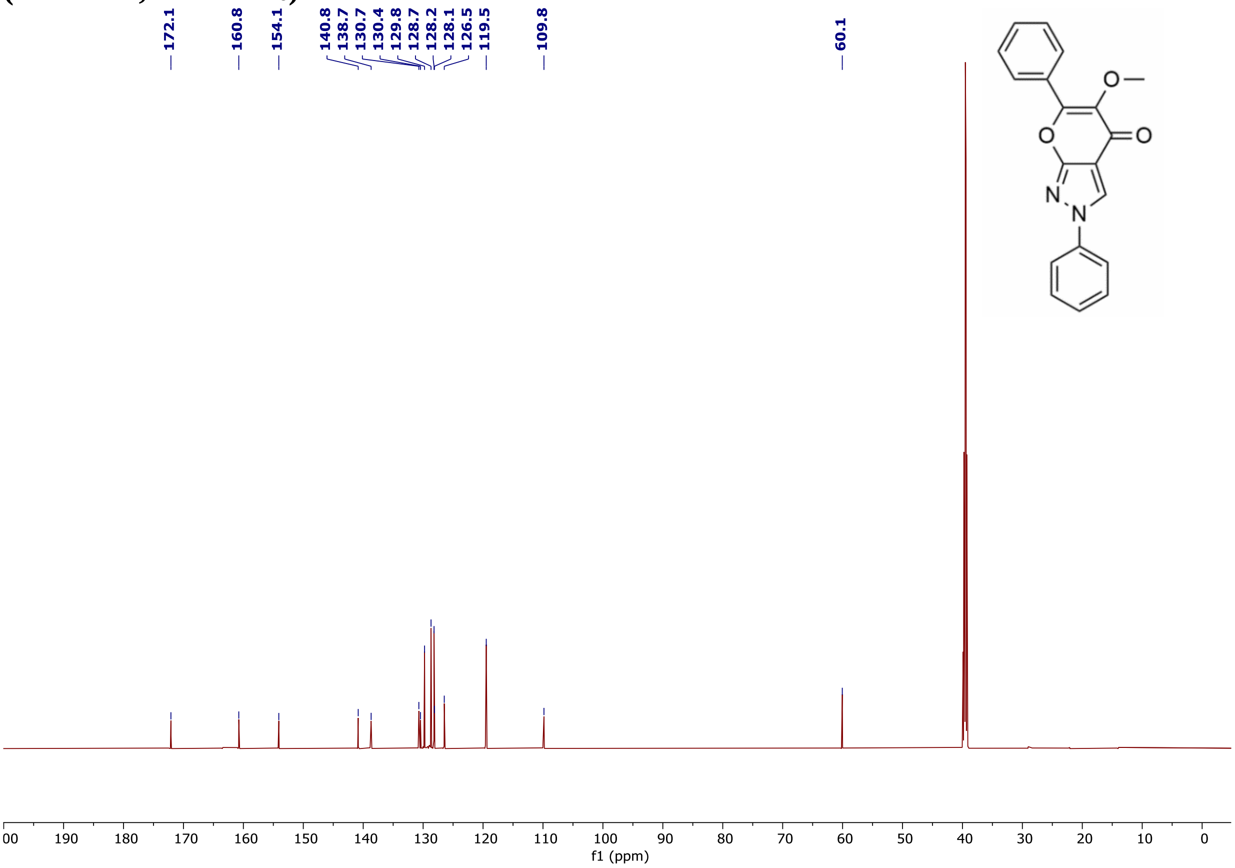

Figure S38. 5-Methoxy-2,6-diphenylpyrano[2,3-*c*]pyrazol-4(2*H*)-one 4. ^{13}C NMR spectrum (176 MHz, DMSO-d_6)

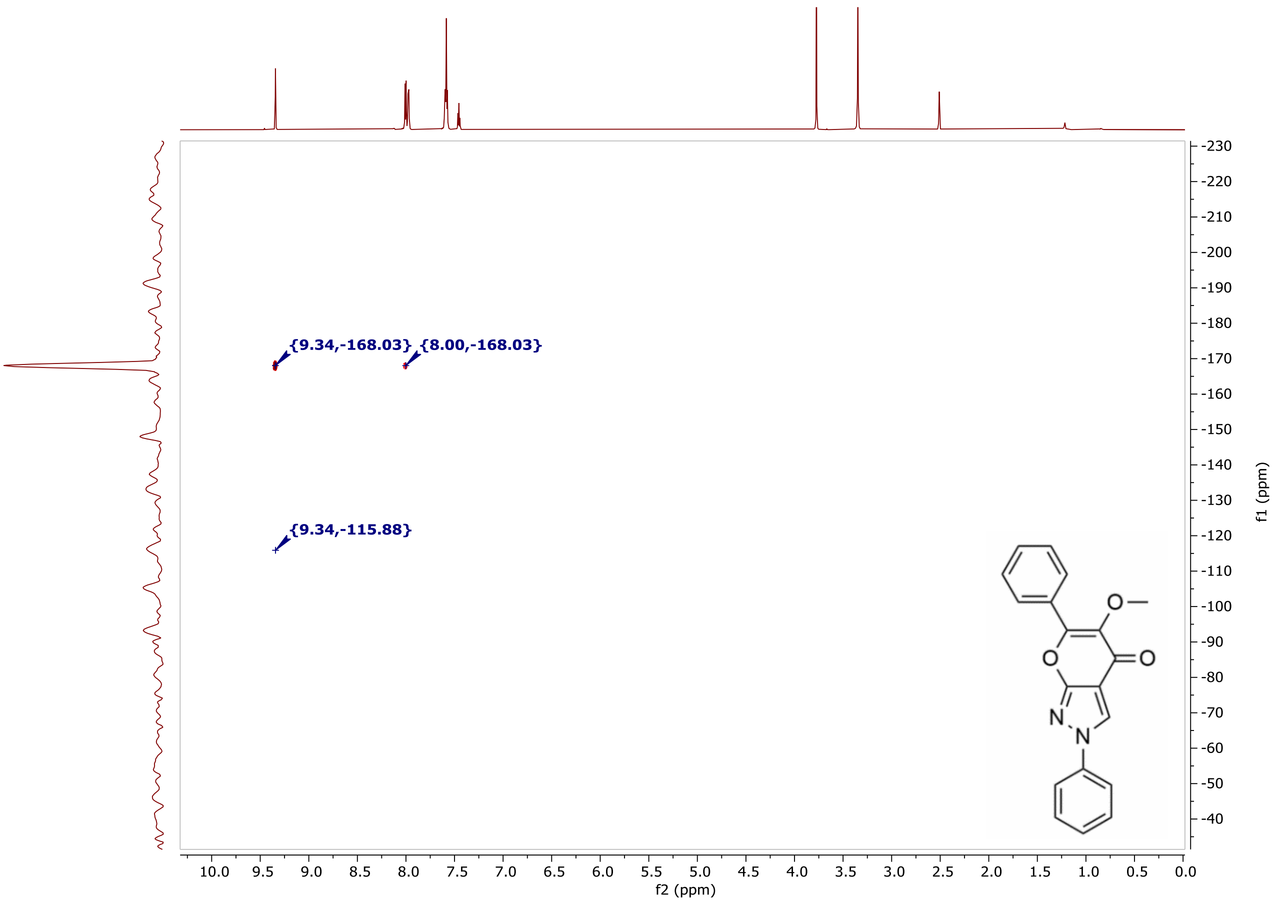

Figure S39. 5-Methoxy-2,6-diphenylpyrano[2,3-*c*]pyrazol-4(2*H*)-one 4. ^{1}H-^{15}N HMBC NMR spectrum (71 MHz, DMSO-*d*$_6$)

Compound Spectrum SmartFormula Report

Analysis Info

Analysis Name	D:\Data\AUM-212.d	Acquisition Date	6/7/2023 6:45:26 PM
Method	DirectInfusion_TuneLow_pos.m	Operator	hplc
Sample Name	AUM-212	Instrument	micrOTOF-Q III 8228888.20448
Comment	AB		

Acquisition Parameter

Source Type	ESI	Ion Polarity	Positive	Set Nebulizer	0.4 Bar
Focus	Not active	Set Capillary	4500 V	Set Dry Heater	180 °C
Scan Begin	50 m/z	Set End Plate Offset	-500 V	Set Dry Gas	4.0 l/min
Scan End	1000 m/z	Set Collision Cell RF	140.0 Vpp	Set Divert Valve	Waste

#	RT [min]	Area	Int. Type	I	S/N	Chromatogram	Max. m/z	FWHM [min]
n.a.	6.4	n.a.	Single spectrum	n.a.	n.a.	n.a.	341.0899	n.a.

+MS, 6.4min #385

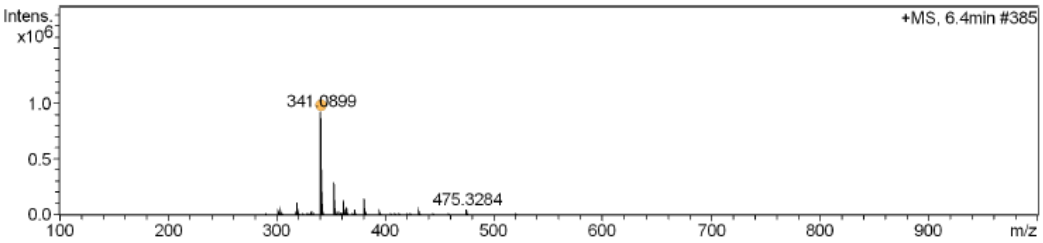

Meas. m/z	#	Ion Formula	m/z	err [ppm]	mSigma	# Sigma	Score	rdb	e⁻ Conf	N-Rule
341.0899	1	C19H14N2NaO3	341.0897	-0.7	13.6	2	100.00	13.5	even	ok

Figure S40. 5-Methoxy-2,6-diphenylpyrano[2,3-*c*]pyrazol-4(2*H*)-one 4. HRMS (ESI)

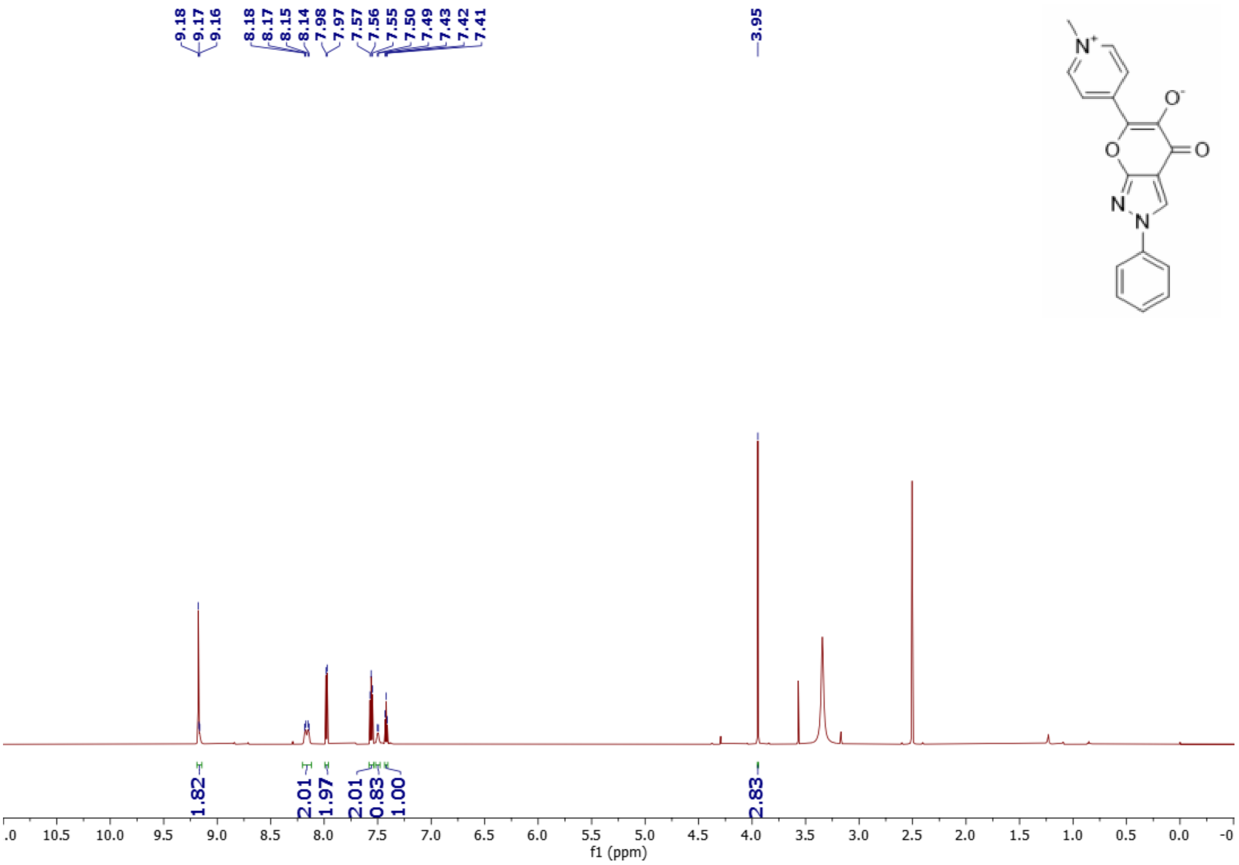

Figure S41. 6-(1-Methylpyridin-4(1*H*)-ylidene)-2-phenylpyrano[2,3-*c*]pyrazole-4,5(2*H*,6*H*)-dione 5. ^{1}H NMR spectrum (700 MHz, DMSO-*d*$_6$)

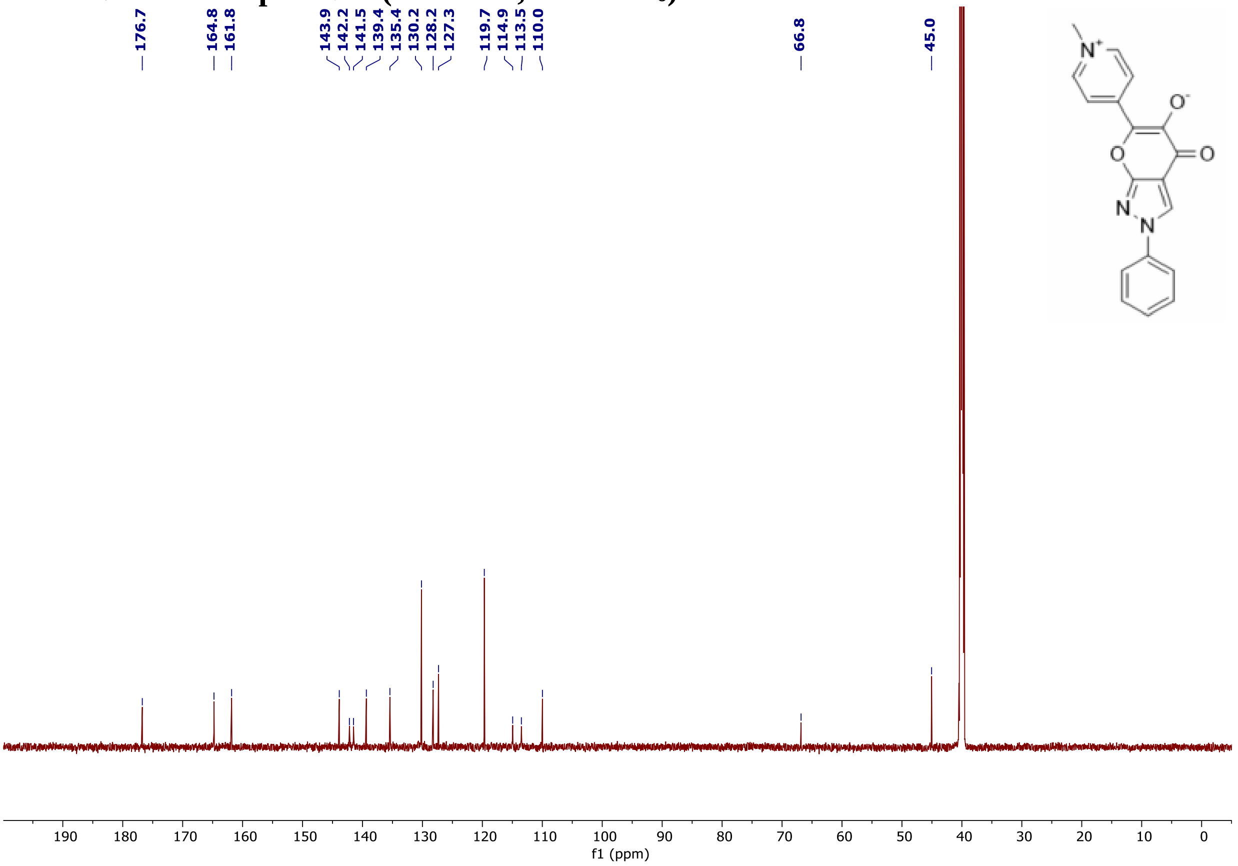

Figure S42. 6-(1-methylpyridin-4(1*H*)-ylidene)-2-phenylpyrano[2,3-*c*]pyrazole-4,5(2*H*,6*H*)-dione 5. ^{13}C NMR spectrum (176 MHz, DMSO-*d*$_6$)

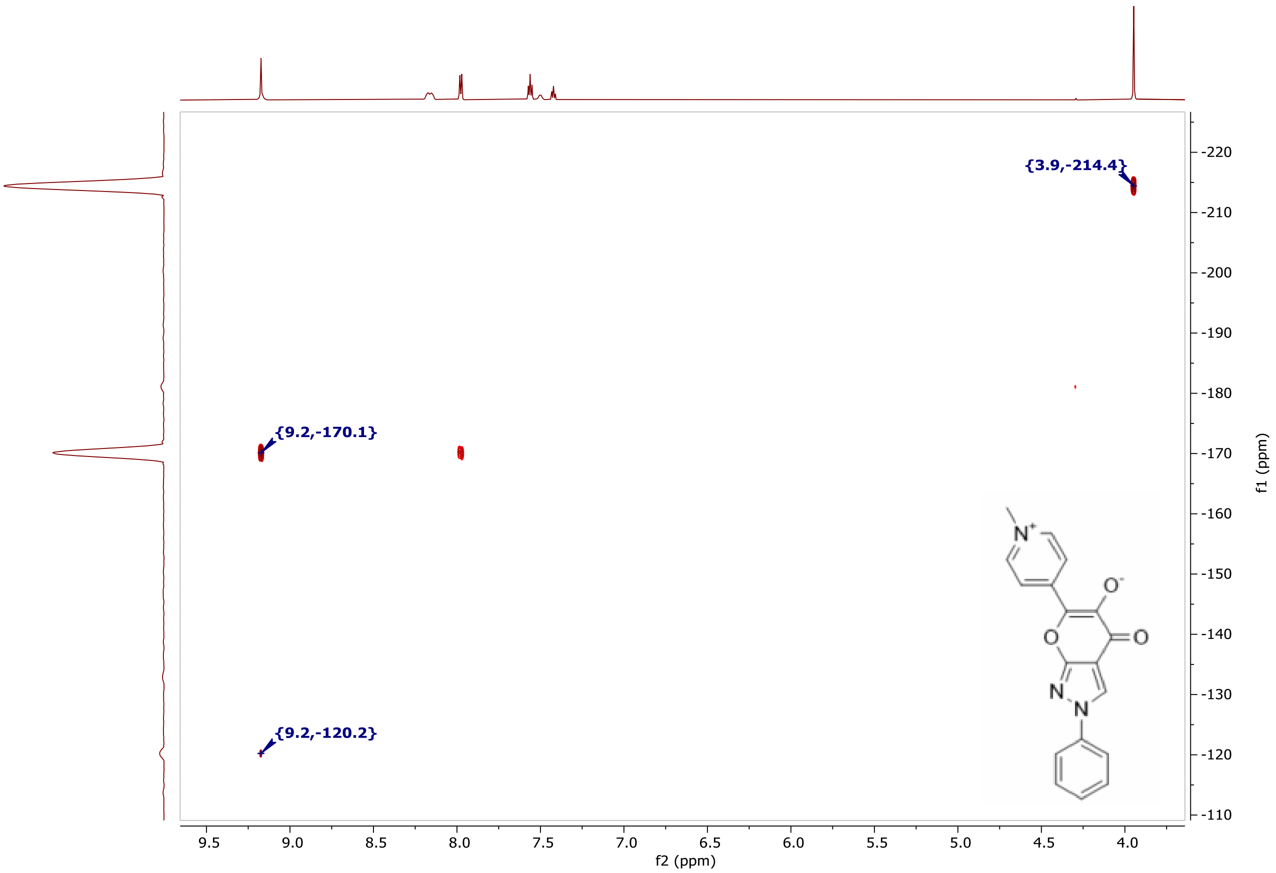

Figure S43. 6-(1-Methylpyridin-4(1*H*)-ylidene)-2-phenylpyrano[2,3-*c*]pyrazole-4,5(2*H*,6*H*)-dione 5. ^{1}H-^{15}N HMBC NMR spectrum (71 MHz, DMSO-*d*$_6$)

Compound Spectrum SmartFormula Report

Analysis Info

Analysis Name	D:\Data\AUM-215_20230314.d	Acquisition Date	3/14/2023 4:01:47 PM
Method	DirectInfusion_TuneLow_pos.m	Operator	hplc
Sample Name	AUM-215_20230314	Instrument	micrOTOF-Q III 8228888.20448
Comment	AB		

Acquisition Parameter

Source Type	ESI	Ion Polarity	Positive	Set Nebulizer	0.4 Bar
Focus	Not active	Set Capillary	4500 V	Set Dry Heater	180 °C
Scan Begin	50 m/z	Set End Plate Offset	-500 V	Set Dry Gas	4.0 l/min
Scan End	1000 m/z	Set Collision Cell RF	140.0 Vpp	Set Divert Valve	Waste

#	RT [min]	Area	Int. Type	I	S/N	Chromatogram	Max. m/z	FWHM [min]
n.a.	14.5	n.a.	Single spectrum	n.a.	n.a.	n.a.	428.1660	n.a.

+MS, 14.5min #871

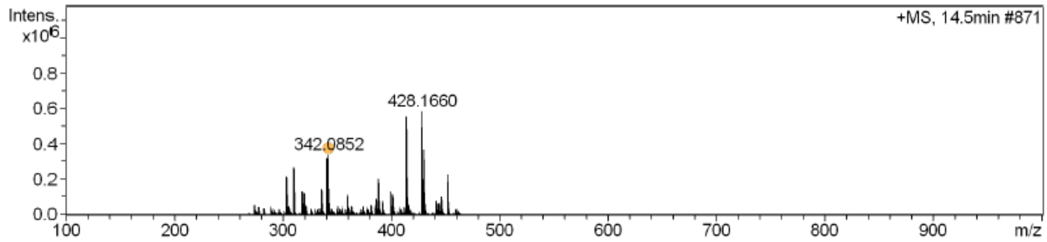

Meas. m/z	#	Ion Formula	m/z	err [ppm]	mSigma	# Sigma	Score	rdb	e⁻ Conf	N-Rule
342.0852	1	C18H13N3NaO3	342.0849	0.9	16.0	1	100.00	13.5	even	ok

Figure S44. 6-(1-Methylpyridin-4(1*H*)-ylidene)-2-phenylpyrano[2,3-*c*]pyrazole-4,5(2*H*,6*H*)-dione 5. HRMS (ESI)

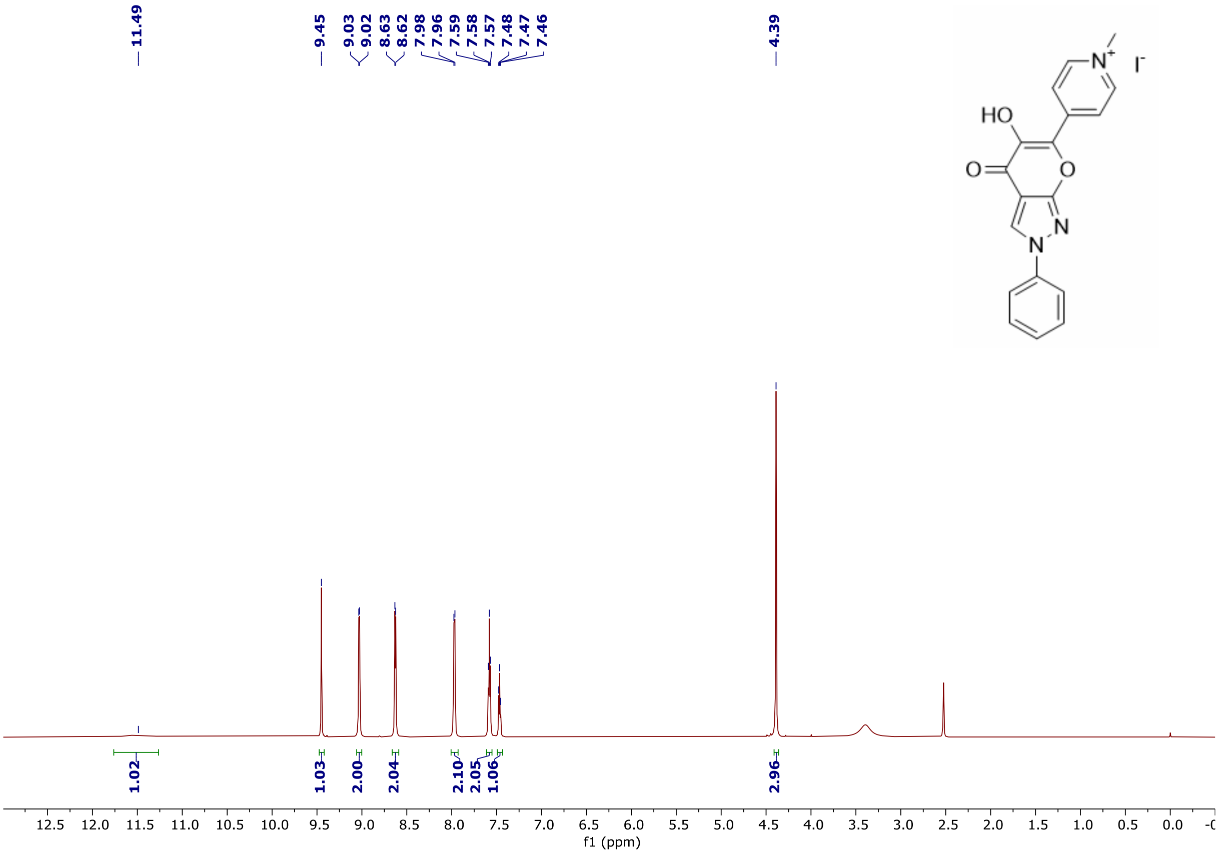

Figure S45. 4-(5-Hydroxy-4-oxo-2-phenyl-2,4-dihydropyrano[2,3-*c*]pyrazol-6-yl)-1-methylpyridin-1-ium iodide 6 ^{1}H NMR spectrum (700 MHz, DMSO-d_6)

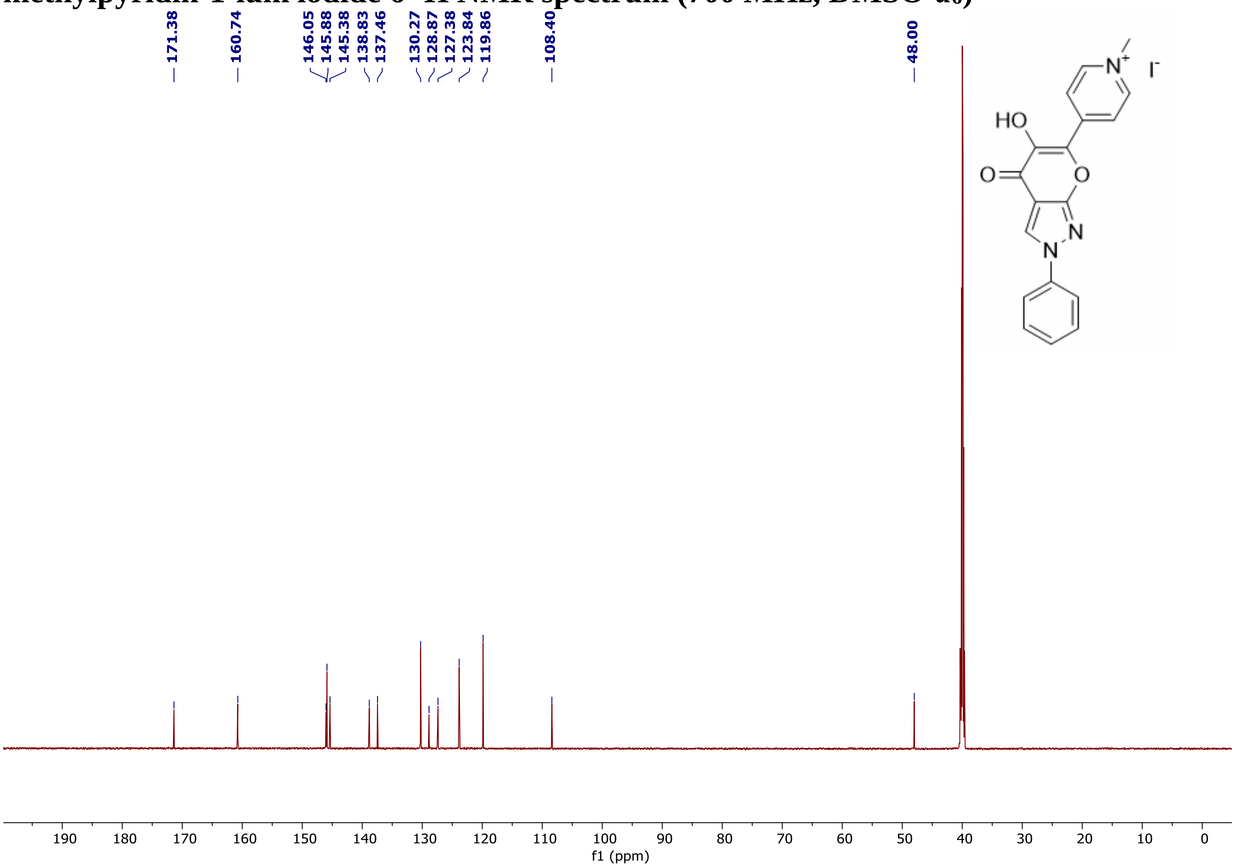

Figure S46. 4-(5-Hydroxy-4-oxo-2-phenyl-2,4-dihydropyrano[2,3-*c*]pyrazol-6-yl)-1-methylpyridin-1-ium iodide 6 ^{13}C NMR spectrum (176 MHz, DMSO-d_6)

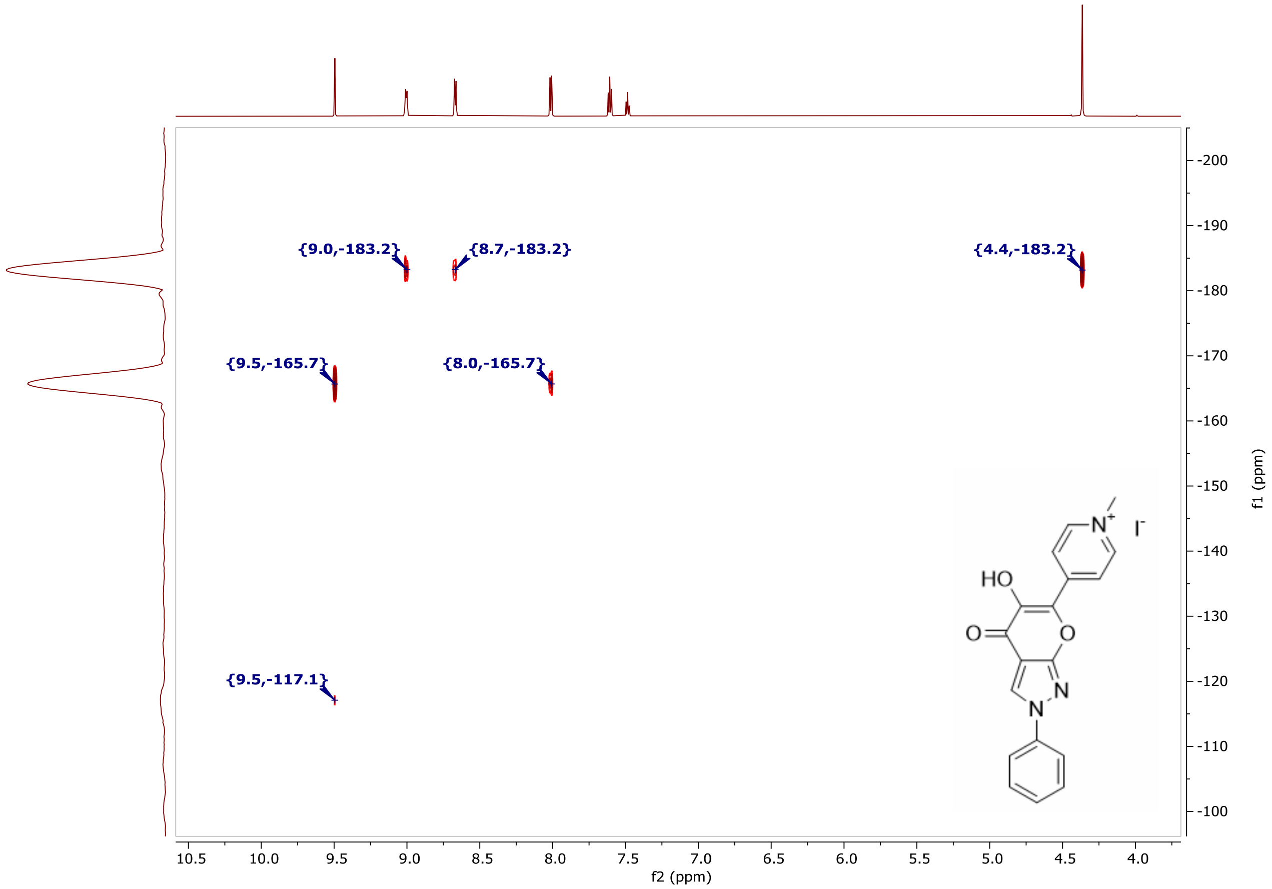

Figure S47. **4-(5-Hydroxy-4-oxo-2-phenyl-2,4-dihydropyrano[2,3-*c*]pyrazol-6-yl)-1-methylpyridin-1-ium iodide 6 ^{1}H-^{15}N HMBC NMR spectrum (71 MHz, DMSO-d_6)**

Compound Spectrum SmartFormula Report

Analysis Info

Analysis Name	D:\Data\AUM-241-K.d	Acquisition Date	8/4/2023 7:06:20 PM
Method	DirectInfusion_TuneLow_pos.m	Operator	hplc
Sample Name	AUM-241-K	Instrument	micrOTOF-Q III 8228888.20448
Comment	AB		

Acquisition Parameter

Source Type	ESI	Ion Polarity	Positive	Set Nebulizer	0.4 Bar
Focus	Not active	Set Capillary	4500 V	Set Dry Heater	180 °C
Scan Begin	50 m/z	Set End Plate Offset	-500 V	Set Dry Gas	4.0 l/min
Scan End	1000 m/z	Set Collision Cell RF	140.0 Vpp	Set Divert Valve	Waste

#	RT [min]	Area	Int. Type	I	S/N	Chromatogram	Max. m/z	FWHM [min]
n.a.	3.7	n.a.	Single spectrum	n.a.	n.a.	n.a.	320.1032	n.a.

+MS, 3.7min #223

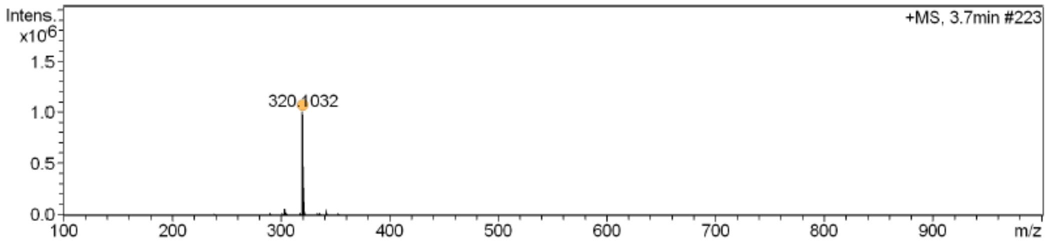

Meas. m/z	#	Ion Formula	m/z	err [ppm]	mSigma	# Sigma	Score	rdb	e⁻ Conf	N-Rule
320.1032	1	C18H14N3O3	320.1030	-0.6	36.3	2	100.00	13.5	even	ok

Figure S48. 4-(5-Hydroxy-4-oxo-2-phenyl-2,4-dihydropyrano[2,3-*c*]pyrazol-6-yl)-1-methylpyridin-1-ium iodide 6. HRMS (ESI)

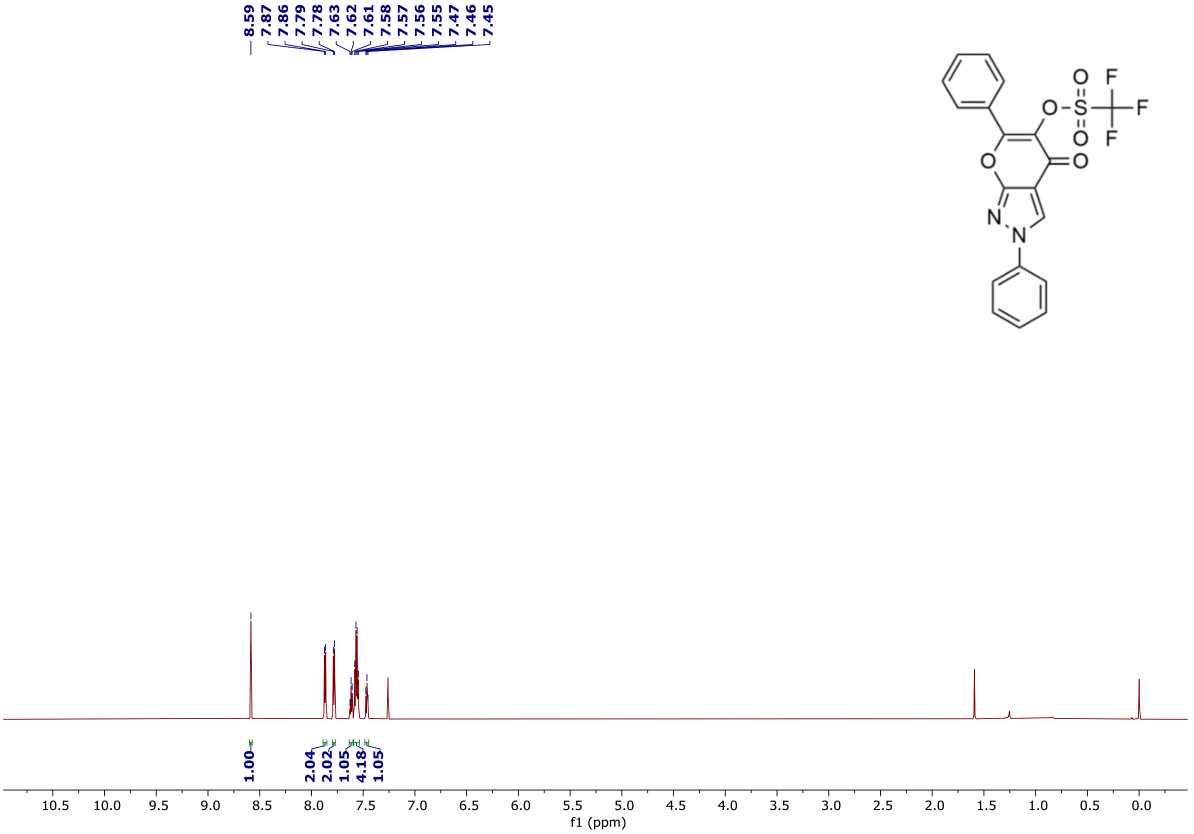

Figure S49. 4-Oxo-2,6-diphenyl-2,4-dihydropyrano[2,3-*c*]pyrazol-5-yl trifluoromethanesulfonate 7. ^{1}H NMR spectrum (700 MHz, $CDCl_3$)

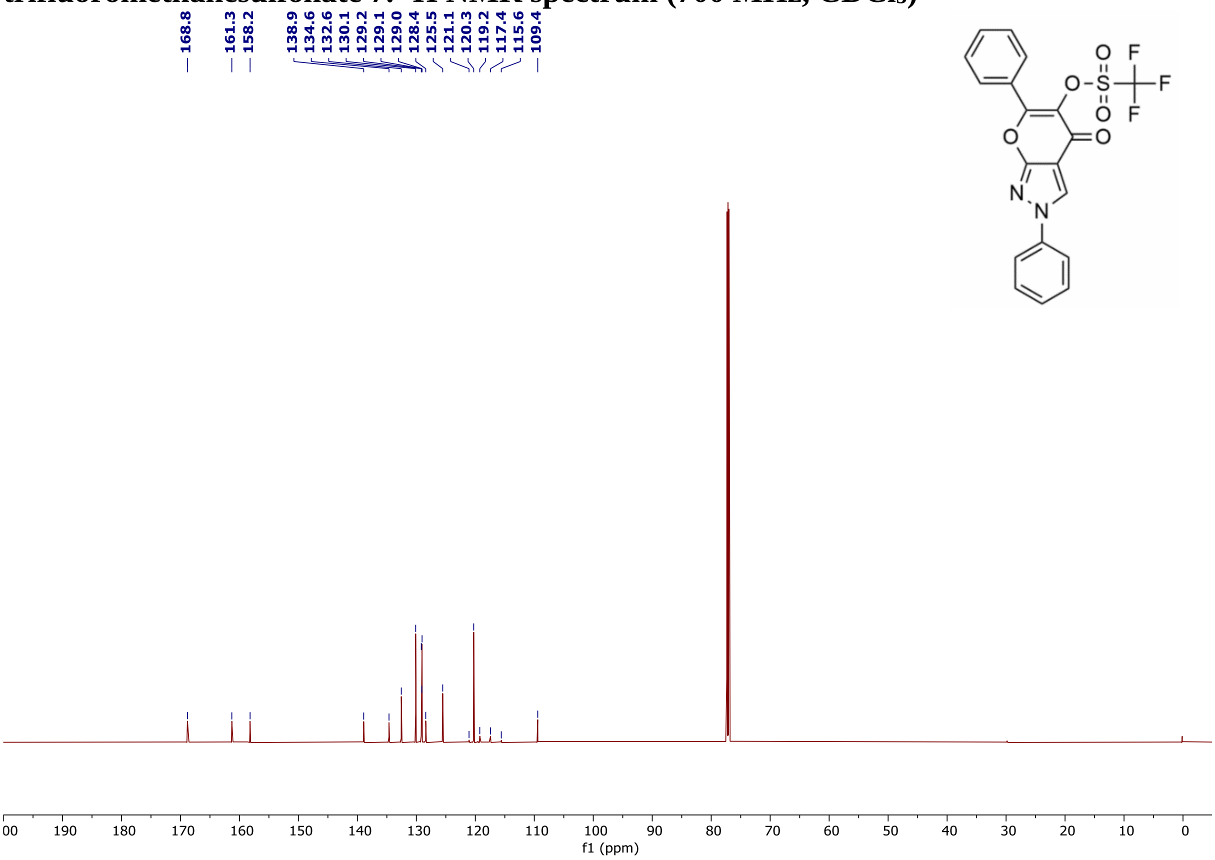

Figure S50. 4-Oxo-2,6-diphenyl-2,4-dihydropyrano[2,3-*c*]pyrazol-5-yl trifluoromethanesulfonate 7. ^{13}C NMR spectrum (176 MHz, $CDCl_3$)

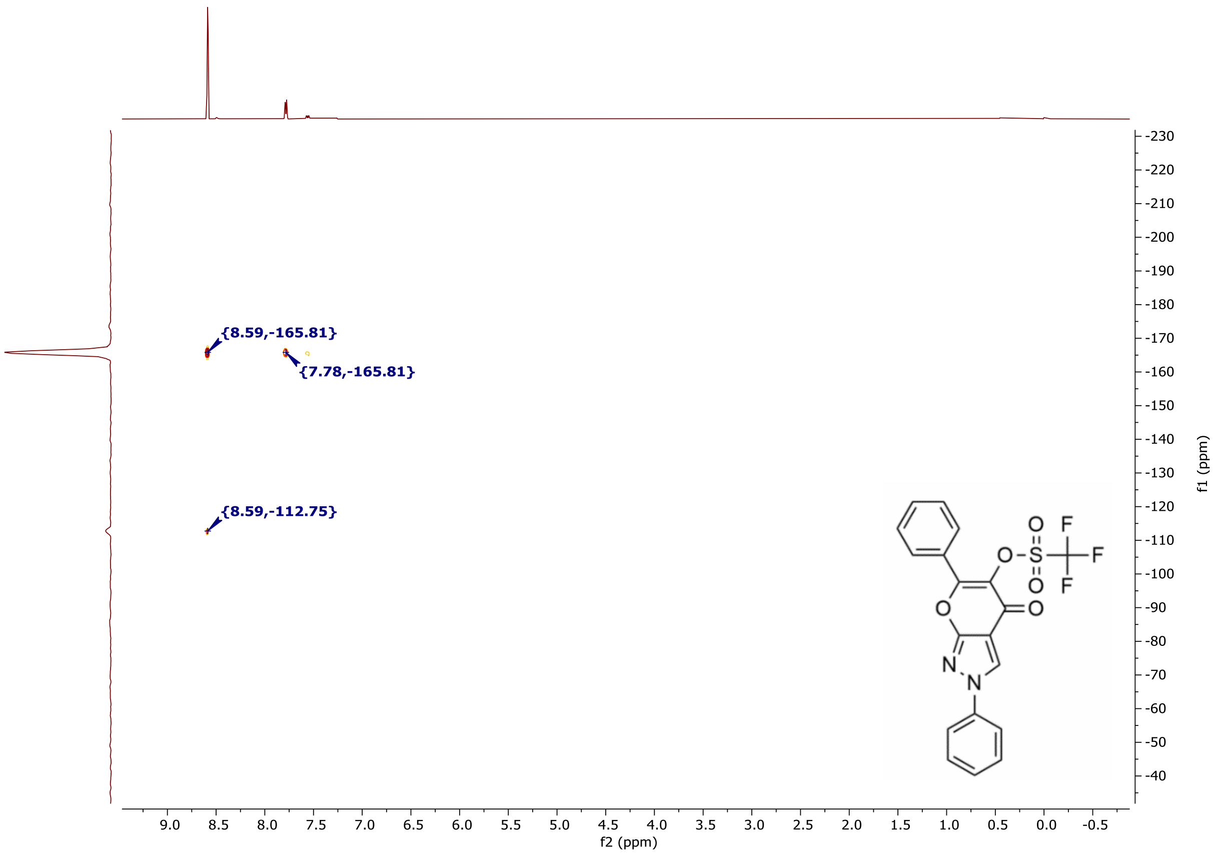

Figure S51. 4-Oxo-2,6-diphenyl-2,4-dihydropyrano[2,3-*c*]pyrazol-5-yl trifluoromethanesulfonate 7. ^{1}H-^{15}N HMBC NMR spectrum (71 MHz, $CDCl_3$)

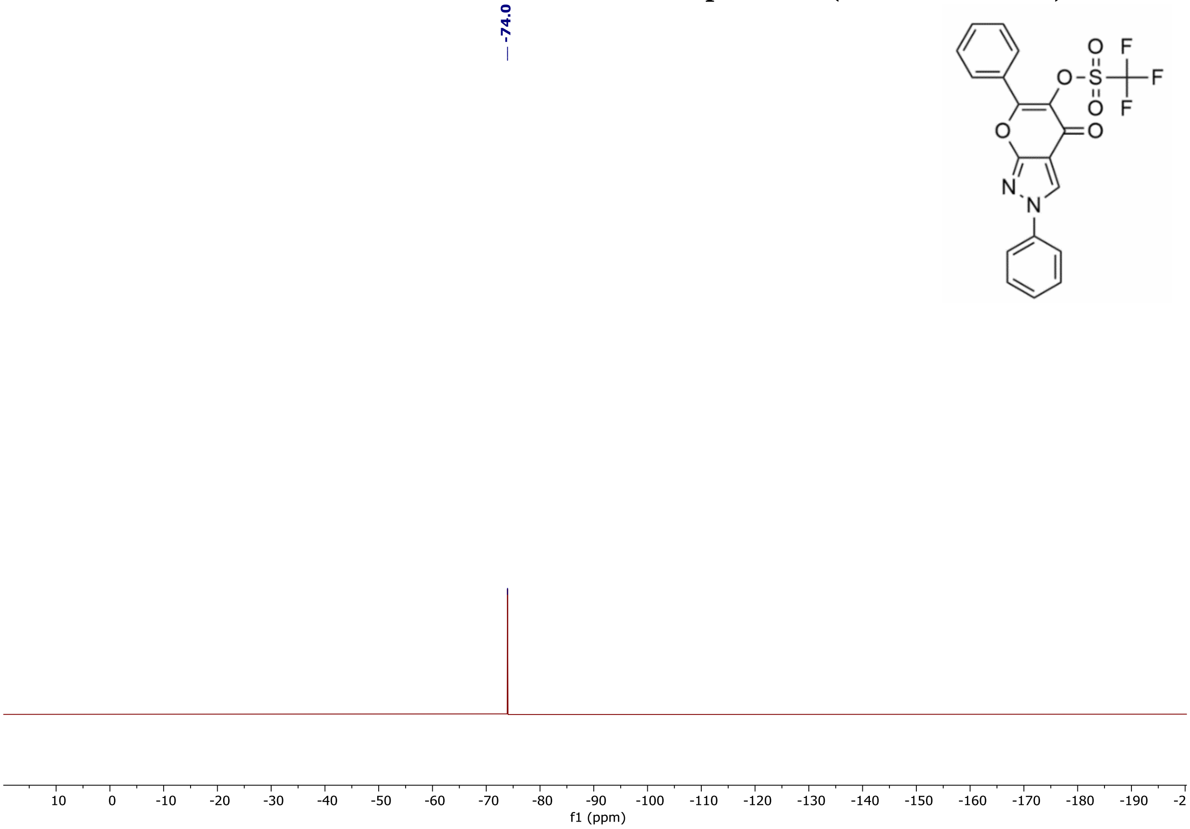

Figure S52. 4-Oxo-2,6-diphenyl-2,4-dihydropyrano[2,3-*c*]pyrazol-5-yl trifluoromethanesulfonate 7. ^{19}F NMR (376 MHz, $CDCl_3$)

Compound Spectrum SmartFormula Report

Analysis Info

Analysis Name	D:\Data\AUM-220.d	Acquisition Date	6/7/2023 6:30:06 PM
Method	DirectInfusion_TuneLow_pos.m	Operator	hplc
Sample Name	AUM-220	Instrument	microTOF-Q III 8228888.20448
Comment	AB		

Acquisition Parameter

Source Type	ESI	Ion Polarity	Positive	Set Nebulizer	0.4 Bar
Focus	Not active	Set Capillary	4500 V	Set Dry Heater	180 °C
Scan Begin	50 m/z	Set End Plate Offset	-500 V	Set Dry Gas	4.0 l/min
Scan End	1000 m/z	Set Collision Cell RF	140.0 Vpp	Set Divert Valve	Waste

#	RT [min]	Area	Int. Type	I	S/N	Chromatogram	Max. m/z	FWHM [min]
n.a.	7.8	n.a.	Single spectrum	n.a.	n.a.	n.a.	459.0235	n.a.

+MS, 7.8min #468

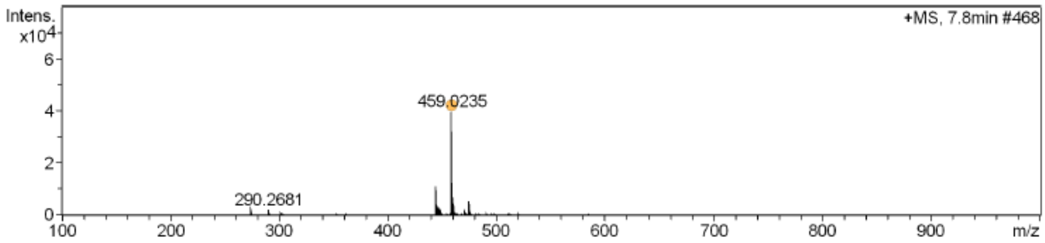

Meas. m/z	#	Ion Formula	m/z	err [ppm]	mSigma	# Sigma	Score	rdb	e¯ Conf	N-Rule
459.0235	1	C19H11F3N2NaO5S	459.0233	-0.5	19.5	4	100.00	13.5	even	ok

Figure S53. 4-Oxo-2,6-diphenyl-2,4-dihydropyrano[2,3-*c*]pyrazol-5-yl trifluoromethanesulfonate 7. HRMS (ESI)

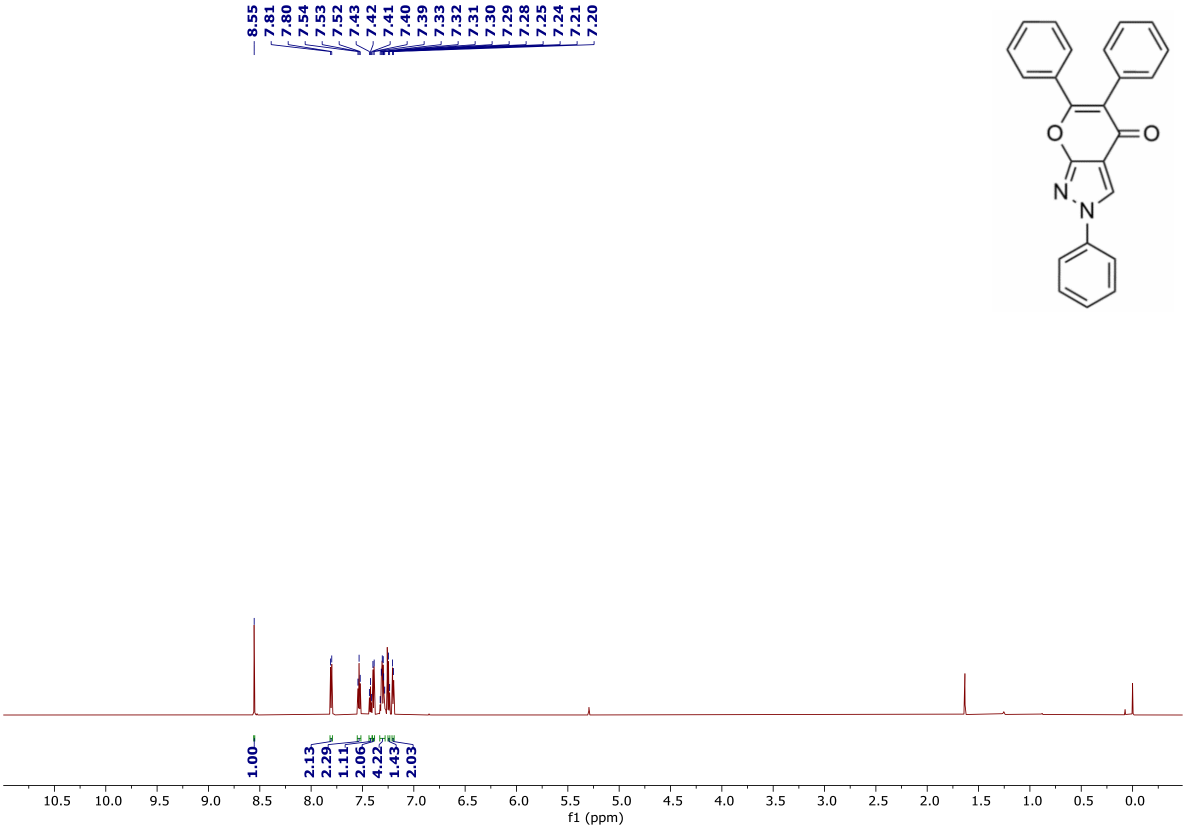

Figure S54. 2,5,6-Triphenylpyrano[2,3-*c*]pyrazol-4(2*H*)-one 8a. ^{1}H NMR spectrum (700 MHz, CDCl$_3$)

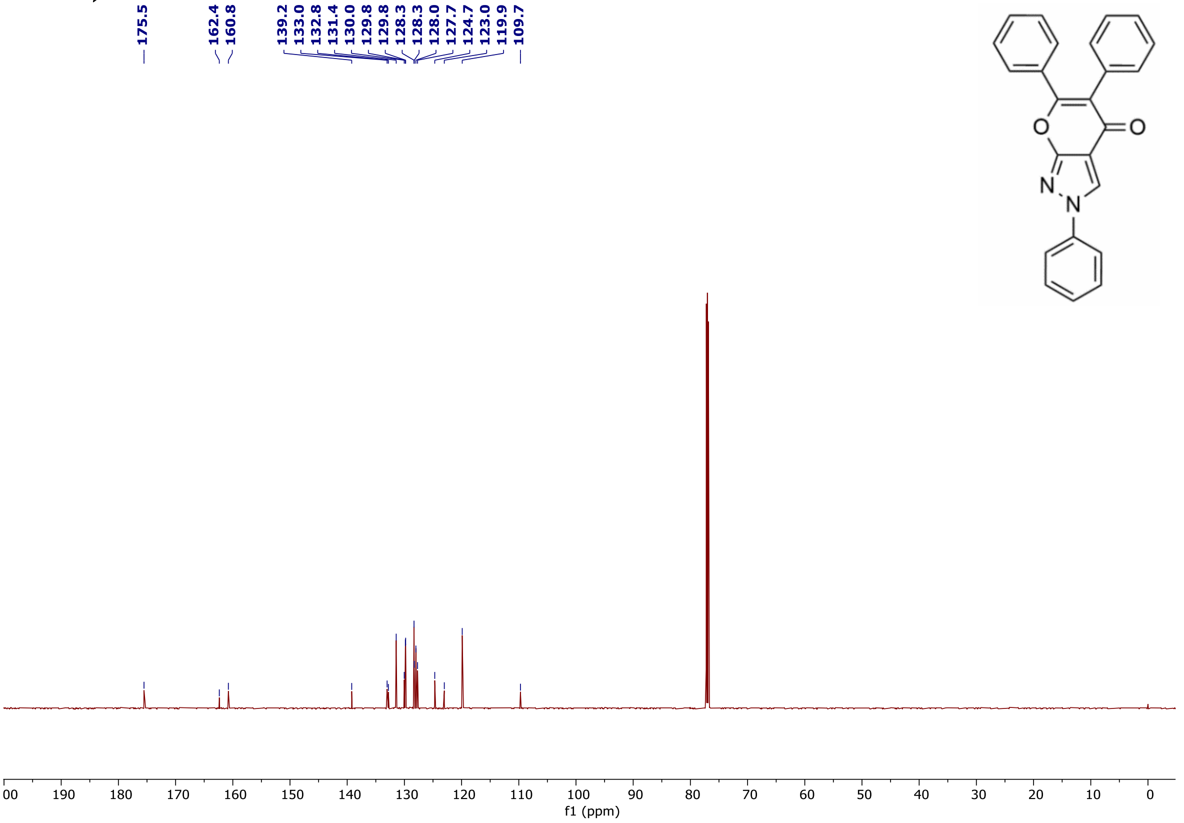

Figure S55. 2,5,6-Triphenylpyrano[2,3-*c*]pyrazol-4(2*H*)-one 8a. ^{13}C NMR spectrum (176 MHz, CDCl$_3$)

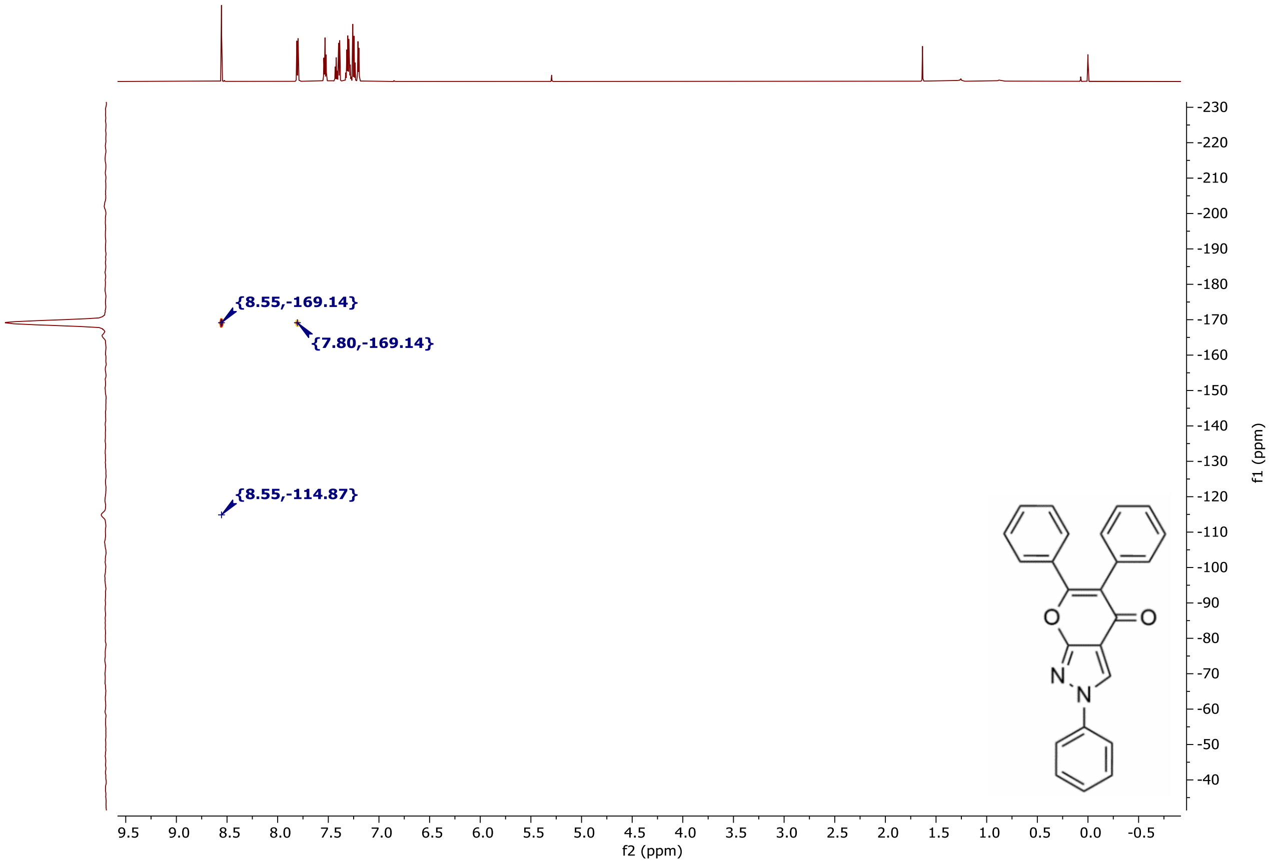

Figure S56. 2,5,6-Triphenylpyrano[2,3-*c*]pyrazol-4(2*H*)-one 8a. ^{1}H-^{15}N HMBC NMR spectrum (71 MHz, $CDCl_3$)

Compound Spectrum SmartFormula Report

Analysis Info

Analysis Name	D:\Data\AUM-222.d	Acquisition Date	6/7/2023 4:23:28 PM
Method	DirectInfusion_TuneLow_pos.m	Operator	hplc
Sample Name	AUM-222	Instrument	micrOTOF-Q III 8228888.20448
Comment	AB		

Acquisition Parameter

Source Type	ESI	Ion Polarity	Positive	Set Nebulizer	0.4 Bar
Focus	Not active	Set Capillary	4500 V	Set Dry Heater	180 °C
Scan Begin	50 m/z	Set End Plate Offset	-500 V	Set Dry Gas	4.0 l/min
Scan End	1000 m/z	Set Collision Cell RF	140.0 Vpp	Set Divert Valve	Waste

#	RT [min]	Area	Int. Type	I	S/N	Chromatogram	Max. m/z	FWHM [min]
n.a.	6.3	n.a.	Single spectrum	n.a.	n.a.	n.a.	387.1107	n.a.

+MS, 6.3min #375

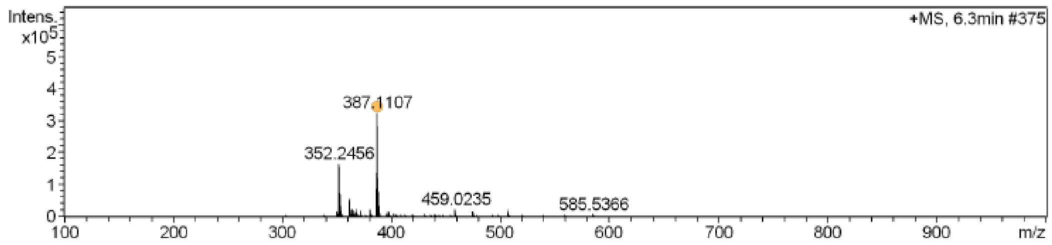

Meas. m/z	#	Ion Formula	m/z	err [ppm]	mSigma	# Sigma	Score	rdb	e⁻ Conf	N-Rule
387.1107	1	C24H16N2NaO2	387.1104	0.7	2.5	1	100.00	17.5	even	ok

Figure S57. 2,5,6-Triphenylpyrano[2,3-*c*]pyrazol-4(2*H*)-one 8a. HRMS (ESI)

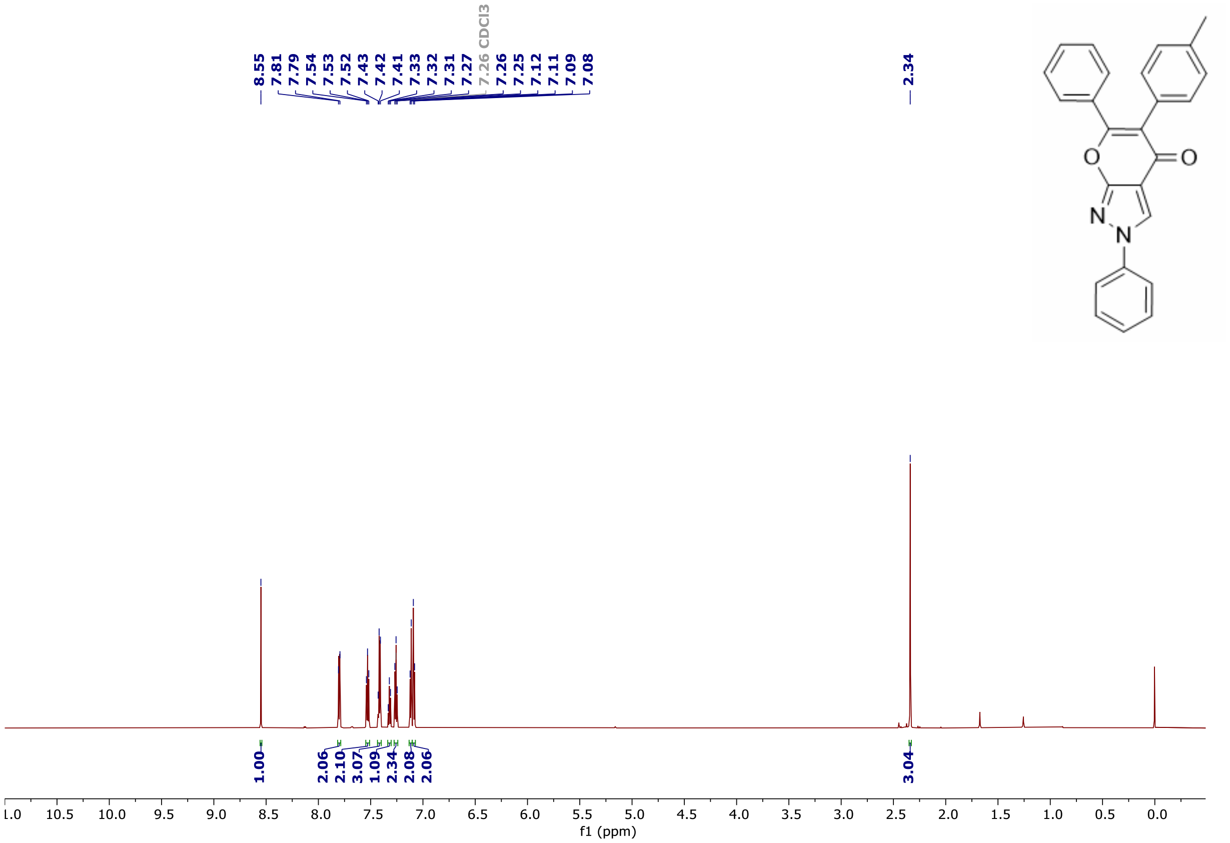

Figure S58. 5-(4-Methylphenyl)-2,6-diphenylpyrano[2,3-*c*]pyrazol-4(2*H*)-one 8b. ^{1}H NMR spectrum (700 MHz, $CDCl_3$)

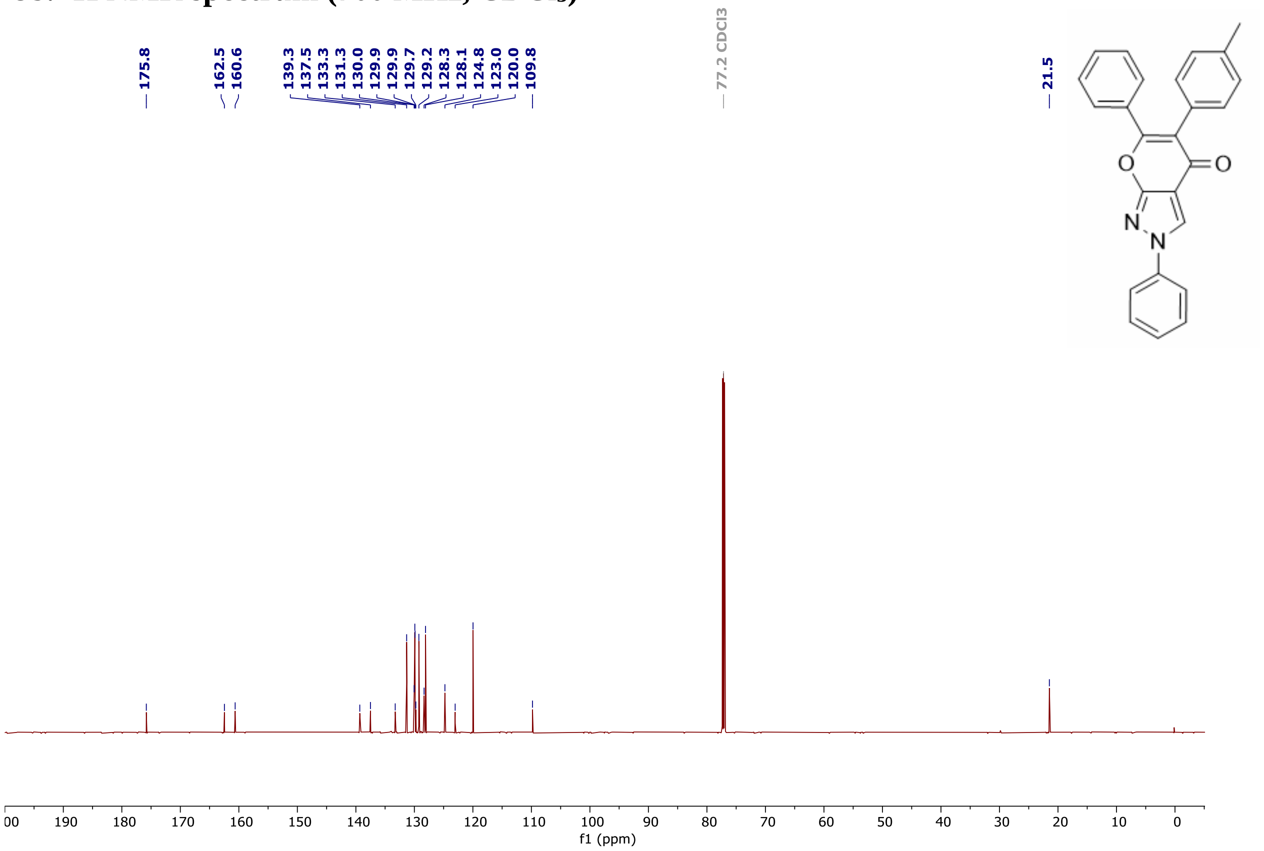

Figure S59. 5-(4-Methylphenyl)-2,6-diphenylpyrano[2,3-*c*]pyrazol-4(2*H*)-one 8b. ^{13}C NMR spectrum (176 MHz, $CDCl_3$)

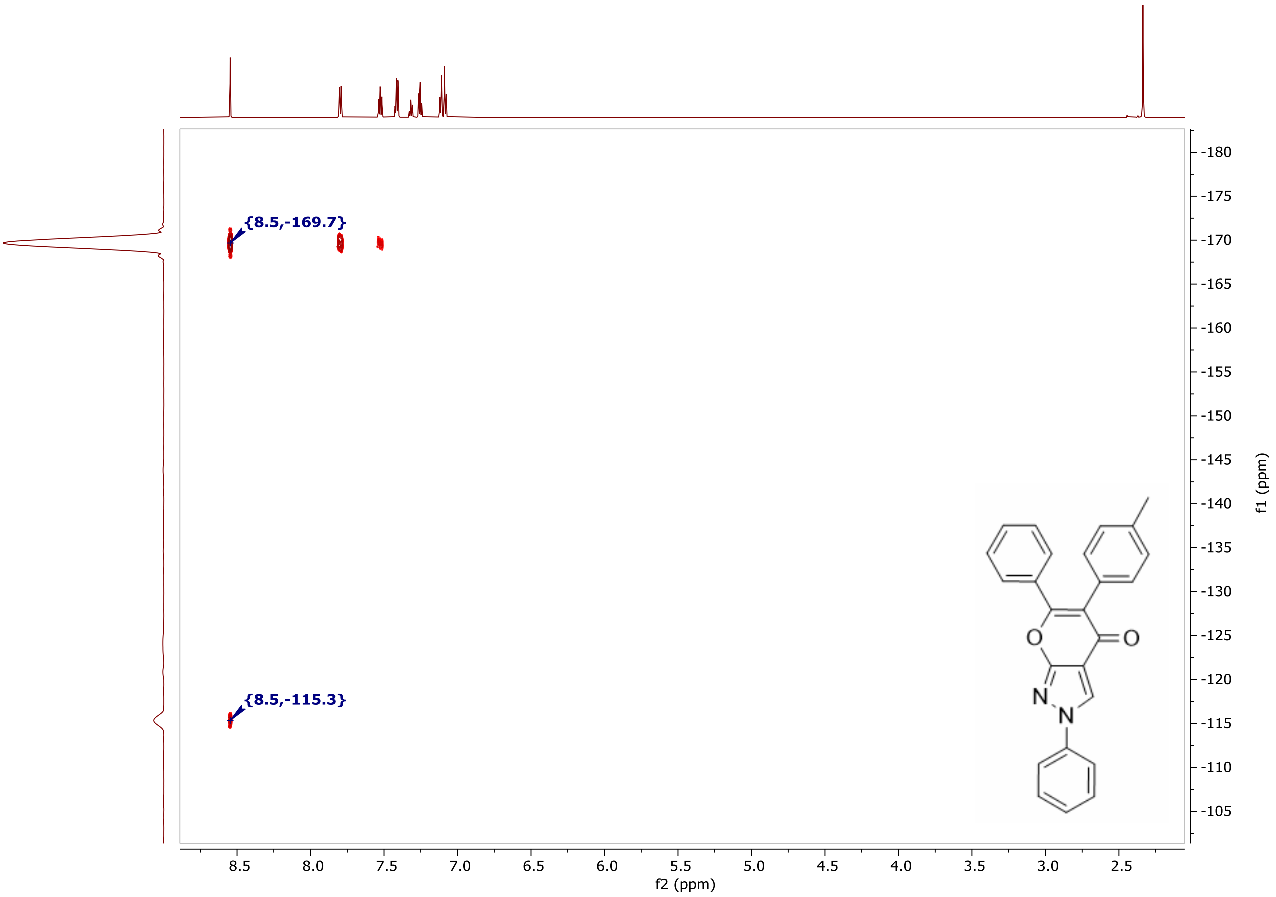

Figure S60. 5-(4-Methylphenyl)-2,6-diphenylpyrano[2,3-*c*]pyrazol-4(2*H*)-one 8b. ^{1}H-^{15}N HMBC NMR spectrum (71 MHz, $CDCl_3$)

Compound Spectrum SmartFormula Report

Analysis Info

Analysis Name	D:\Data\AUM-223.d	Acquisition Date	8/15/2023 6:27:50 PM
Method	DirectInfusion_TuneLow_pos.m	Operator	hplc
Sample Name	AUM-223	Instrument	micrOTOF-Q III 8228888.20448
Comment	AB		

Acquisition Parameter

Source Type	ESI	Ion Polarity	Positive	Set Nebulizer	0.4 Bar
Focus	Not active	Set Capillary	4500 V	Set Dry Heater	180 °C
Scan Begin	50 m/z	Set End Plate Offset	-500 V	Set Dry Gas	4.0 l/min
Scan End	1000 m/z	Set Collision Cell RF	140.0 Vpp	Set Divert Valve	Waste

#	RT [min]	Area	Int. Type	I	S/N	Chromatogram	Max. m/z	FWHM [min]
n.a.	2.7	n.a.	Single spectrum	n.a.	n.a.	n.a.	401.1262	n.a.

+MS, 2.7min #161

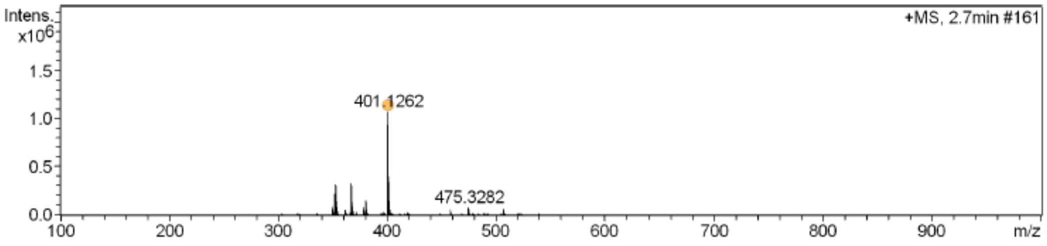

Meas. m/z	#	Ion Formula	m/z	err [ppm]	mSigma	# Sigma	Score	rdb	e¯ Conf	N-Rule
401.1262	1	C25H18N2NaO2	401.1260	0.3	44.1	2	100.00	17.5	even	ok

Figure S61. 5-(4-Methylphenyl)-2,6-diphenylpyrano[2,3-*c*]pyrazol-4(2*H*)-one 8b. HRMS (ESI)

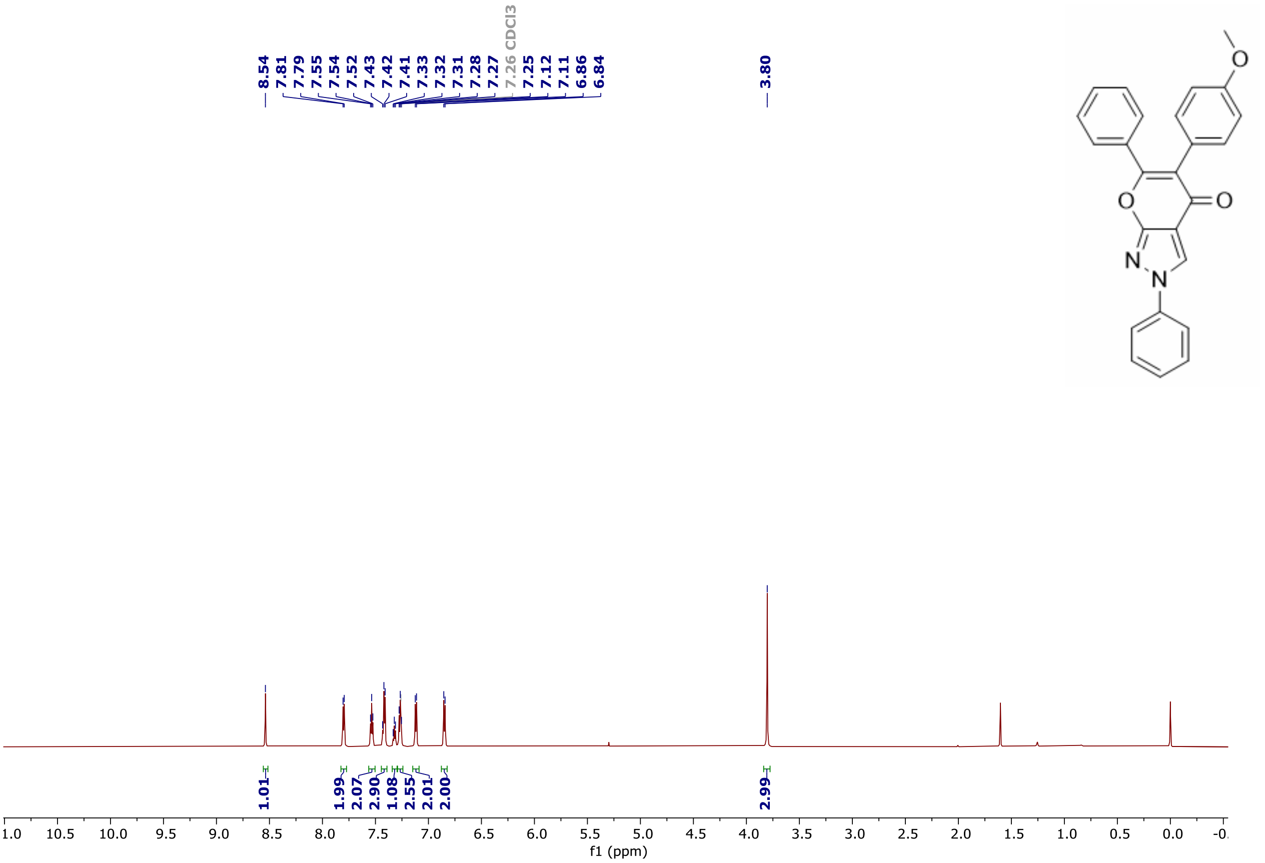

Figure S62. 5-(4-Methoxyphenyl)-2,6-diphenylpyrano[2,3-*c*]pyrazol-4(2*H*)-one 8c. ^{1}H NMR spectrum (700 MHz, $CDCl_3$)

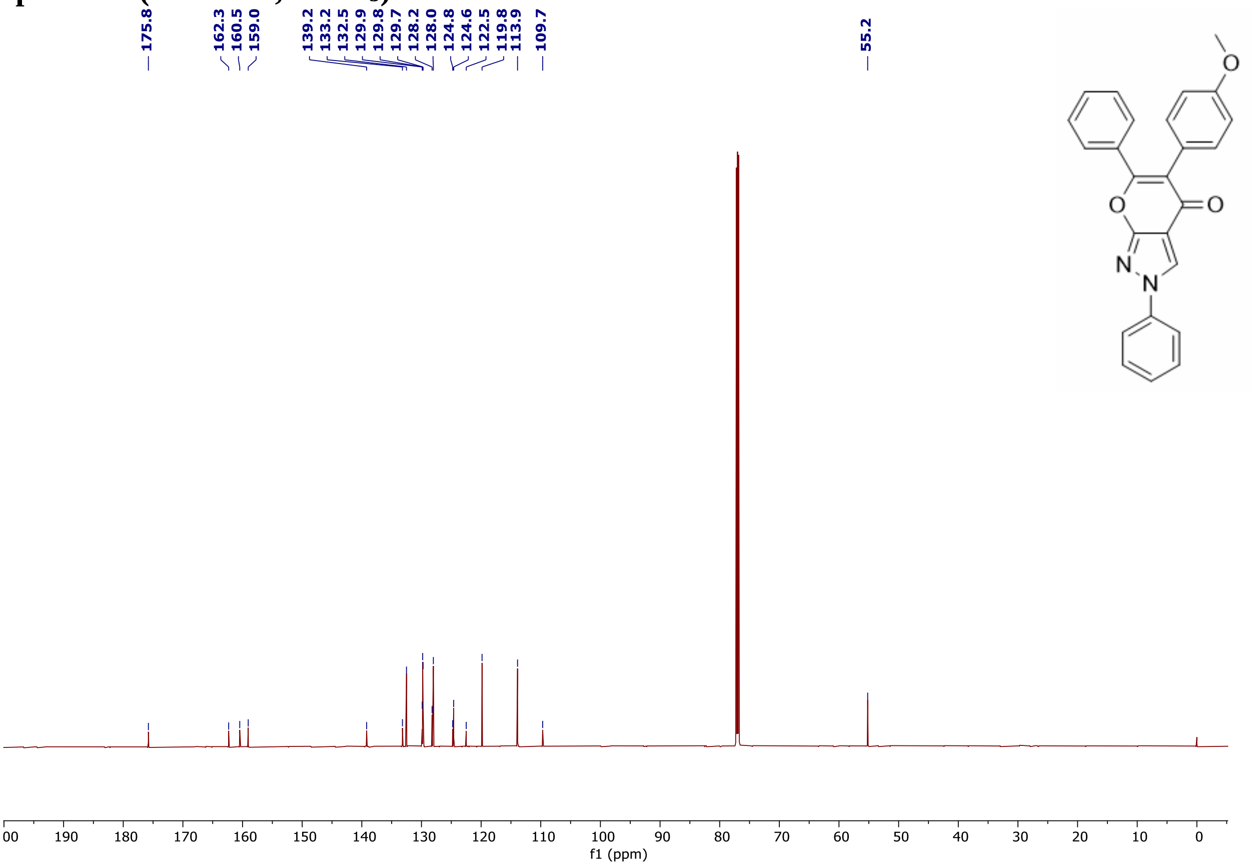

Figure S63. 5-(4-Methoxyphenyl)-2,6-diphenylpyrano[2,3-*c*]pyrazol-4(2*H*)-one 8c. ^{13}C NMR spectrum (176 MHz, $CDCl_3$)

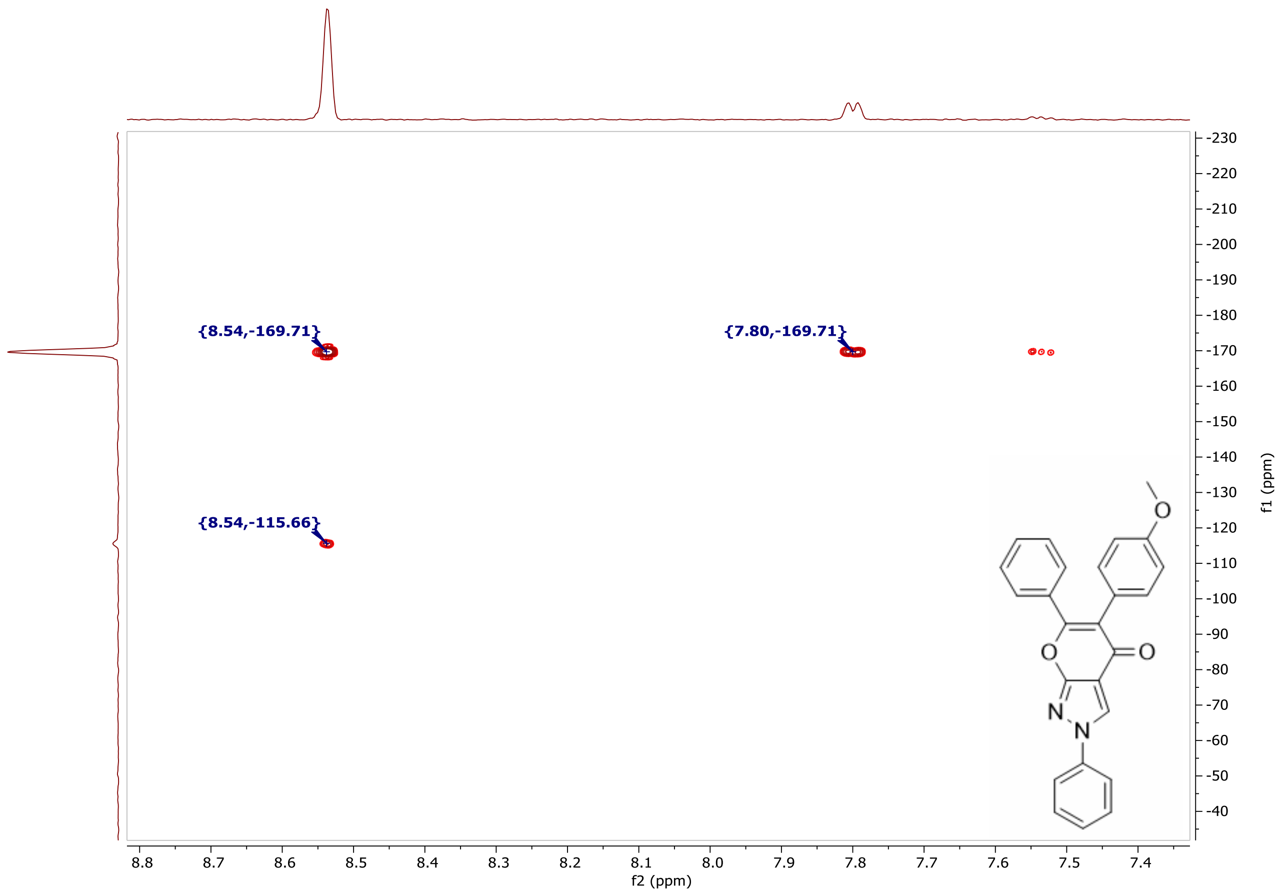

Figure S64 5-(4-Methoxyphenyl)-2,6-diphenylpyrano[2,3-*c*]pyrazol-4(2*H*)-one 8c. ^{1}H-^{15}N HMBC NMR spectrum (71 MHz, $CDCl_3$)

Compound Spectrum SmartFormula Report

Analysis Info

Analysis Name	D:\Data\AUM-225.d	Acquisition Date	8/15/2023 6:47:38 PM
Method	DirectInfusion_TuneLow_pos.m	Operator	hplc
Sample Name	AUM-225	Instrument	micrOTOF-Q III 8228888.20448
Comment	AB		

Acquisition Parameter

Source Type	ESI	Ion Polarity	Positive	Set Nebulizer	0.4 Bar
Focus	Not active	Set Capillary	4500 V	Set Dry Heater	180 °C
Scan Begin	50 m/z	Set End Plate Offset	-500 V	Set Dry Gas	4.0 l/min
Scan End	1000 m/z	Set Collision Cell RF	140.0 Vpp	Set Divert Valve	Waste

#	RT [min]	Area	Int. Type	I	S/N	Chromatogram	Max. m/z	FWHM [min]
n.a.	3.9	n.a.	Single spectrum	n.a.	n.a.	n.a.	417.1208	n.a.

+MS, 3.9min #233

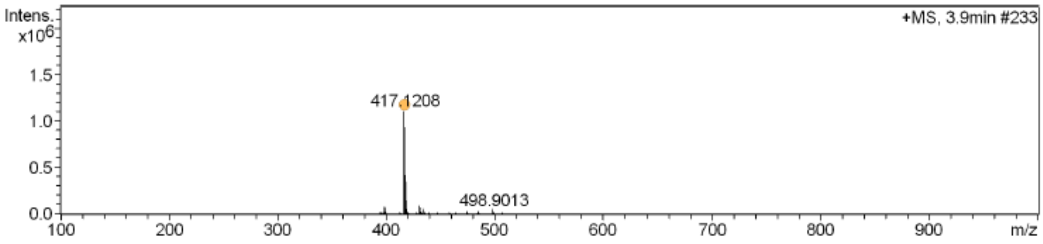

Meas. m/z	#	Ion Formula	m/z	err [ppm]	mSigma	# Sigma	Score	rdb	e⁻ Conf	N-Rule
417.1208	1	C25H18N2NaO3	417.1210	-0.5	52.8	1	100.00	17.5	even	ok

Figure S65. 5-(4-Methoxyphenyl)-2,6-diphenylpyrano[2,3-*c*]pyrazol-4(2*H*)-one 8c. HRMS (ESI)

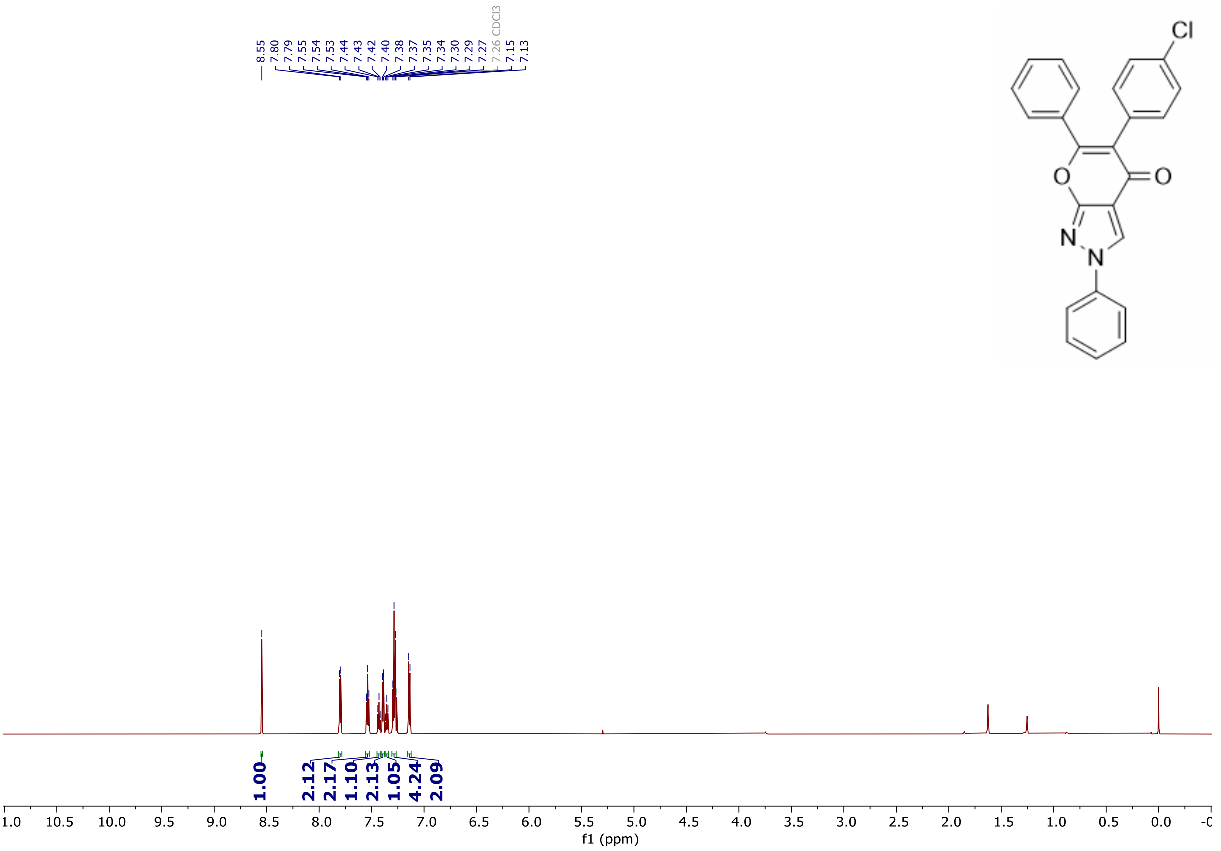

Figure S66. 5-(4-Chlorophenyl)-2,6-diphenylpyrano[2,3-*c*]pyrazol-4(2*H*)-one 8d. ^{1}H NMR spectrum (700 MHz, CDCl$_3$)

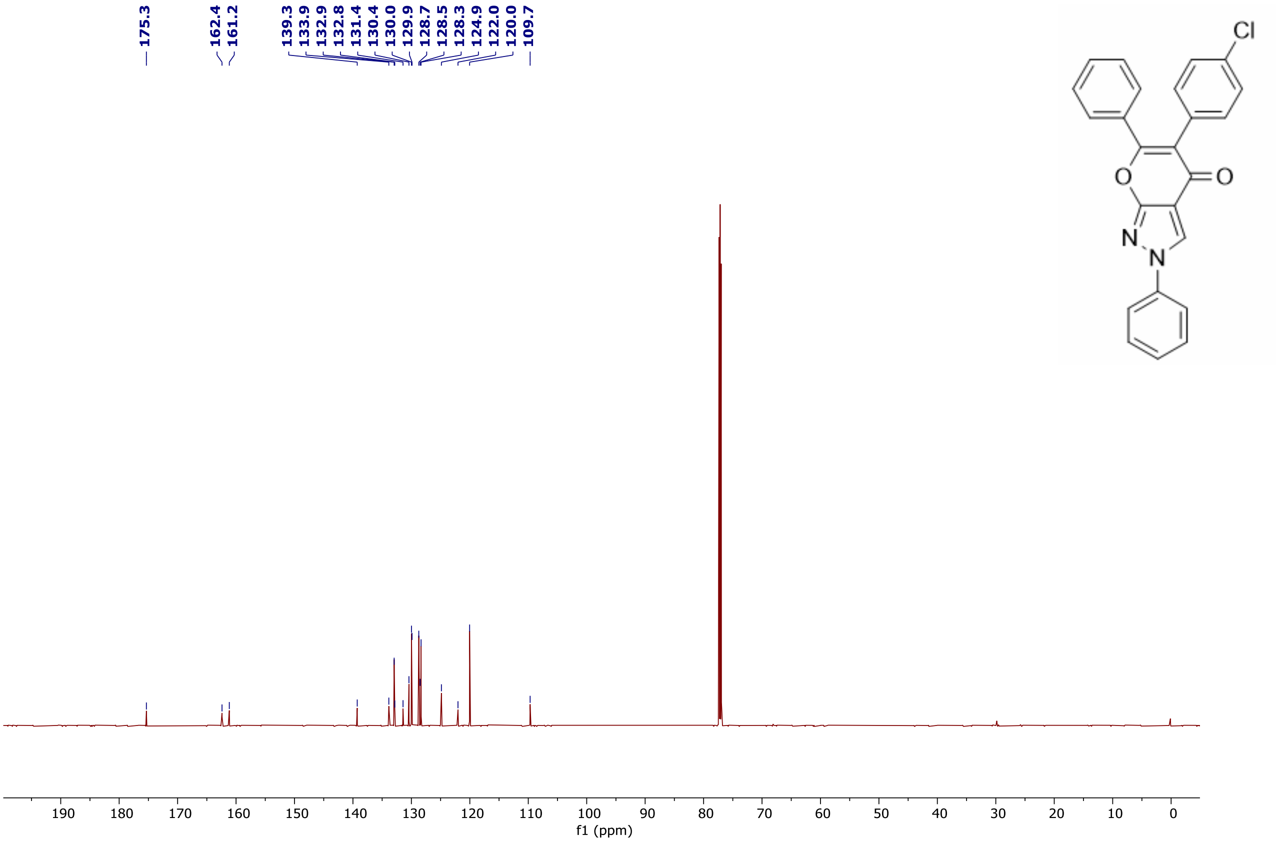

Figure S67. 5-(4-Chlorophenyl)-2,6-diphenylpyrano[2,3-*c*]pyrazol-4(2*H*)-one 8d. ^{13}C NMR spectrum (176 MHz, CDCl$_3$)

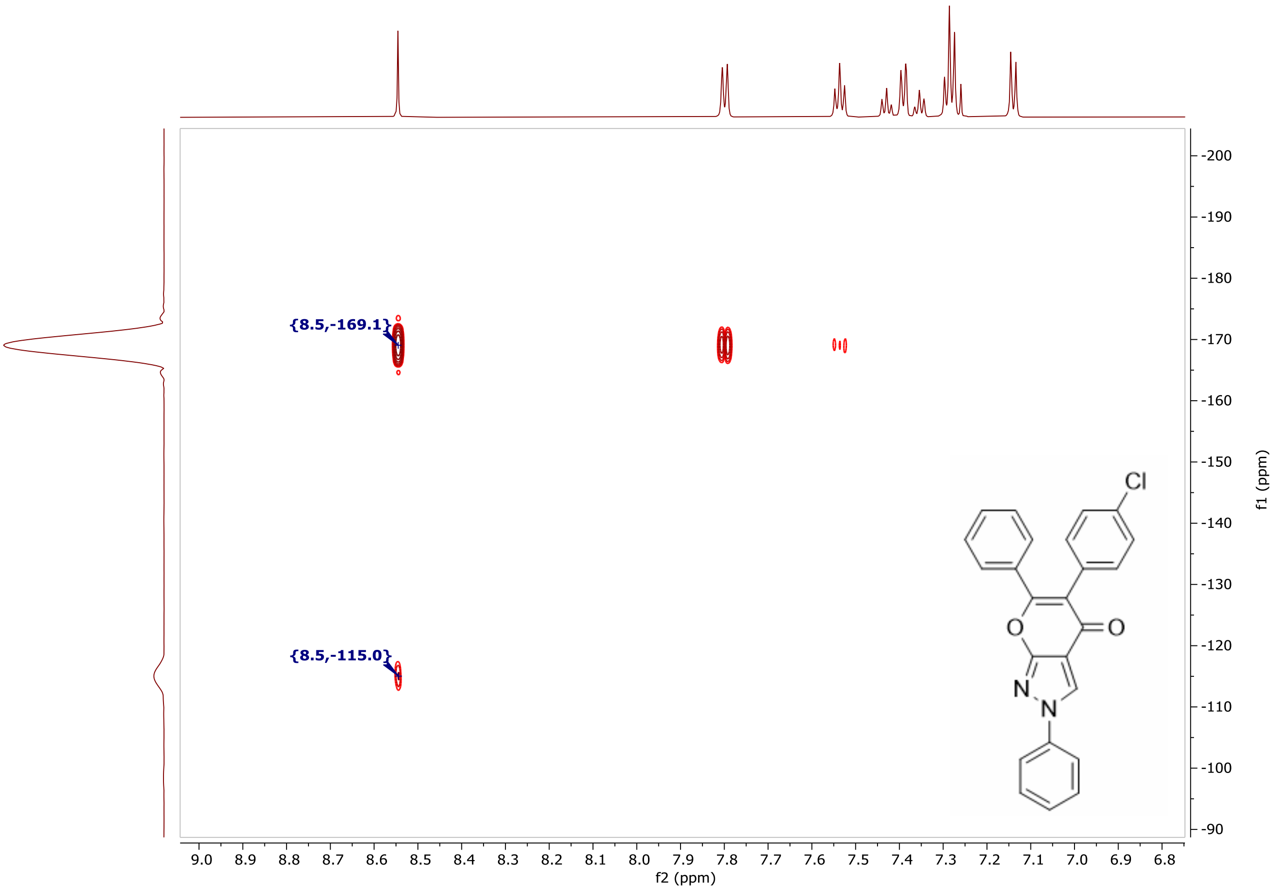

Figure S68 5-(4-Chlorophenyl)-2,6-diphenylpyrano[2,3-*c*]pyrazol-4(2*H*)-one 8d. ^{1}H-^{15}N HMBC NMR spectrum (71 MHz, $CDCl_3$)

Compound Spectrum SmartFormula Report

Analysis Info

Analysis Name	D:\Data\AUM-224.d	Acquisition Date	8/15/2023 6:38:00 PM
Method	DirectInfusion_TuneLow_pos.m	Operator	hplc
Sample Name	AUM-224	Instrument	microTOF-Q III 8228888.20448
Comment	AB		

Acquisition Parameter

Source Type	ESI	Ion Polarity	Positive	Set Nebulizer	0.4 Bar
Focus	Not active	Set Capillary	4500 V	Set Dry Heater	180 °C
Scan Begin	50 m/z	Set End Plate Offset	-500 V	Set Dry Gas	4.0 l/min
Scan End	1000 m/z	Set Collision Cell RF	140.0 Vpp	Set Divert Valve	Waste

#	RT [min]	Area	Int. Type	I	S/N	Chromatogram	Max. m/z	FWHM [min]
n.a.	4.6	n.a.	Single spectrum	n.a.	n.a.	n.a.	421.0711	n.a.

+MS, 4.6min #277

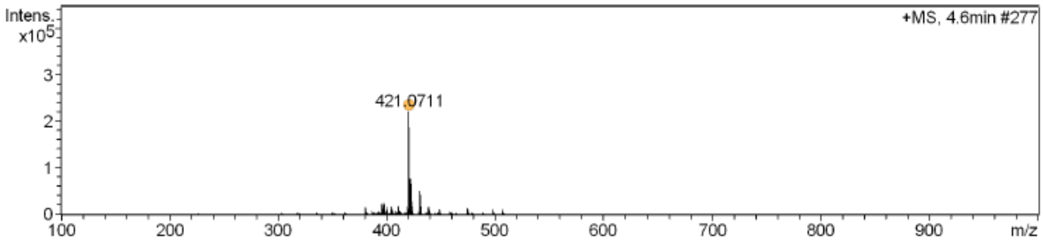

Meas. m/z	#	Ion Formula	m/z	err [ppm]	mSigma	# Sigma	Score	rdb	e⁻ Conf	N-Rule
421.0711	1	C24H15ClN2NaO2	421.0714	-0.7	5.3	1	100.00	17.5	even	ok

Figure S69. 5-(4-Chlorophenyl)-2,6-diphenylpyrano[2,3-*c*]pyrazol-4(2*H*)-one 8d. HRMS (ESI)

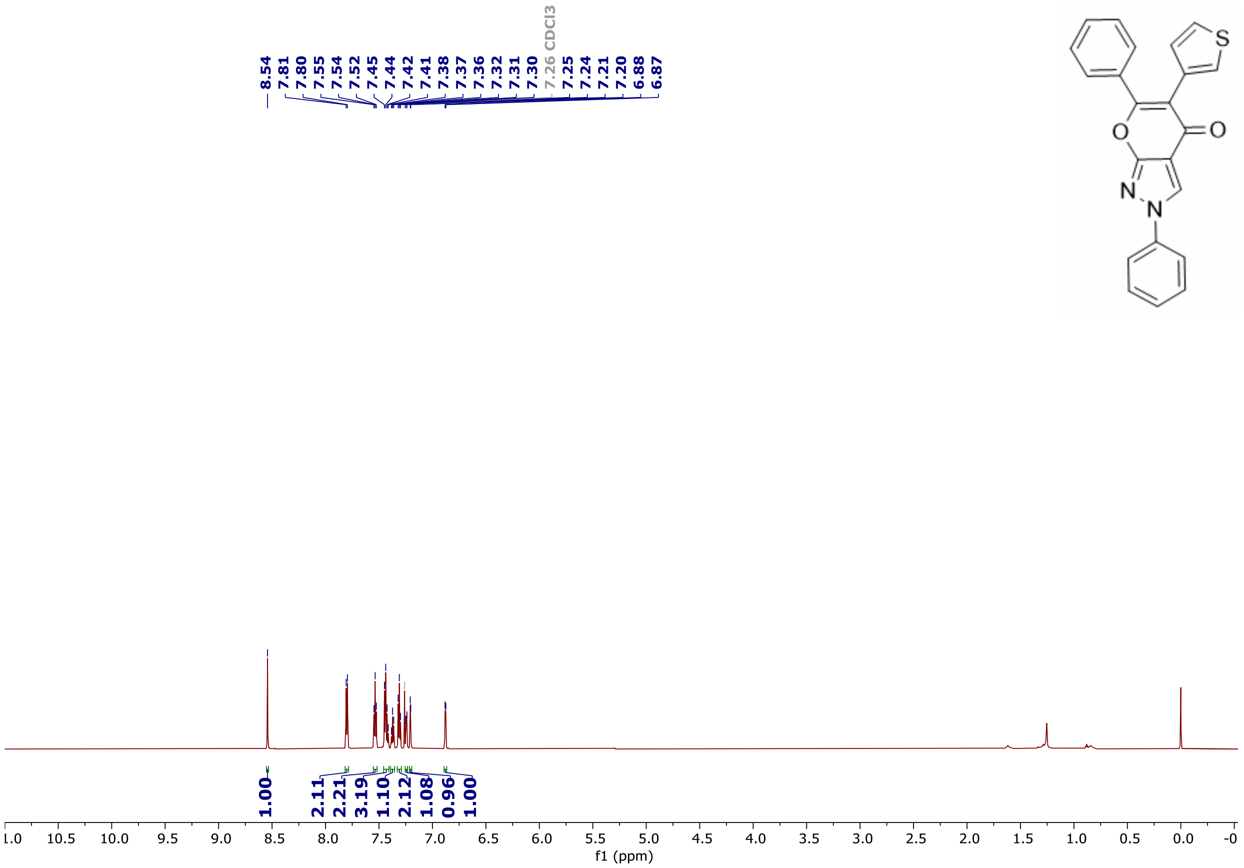

Figure S70. 2,6-Diphenyl-5-(thiophen-3-yl)pyrano[2,3-*c*]pyrazol-4(2*H*)-one 8e. ^{1}H NMR spectrum (700 MHz, $CDCl_3$)

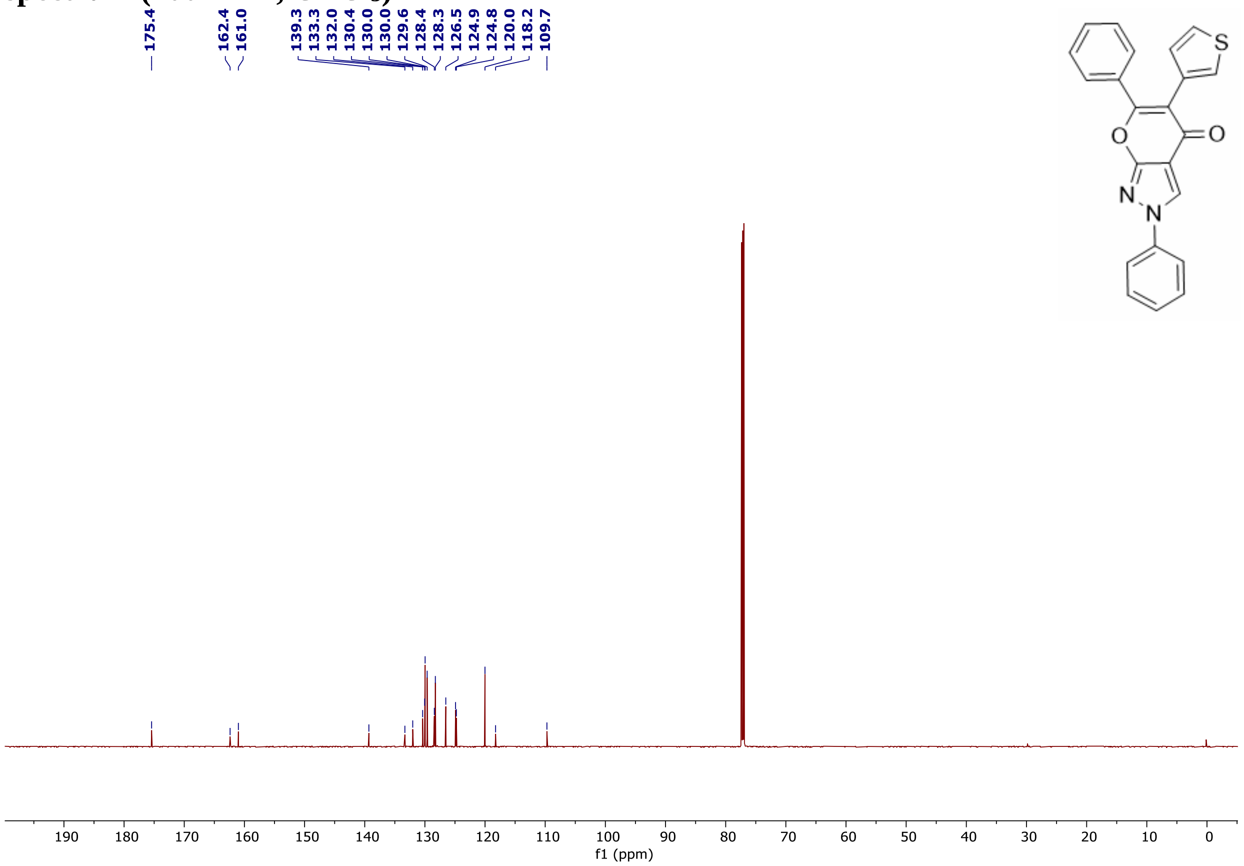

Figure S71. 2,6-Diphenyl-5-(thiophen-3-yl)pyrano[2,3-*c*]pyrazol-4(2*H*)-one 8e. ^{13}C NMR spectrum (176 MHz, $CDCl_3$)

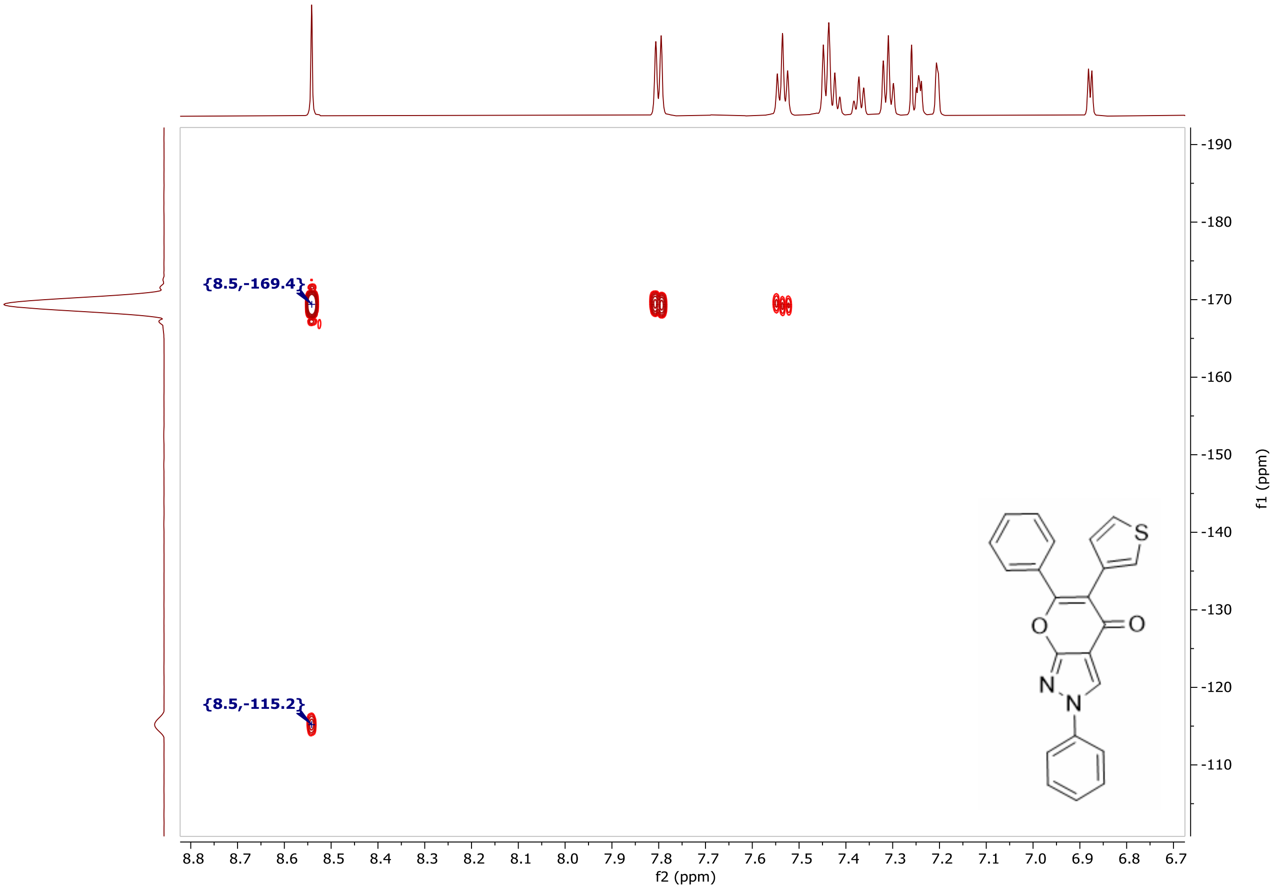

Figure S72 2,6-Diphenyl-5-(thiophen-3-yl)pyrano[2,3-*c*]pyrazol-4(2*H*)-one 8e. ^{1}H-^{15}N HMBC NMR spectrum (71 MHz, $CDCl_3$)

Compound Spectrum SmartFormula Report

Analysis Info

Analysis Name	D:\Data\AUM-238.d	Acquisition Date	8/15/2023 7:08:12 PM
Method	DirectInfusion_TuneLow_pos.m	Operator	hplc
Sample Name	AUM-238	Instrument	micrOTOF-Q III 8228888.20448
Comment	AB		

Acquisition Parameter

Source Type	ESI	Ion Polarity	Positive	Set Nebulizer	0.4 Bar
Focus	Not active	Set Capillary	4500 V	Set Dry Heater	180 °C
Scan Begin	50 m/z	Set End Plate Offset	-500 V	Set Dry Gas	4.0 l/min
Scan End	1000 m/z	Set Collision Cell RF	140.0 Vpp	Set Divert Valve	Waste

#	RT [min]	Area	Int. Type	I	S/N	Chromatogram	Max. m/z	FWHM [min]
n.a.	5.1	n.a.	Single spectrum	n.a.	n.a.	n.a.	393.0669	n.a.

+MS, 5.1min #303

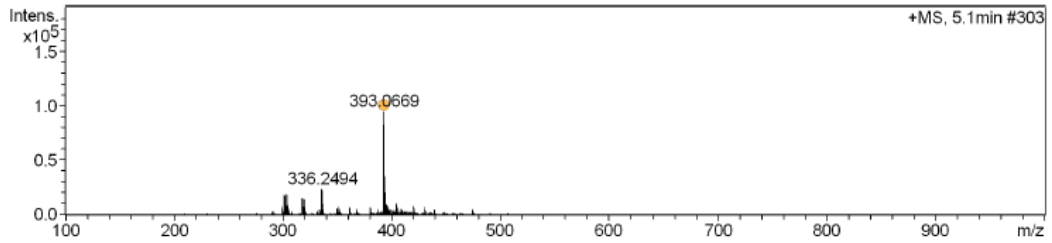

Meas. m/z	#	Ion Formula	m/z	err [ppm]	mSigma	# Sigma	Score	rdb	e⁻ Conf	N-Rule
393.0669	1	C22H14N2NaO2S	393.0668	-0.3	2.6	1	100.00	16.5	even	ok

Figure S73. 2,6-Diphenyl-5-(thiophen-3-yl)pyrano[2,3-*c*]pyrazol-4(2*H*)-one 8e. HRMS (ESI)

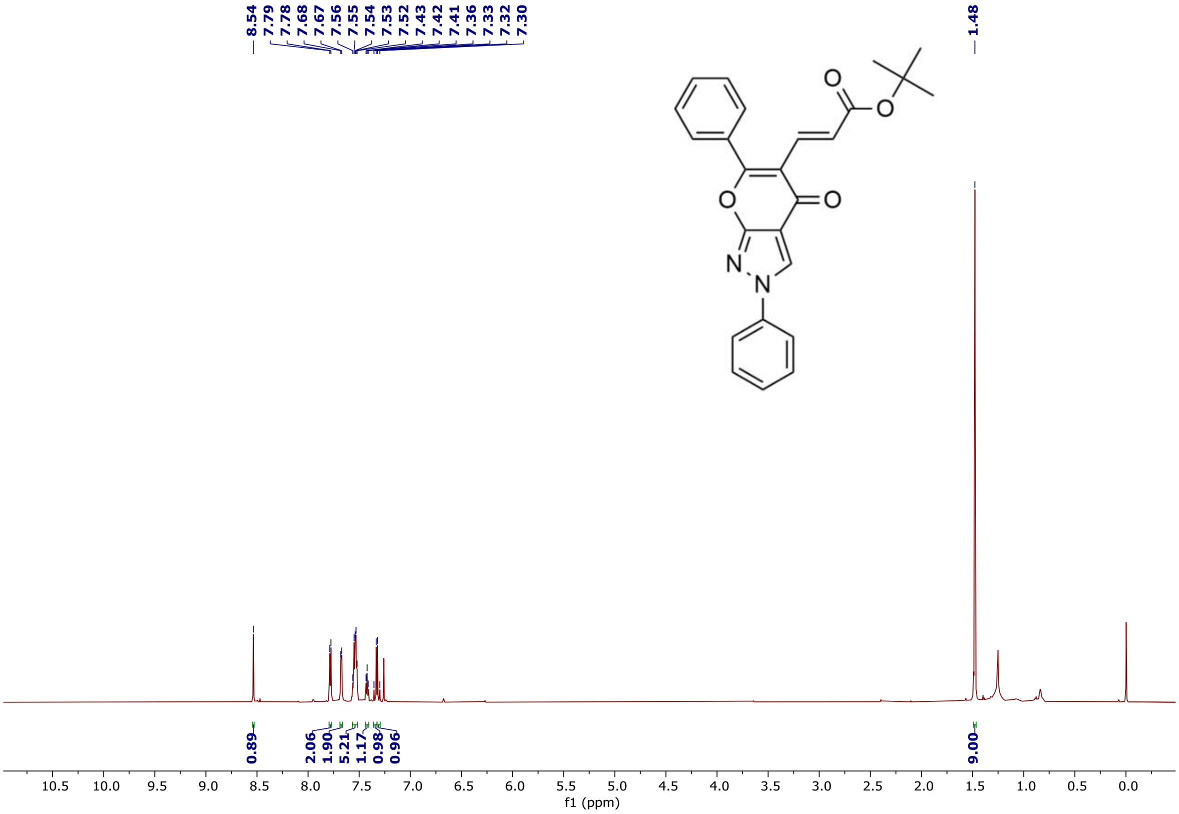

Figure S74. ***tert*-Butyl (2*E*)-3-(4-oxo-2,6-diphenyl-2,4-dihydropyrano[2,3-*c*]pyrazol-5-yl)prop-2-enoate 8f. ^{1}H NMR spectrum (700 MHz, $CDCl_3$)**

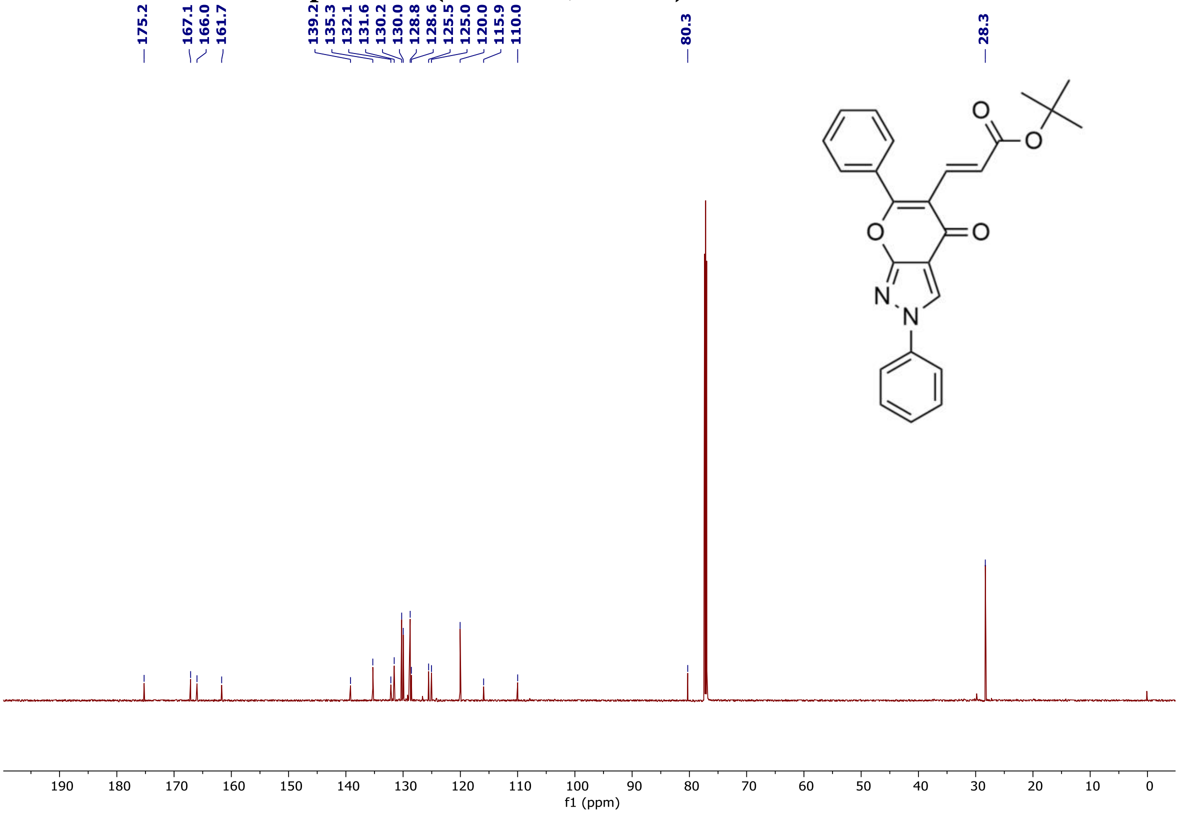

Figure S75. ***tert*-Butyl (2*E*)-3-(4-oxo-2,6-diphenyl-2,4-dihydropyrano[2,3-*c*]pyrazol-5-yl)prop-2-enoate 8f. ^{13}C NMR spectrum (176 MHz, $CDCl_3$)**

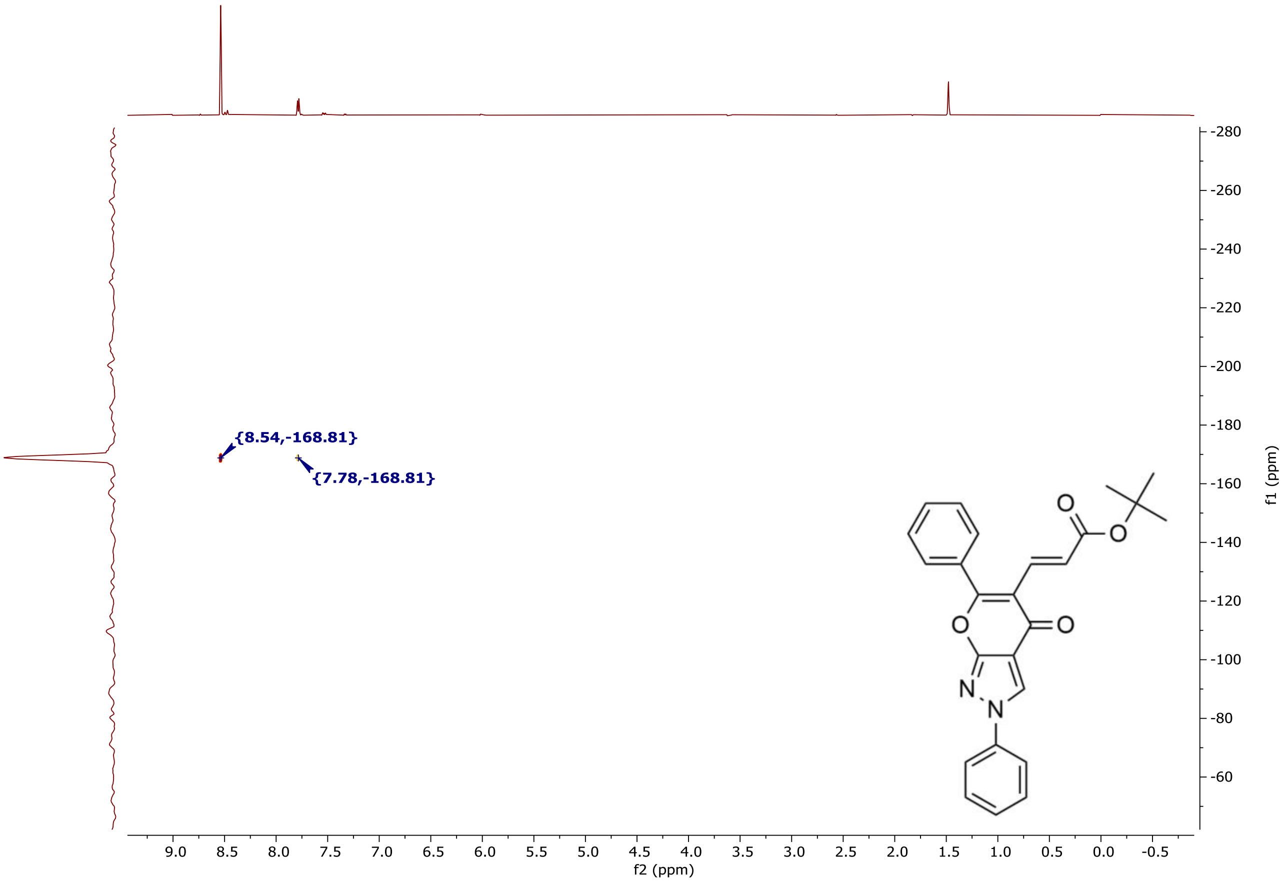

Figure S76. ***tert*-Butyl (2*E*)-3-(4-oxo-2,6-diphenyl-2,4-dihydropyrano[2,3-*c*]pyrazol-5-yl)prop-2-enoate 8f. ^{1}H-^{15}N HMBC NMR spectrum (71 MHz, $CDCl_3$)**

Compound Spectrum SmartFormula Report

Analysis Info

Analysis Name	D:\Data\AUM-231.d	Acquisition Date	6/7/2023 6:02:03 PM
Method	DirectInfusion_TuneLow_pos.m	Operator	hplc
Sample Name	AUM-231	Instrument	microTOF-Q III 8228888.20448
Comment	AB		

Acquisition Parameter

Source Type	ESI	Ion Polarity	Positive	Set Nebulizer	0.4 Bar
Focus	Not active	Set Capillary	4500 V	Set Dry Heater	180 °C
Scan Begin	50 m/z	Set End Plate Offset	-500 V	Set Dry Gas	4.0 l/min
Scan End	1000 m/z	Set Collision Cell RF	140.0 Vpp	Set Divert Valve	Waste

#	RT [min]	Area	Int. Type	I	S/N	Chromatogram	Max. m/z	FWHM [min]
n.a.	15.3	n.a.	Single spectrum	n.a.	n.a.	n.a.	437.1473	n.a.

+MS, 15.3min #920

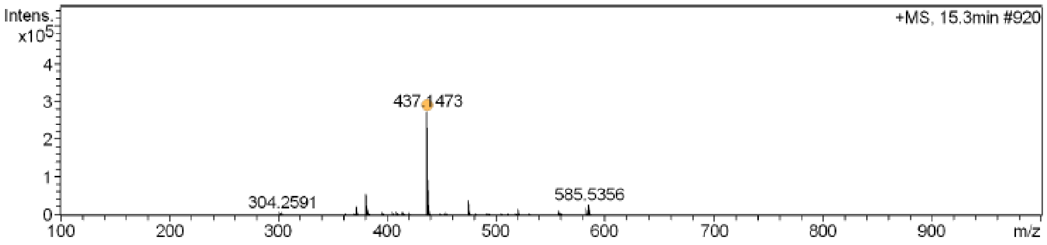

Meas. m/z	#	Ion Formula	m/z	err [ppm]	mSigma	# Sigma	Score	rdb	e⁻ Conf	N-Rule
437.1473	1	C25H22N2NaO4	437.1472	-0.4	3.9	1	100.00	15.5	even	ok

Figure S77. *tert*-Butyl (2*E*)-3-(4-oxo-2,6-diphenyl-2,4-dihydropyrano[2,3-*c*]pyrazol-5-yl)prop-2-enoate 8f. HRMS (ESI)

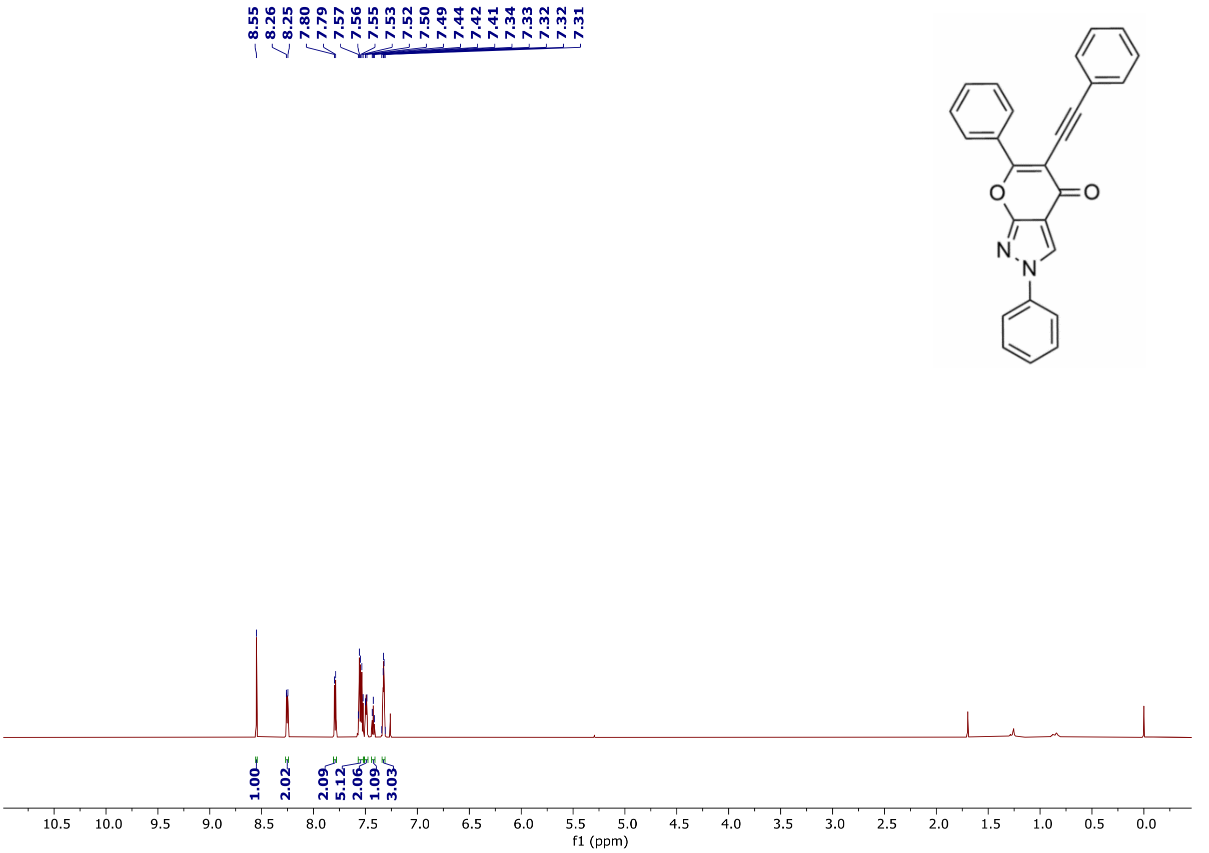

Figure S78. 2,6-Diphenyl-5-(phenylethynyl)pyrano[2,3-*c*]pyrazol-4(2*H*)-one 8g. ^{1}H NMR spectrum (700 MHz, $CDCl_3$)

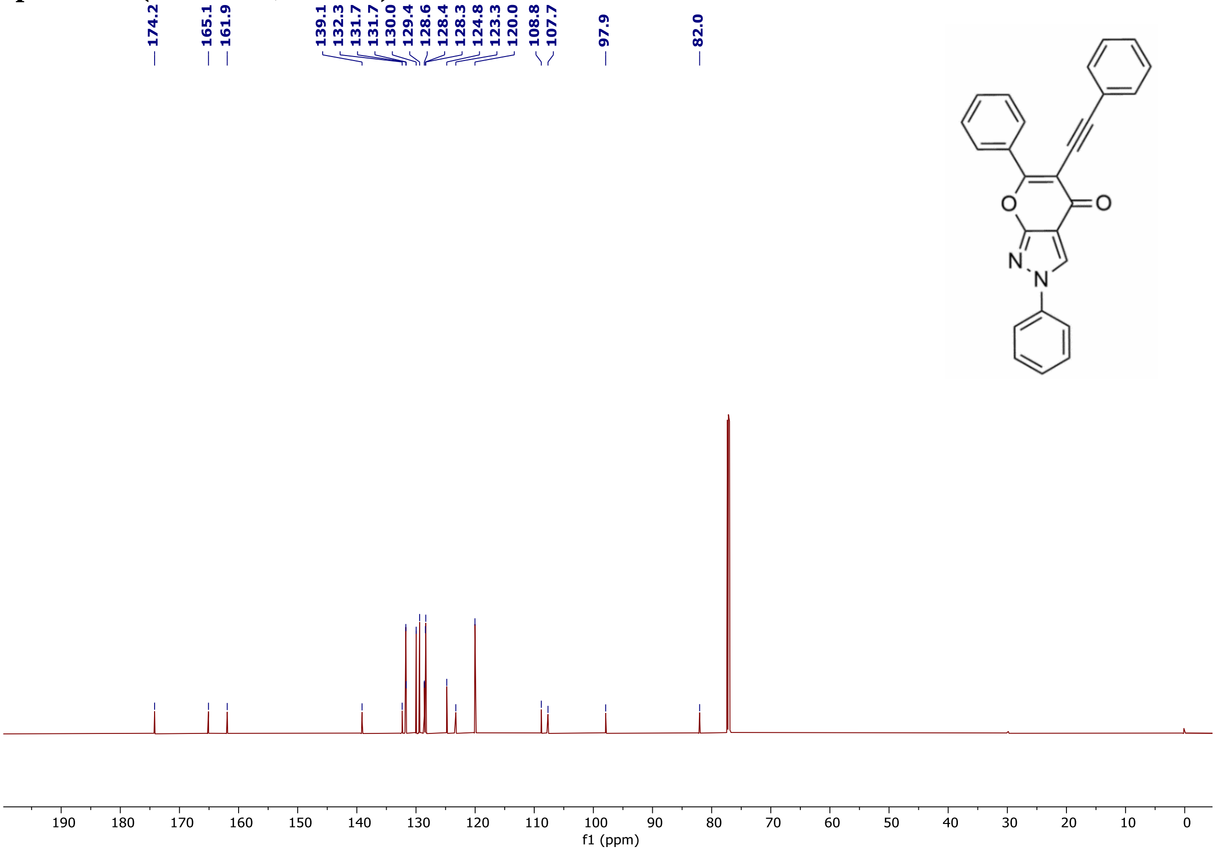

Figure S79. 2,6-Diphenyl-5-(phenylethynyl)pyrano[2,3-*c*]pyrazol-4(2*H*)-one 8g. ^{13}C NMR spectrum (176 MHz, $CDCl_3$)

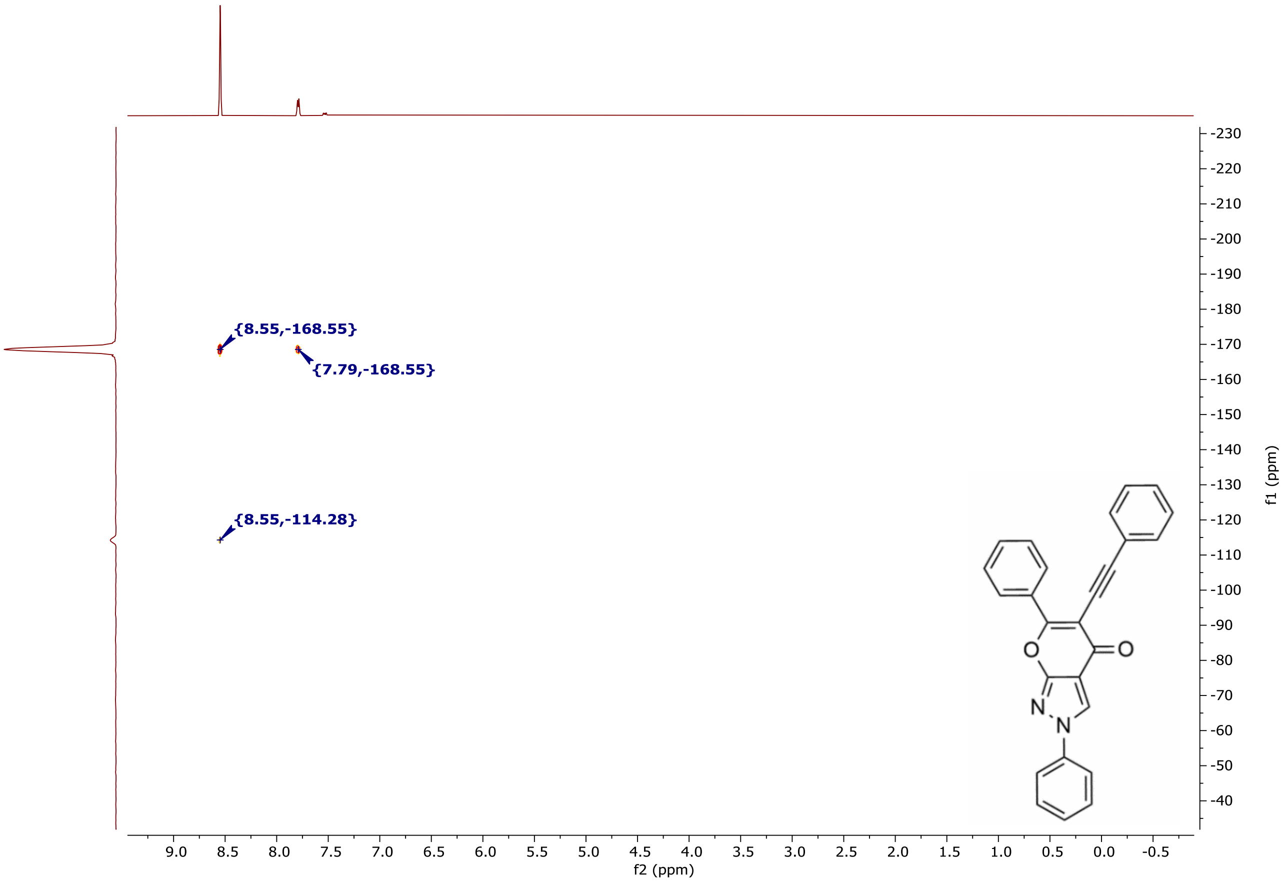

Figure S80. 2,6-Diphenyl-5-(phenylethynyl)pyrano[2,3-*c*]pyrazol-4(2*H*)-one 8g. ^{1}H-^{15}N HMBC NMR spectrum (71 MHz, $CDCl_3$)

Compound Spectrum SmartFormula Report

Analysis Info

Analysis Name	D:\Data\AUM-230.d	Acquisition Date	6/7/2023 5:38:02 PM
Method	DirectInfusion_TuneLow_pos.m	Operator	hplc
Sample Name	AUM-230	Instrument	micrOTOF-Q III 8228888.20448
Comment	AB		

Acquisition Parameter

Source Type	ESI	Ion Polarity	Positive	Set Nebulizer	0.4 Bar
Focus	Not active	Set Capillary	4500 V	Set Dry Heater	180 °C
Scan Begin	50 m/z	Set End Plate Offset	-500 V	Set Dry Gas	4.0 l/min
Scan End	1000 m/z	Set Collision Cell RF	140.0 Vpp	Set Divert Valve	Waste

#	RT [min]	Area	Int. Type	I	S/N	Chromatogram	Max. m/z	FWHM [min]
n.a.	6.1	n.a.	Single spectrum	n.a.	n.a.	n.a.	411.1101	n.a.

+MS, 6.1min #367

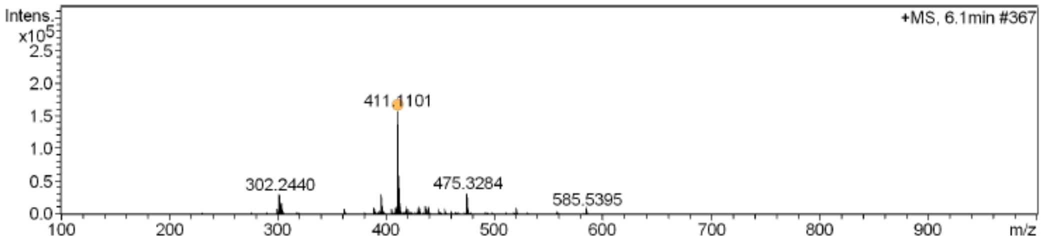

Meas. m/z	#	Ion Formula	m/z	err [ppm]	mSigma	# Sigma	Score	rdb	e¯ Conf	N-Rule
411.1101	1	C26H16N2NaO2	411.1104	0.7	2.2	1	100.00	19.5	even	ok

Figure S81. 2,6-Diphenyl-5-(phenylethynyl)pyrano[2,3-*c*]pyrazol-4(2*H*)-one 8g. HRMS (ESI)

References

1. Dolomanov, O.V., Bourhis, L.J., Gildea, R.J, Howard, J.A.K. & Puschmann, H. (2009), *J. Appl. Cryst.* 42, 339-341.
2. Sheldrick, G.M. (2015). *Acta Cryst.* A71, 3-8.
3. Bourhis, L.J., Dolomanov, O.V., Gildea, R.J., Howard, J.A.K., Puschmann, H. (2015). *Acta Cryst.* A71, 59-75.
